# Instantaneous formation of interstellar minerals and mineral quantum dots†

**DOI:** 10.1039/d5ra01088h

**Published:** 2025-04-17

**Authors:** Arijit Roy, Surendra V. Singh, R. Ramachandran, J. K. Meka, M. Ambresh, T. Vijay, P. Janardhan, V. Jayaram, V. Venkatraman, A. Das, H. Hill, Anil Bhardwaj, N. J. Mason, B. Sivaraman

**Affiliations:** a Physical Research Laboratory Ahmedabad 380009 India arijit@prl.res.in bhala@prl.res.in; b Institute for Astronomy, Space and Earth Sciences Kolkata 700054 India; c Indian Institute of Science Bengaluru 560012 India; d Indian Institute of Technology-Gandhinagar (IIT-Gn) Gandhinagar 382055 India; e Space Physics Laboratory, Vikram Sarabhai Space Centre Thiruvananthapuram 695022 India; f International Space University (ISU) Strasbourg France; g The School of Physics and Astronomy, University of Kent Canterbury CT2 7NZ UK

## Abstract

Our understanding of the formation pathways of interstellar mineral dust is still evolving. This study investigated the formation of astrophysical mineral dust, such as olivine, by shock processing. Low-velocity (∼1.8 km s^−1^) interstellar shock conditions were simulated using high-intensity shock tubes. These conditions enabled the examination of various cosmic mineral dust precursors such as the mixtures of Mg, Fe and SiO_2_ under a shock strength of approximately 5.6 M and temperatures around 7300 K. Analysis of the processed samples revealed the presence of Mg-rich olivine, forsterite, MgO quantum dots (QD), and magnetite. These results indicate that shockwaves can rapidly induce dust formation in interstellar space. Furthermore, we demonstrated that shock processing of mineral dust precursors could contribute to the formation of crystalline silicate dust observed in comets and the creation of chondrules, which are observed in chondritic meteorites.

## Abstract 2

Interstellar minerals and mineral quantum dots are formed within milliseconds by shock processing mixtures of Mg, Fe, and SiO_2_.

## Graphical abstract

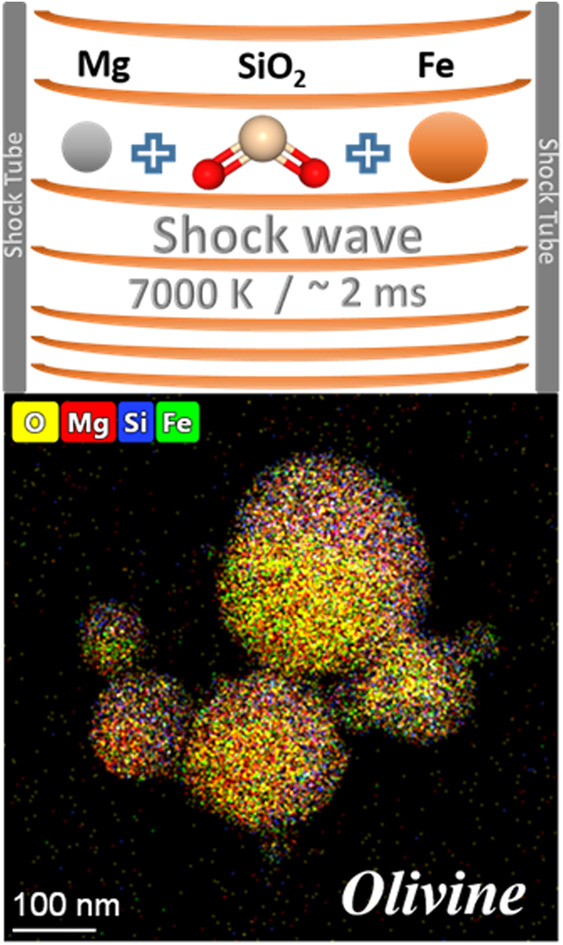

## 1. Introduction

Interstellar dust, which accounts for 1% of the interstellar mass budget, plays a significant role in both the interstellar molecular enrichment process and cosmic radiation balance, while serving as a basic building block of planetesimals. A significant amount of interstellar dust is arguably formed *via* gas-phase condensation processes in winds of evolved stars such as the Asymptotic Giant Branch (AGB) star.^1,2^ The elemental abundance of these stars greatly affects the chemical compositions of these dust grains, especially the C/O abundance ratio, as these two elements have the highest abundances after hydrogen and helium in these stars. In the case of C/O < 1, the dust grains are mostly oxides like olivine, pyroxene, spinel *etc.*; in the case of C/O > 1, dust grains are carbonaceous like SiC, TiC, C_60_, and other carbon allotropes.^3^

Silicate-type mineral dust has been detected for decades in different astrophysical regions using their IR spectral features around 10 μm and 20 μm that are attributed to Si–O stretching and O–Si–O bending vibrations.^4^ These spectral signatures have also been observed in several comets,^5,6^ asteroids,^7,8^ and Interplanetary Dust Particles (IDP).^9^ While crystalline silicate dust makes up less than 5% of the interstellar silicate dust population,^10^ the majority of observed interstellar silicate dust grains are amorphous pyroxene or olivine.^11,12^ The backbone of silicate dust is the [SiO_4_]^4–^ tetrahedron, which can link with other SiO_4_ tetrahedra or abundant cations like Mg^2+^ and Fe^2+^ to form different minerals such as pyroxene and olivine.^4,13^

The formation of interstellar silicate dust grains is a complex process that is still being investigated. Various experimental and theoretical methods have been used in an attempt to understand the formation pathways of these grains. Some of these proposed formation pathways include melt and quench,^14^ sol–gel processing,^15^ crystal growth,^16,17^ gas phase condensation,^18,19^ laser pyrolysis,^20,21^ and ion-induced processing.^22^ Methods such as the sol–gel technique, and the melt and quench technique are dedicated mainly to the production of bulk grains in thermodynamic equilibrium. The grains produced in these methods are homogeneous and mostly used for the measurement of optical constants.^23^ However, this production technique does not mimic the real scenario in which dust grains originate in stellar envelopes.^23^ Dust grains produced in conventional gas phase condensation techniques are mostly amorphous, and further heating is required to turn them into the crystalline form.^24,25^

Shock waves play a crucial role as energy sources in the interstellar medium (ISM). Low-velocity shock waves (10 kms^−1^) likely originate from the envelopes of red giant stars^26^ and early-type stars with spectral type O and B.^27^ These shocks compress and heat the gas and dust in their propagating medium, enhancing the chemical complexity of the environment.^28,29^ Astronomical observations of Nova V2891 Cygni has provided evidence of dust formation behind the shock front.^30^ Shock processing of amorphous grains in the early solar nebula has been proposed as a potential mechanism for forming crystalline silicates, which later become incorporated into comets.^31,32^ Additionally, shock-induced flash heating can play a key role in forming chondrules in the primordial solar nebula.^33^

Owing to the importance of shock waves in enriching cosmic dust chemistry, laboratory experiments are required to mimic the interstellar shock processing of cosmic dust analogues and the synthesis of dust grains from their elemental precursors. In recent years, shock tubes have emerged as a tool capable of mimicking extreme interstellar shock conditions in an instant.^34–36^ By utilizing a shock tube, known as High Intensity Shock Tube for Astrochemistry (HISTA), in this work we investigated the low-velocity (1.8 kms^−1^, 5.6 M) shock processing of stoichiometric mixtures of olivine precursor dust analogues, namely Mg + SiO_2_ mixed in equal quantities (1 : 1) referred to as M1, M1 + Fe (1 : 1) referred to as M2, and Fe + SiO_2_ (1 : 1) referred as M3. The precursor mixtures were subjected to shock waves with a reflected shock temperature of about 7300 K achieved for 2 ms. The solid residues obtained after the shock processing were collected and analyzed using different analytical techniques, including Attenuated Total Reflectance (ATR)-FTIR Spectroscopy, X-ray Diffraction (XRD), Field Emission Scanning Electron Microscopy (FE-SEM), High-Resolution Transmission Electron Microscopy (HR-TEM), and Electron Dispersive X-ray (EDX) spectroscopy.

## 2. Experimental methodology

### 2.1 Shock tube operation

The High-Intensity Shock Tube for Astrochemistry (HISTA) housed at Physical Research Laboratory, Ahmedabad, has been used to perform shock processing of stoichiometric mixtures of interstellar silicate dust analogue samples named M1, M2, and M3. Details of M1, M2, and M3 can be found in Table 1. The powder samples of Mg, Fe and SiO_2_ were procured from Sigma-Aldrich with a purity of about 99.5% on a trace metals basis. HISTA is a 7 m long shock tube with a 2 m long driver section and a 5 m long driven section, and these two sections are separated by a metal (Al) diaphragm. In this work we use He as the driver gas and Ar as the driven gas. A detailed description of HISTA, its working principles and procedure, sample preparation, the Mach number measurement technique, reflection shock temperature and pressure calculation are documented in the supplementary section (Fig. S1 and S2†) and discussed in previously published work.^35,36^ About 0.1 gm of M1, M2 and M3 samples were separately shock processed using a shock wave with Mach 5.6 and reflected shock temperature ∼7300 K for 2 ms. Details of the experimental parameters such as diaphragm bursting pressure (*P*_4_), driven pressure (*P*_1_), Mach Number (*M*), and the estimated reflected shock temperature (*T*_5_) using Rankine–Hugoniot (R–H) jump equations used in this experiment can be found in Table 1.

**Table 1:** Sample details along with experimental parameters

Sample details	Bursting pressure (bar) (He as driver gas)	Driven gas (Ar) pressure (bar)	Mach (M)	Reflected shock temperature (*T*_5_) (K)
M1 = Mg + SiO_2_ (1 : 1)	68.5	0.1	5.6	∼7300
M2 = M1+ Fe (1 : 1)	68.9	0.1	5.6	∼7300
M3 = Fe + SiO_2_ (1 : 1)	67.8	0.1	5.6	∼7300

After the shock processing, the samples were scratched from the inner walls of the HISTA and from its end flange. These collected samples were analysed using Attenuated Total Reflectance (ATR) FTIR Spectroscopy, X-ray Diffraction analysis (XRD), Field Emission Scanning Electron Microscopy (FE-SEM), and High-Resolution Transmission Electron Microscopy (HR-TEM) Technique.

### 2.2 ATR-FTIR spectroscopy

ATR-FTIR spectroscopy of the shocked and unshocked sample was carried out using a diamond attenuated total reflectance module attached to the Nicolet IS50 FTIR spectrometer. Using a Polaris IR source (9600–10 cm^−1^), solid substrate beam splitter (>1000–10 cm^−1^), and a DLaTGS-KBr detector (12 500–350 cm^−1^), we measured MIR to FIR (up to 35 μm) spectra of the samples. The spectrometer's resolution was 2 cm^−1^ and the acquisition time was around 1 minute 32 s.

### 2.3 XRD analysis

We used a Bruker D8 DISCOVER X-ray diffractometer to analyze our samples. For the indexing of the different peaks in the XRD pattern we compared our experimental data with the standard data available in the Crystallographic Open Database (COD), where a standard XRD was generated using VESTA^37^ software.

### 2.4 FE-SEM

By combining FE-SEM imaging and EDX spectroscopy, a comprehensive understanding of the morphological changes and chemical composition variations induced by the shock processing in the sample can be obtained. The imaging and EDX spectroscopy of both pre and post-shocked samples was carried out using a JEOL JSM-7900F Schottky Field Emission FE-SEM. A conductive coating of Pt was applied to all the samples for 1.5 minutes to minimize electron-induced charging effects during imaging. For imaging, an operating voltage of 5 keV was employed, and for the EDX spectroscopy, it was 15 keV.

### 2.5 HR-TEM

HR-TEM analysis of the shocked processed samples was carried out using Titan Themis 300 S/TEM from Thermo Fisher Scientific. The samples were ultra-sonicated in ethyl alcohol for 15 minutes, then drop cast on a quantefoil grid and allowed to dry in a chemically inert atmosphere overnight. Before the imaging analysis started, these drop-cast grids were plasma cleaned using Ar–O_2_ plasma to reduce any changes of further contamination. Throughout the analysis, the HR-TEM was operated at 300 keV. The lattice *d* spacing of the crystalline structures were calculated using ImageJ^38^ software.

## 3. Results

### 3.1 ATR FT-IR spectroscopy

**Fig. 1:**
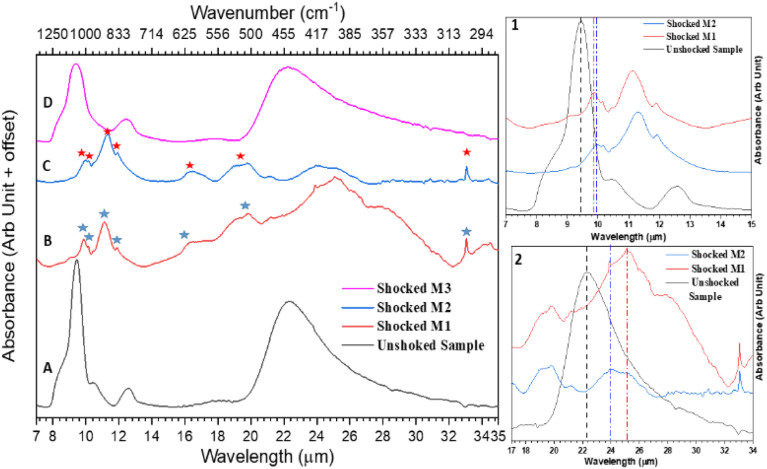
ATR-FTIR spectra of the unshocked sample (A) and shocked samples of M1 (B), M2 (C), and M3 (D). Inset 1 highlights the red shift observed in the Si–O stretching vibration region for the shocked M1 and M2 samples compared to their unshocked counterparts. Inset 2 illustrates the red shift in the O–Si–O bending vibration region for the shocked M1 and M2 samples relative to their unshocked counterparts.

ATR-FTIR spectra of both the shocked and unshocked M1, M2 and M3 samples are shown in Fig. 1. Fig. 1A represents the IR spectra of the unshocked mixtures with peaks around 8.4 μm, 9.4 μm, 10.5 μm, 12.5 μm, 18 μm and around 22.2 μm. The feature around 9.4 μm is associated with the Si–O stretching mode of vibration, the feature around 12.5 μm can be due to the symmetric stretching of the Si–O–Si bond,^15^ and the broad feature around 18 μm and 22.2 μm may be associated to the bending motion of O–Si–O network. The broad peak around 9.4 μm and around 22.2 μm suggests that the mixture contains amorphous SiO_2_.^15^ Since Mg and Fe do not have IR activity, the IR spectra of all three unshocked samples remain similar, and we only showed one spectrum (Fig. 1A) here.

Fig. 1B shows that the IR spectra of the shocked sample of M1 consists of many sharp and prominent features, marked by blue stars. One such feature is around 9.8 μm, accompanied by a shoulder band at 10.2 μm and another broad feature around 9 μm. The other sharp and intense 11.1 μm feature is accompanied by two relatively less intense peaks around 10.4 μm and 11.9 μm. The peaks mentioned above can be assigned to different asymmetric and symmetric stretching motions of Si–O bonds.^4,13^ The peaks around 16.2 μm and 19.7 μm correspond to the asymmetric and symmetric bending motion of the O–Si–O network. Whereas the peak around 23.8 μm could be due to the translation motion of metallic ions in the SiO_4_ matrices. An intense and narrow feature around 33 μm is also observed. This band is assigned to the metal cations translation motion.^4,13^ The drop in the intensity of the entire spectra compared to the unshocked sample spectra suggests shock-induced destruction of Si–O bonds in the sample. A similar kind of spectral feature has been observed in IR spectra of the shocked samples of M2 in Fig. 1C, and the sharp prominent features present there are marked by red stars. The IR spectra of the shocked sample of M3 in Fig. 1D do not show significant variation in their spectral feature. From the IR spectra of the shocked sample of M3, it is difficult to predict the type of solid solution present after shock processing of the mixture of Fe and SiO_2_.

The presence of new sharp spectral features, particularly around 11.1 μm and 33 μm for the shocked M1 sample (Fig. 1B) and 11.2 μm and 33 μm for the shocked M2 sample (Fig. 1C), suggests that the shock-processed samples of M1 and M2 contain crystalline olivine-class silicate dust. In fact, these bands have also been observed as tracers of Mg-rich crystalline silicates in comets such as Hale–Bopp^39^ and around the young stars like HD 100546.^40^ Insets 1 and 2 of Fig. 1 depict the redshift observed in the shocked samples of M1 and M2 compared to their unshocked counterparts. For better visualization of the redshift, three different vertical lines are used in Insets 1 and 2. In the inset 1 the black vertical line at 9.4 μm indicates the stretching vibration of Si–O in the unshocked sample. This stretching mode is found to be redshifted to 9.8 μm, marked by a red vertical line for shocked M1, and to 9.9 μm, marked by a blue vertical line for shocked M2. This observed red shift could be associated with the inclusion of Mg cation into the SiO_4_ network,^15^ which leads to the formation of new solid solutions in both the shocked samples M1 and M2. The Si–O–Si symmetric stretching feature around 12.5 μm in the unshocked sample was found to be missing in the shocked sample IR spectra. This can be associated with the presence of a significant amount of MgO in the shocked sample.^15^ The broadening (inset 2 of Fig. 1) of the O–Si–O bending motion band beyond 20 μm of the shocked samples could be due to coupling of Si–O bending and Mg–O stretching vibrations.^41^ Here we also observed a similar red shift of the O–Si–O bending mode of the shocked M1 and M2 in comparison to their unshocked counterpart. Again for clarity, we have marked the bending mode of unshocked sample by a black vertical line, shocked M1 by a red line and shocked M2 by a blue line. To identify solid solutions in the shocked sample, we compared our IR spectra with reference spectra of crystalline forsterite (Mg_2_SiO_4_), enstatite (MgSiO_3_), and crystalline Mg-rich olivine (Mg_1.8_ Fe_0.2_SiO_4_) from Jäger *et al.*^13^ This comparison indicates that our shocked sample M1 comprises crystalline forsterite and MgO, while shocked sample M2 comprises Mg-rich olivine and MgO. For detailed information about the peaks in the shocked samples and their assignments, refer to Table S1† for shocked M1 and Table S2† for Shocked M2.

### 3.2. XRD Analysis

#### 3.2.1 Sample M1

**Fig. 2:**
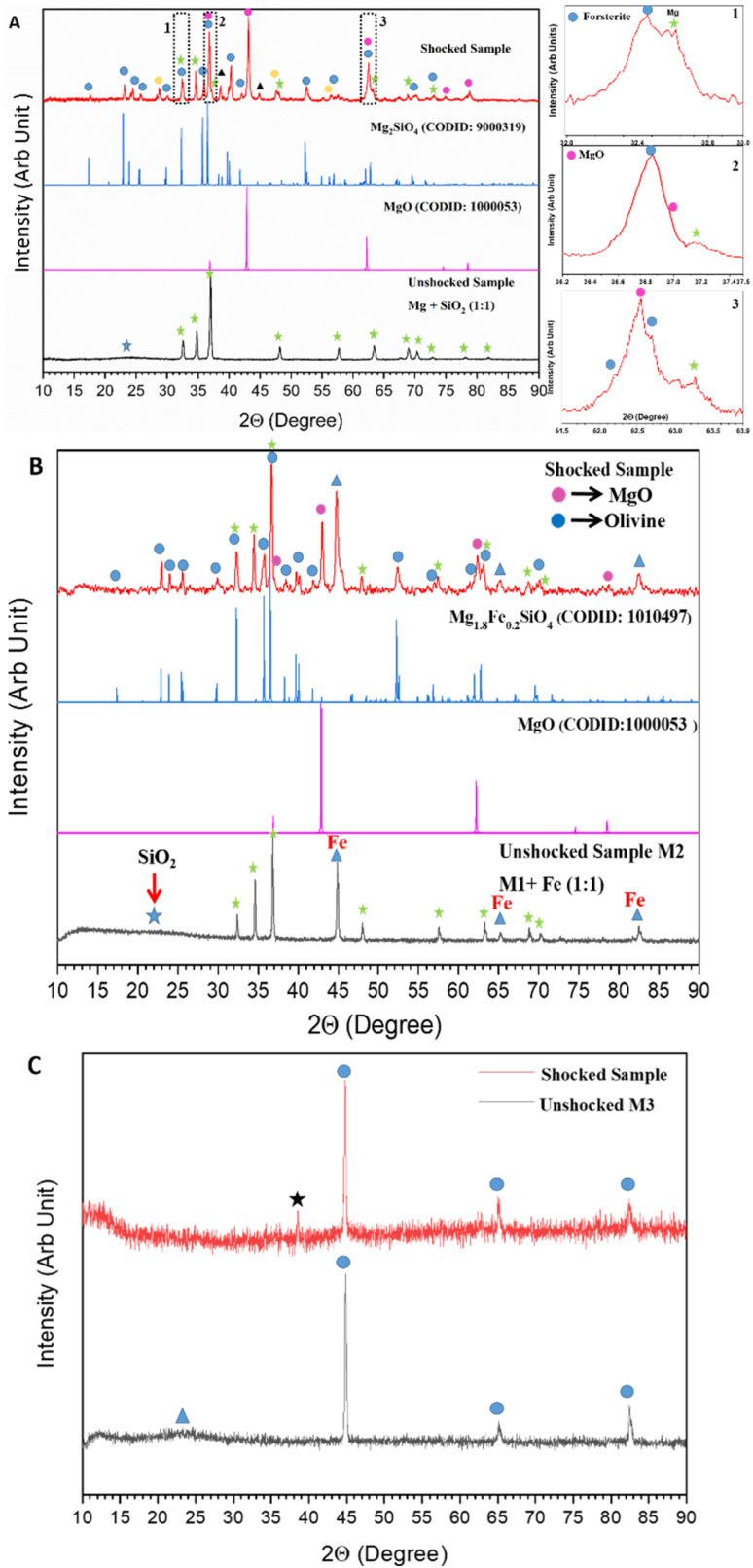
XRD patterns of the shocked and unshocked samples for M1 (A), M2 (B), and M3 (C). Insets 1–3 in panel (A) present zoomed views of the regions within the rectangular boxes in the XRD pattern of the shocked M1 sample.

We performed XRD analysis on both shocked and unshocked samples to identify crystalline phases in the shocked samples. The patterns were normalized to the same scale and offset for visual clarity. Using Bragg's equation, we calculated the interplanar spacing (*d*) of the XRD peaks. Fig. 2A shows the XRD patterns of shocked and unshocked M1 samples. The unshocked sample, represented in black, exhibits a broad feature around 24° (marked by a blue star), indicating the presence of amorphous SiO_2_. Additionally, distinct peaks marked by green stars are attributed to Mg (Crystallographic Open Data (COD) ID: 9008506).

The XRD pattern of the shocked sample, coloured in red in Fig. 2A, has many new peaks compared to its unshocked counterpart, suggesting shock-induced formation of new crystalline phases. To identify these new peaks, we compared our shocked sample XRD pattern with the standard XRD pattern of forsterite (CODID: 900319), available in the COD base. Details of Millar indices and the *d* spacing values of forsterite from both our shocked sample and from CODID: 900319 are shown in Table S3.† It is well known that forsterite belongs to an orthorhombic crystal system with lattice parameters *a* = 4.75 Å, *b* = 10.19 Å, *c* = 5.98 Å, and *α* = *β* = *γ*= 90° ^42^ but because of its large unit cell, there can be many possible planes of reflections, which makes indexing difficult for the high 2*θ* values,^43^ especially after 65°. Besides forsterite, MgO is another crystalline mineral observed in the shocked sample. The new peaks in the shocked sample marked by a magenta colour circle indicate the presence of MgO (CODID: 100053). We also carried out the indexing of MgO's observed peaks by comparing it with its standard XRD pattern, as shown in Fig. 2A. This comparison reveals that MgO present in our sample has a cubic unit cell with *a* = *b* = *c* = 4.21 Å, *α* = *β* = *γ*= 90° and belongs to *Fm*-3*m* space group. Details of the Millar indices and corresponding *d* spacing of MgO present in our sample can be found in ESI Table S3.† Peaks marked by yellow circles at 28.86°, 47.59°, and 56.40° in the shocked sample are assigned to Si (CODID: 1526655), which suggests shock-induced bond destruction of SiO_2_. One can also find the Millar indices and *d* spacing of Si in the Table S3.† The peaks at 38.6° and 44.87°, marked by a black triangle, show Al's presence in the shocked sample, which came from the Al diaphragm used in HISTA. The presence of Mg can also be seen in the shocked sample, and green stars mark the peaks corresponding to Mg. The XRD pattern of the shocked sample shown in Fig. 2A, reveals several peaks, specifically those at approximately 32.5°, 36.8°, and 62.5°, that are marked as common to different crystalline phases. These peaks are labelled as 1, 2, and 3, are further examined in the inset plots on the left-hand side of Fig. 2A. These zoomed-in views clearly reveal that these shaded regions are not single peaks but consist of multiple components on both side of the central peak. This observation suggests that the detected peaks are likely blended reflection features arising from the coexistence of several crystalline phases in the sample. XRD of the shocked processed sample showed the presence of Mg-rich end members of olivine, that is forsterite, metal oxide MgO, Si, and Mg. Amongst these crystalline phases, signatures of forsterite and MgO were observed using IR spectroscopy, and XRD analysis added more values to that information. The shocked sample does not show phases like Mg_2_Si, or the simplest Mg-rich pyroxene class dust enstatite (MgSiO_3_).

#### 3.2.2 Sample M2

The XRD pattern of the both shocked and unshocked sample of M2 can be found in Fig. 2B. The black coloured pattern at the bottom of the Fig. 2B corresponds to the unshocked mixture, showing the presence of SiO_2_ (blue star), Mg (green star), and iron at 45°, 65°, and 82.5° (blue tringle).

At the top of Fig. 2B, the red colour pattern corresponds to the shocked sample and it contains many new peaks compared to the unshocked sample, which suggests the formation of new crystalline phases due to shock processing. Comparison with the standard data available in the COD base suggests that the shocked sample of M2 consists of Mg-rich and iron-poor olivine termed Fe_0.2_Mg_1.8_SiO_4_ (CODID-1010497) corresponding to the peaks labelled by a blue circle, and MgO (CODID: 100053) corresponding to the peaks marked by a magenta circle. For visual clarity, the standard XRD pattern of olivine is plotted in blue (just below the shocked sample's pattern) and the standard XRD pattern of MgO plotted in magenta (just above the unshocked sample's pattern). The peaks labelled with green stars corresponds to Mg and the peaks marked with a blue triangle shows the presence of iron. Details of the Millar indices and corresponding *d* spacing of olivine, MgO present in shocked M2 can be found in the Table S4.† The XRD analysis of the shocked and unshocked samples are consistent with the IR analysis discussed above. Mineral phases such as pyroxene, iron oxides have not been observed.

#### 3.2.3 Sample M3

The XRD pattern of both the pre- and post-shocked mixture of M3 (Fe and SiO_2_ (1 : 1)) is shown in Fig. 2C. As the stoichiometric composition of the mixtures suggests, we see the presence of amorphous SiO_2_, marked by a blue triangle, whereas the peaks marked by the blue circle are assigned to Fe.

At the top of Fig. 2C, the XRD pattern of the shocked sample (shown in red) does not display any change in the sample's chemical composition. The diffractograms showed all three intense peaks of Fe, similar to those observed in unshocked samples. The new peak around 38.6°, (marked by a black star) is assigned to Al, arising from the rupture of the diaphragm. No signatures of iron-rich olivine or pyroxene class of dust are observed. These observations are also consistent with the IR spectroscopic result of the same sample.

### 3.3 FE-SEM imaging

**Fig. 3:**
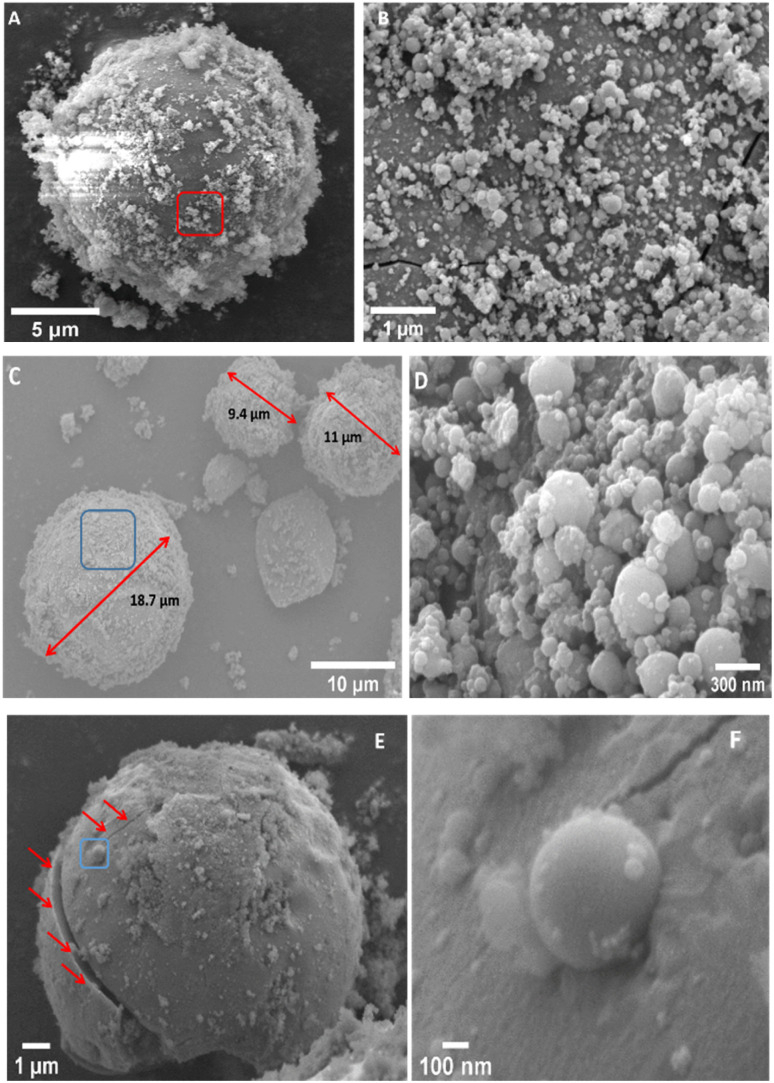
FE SEM images of the shocked samples of M1 (A and B), M2 (C and D) and M3 (E and F).

The morphological studies of the shocked and unshocked samples have been carried out using FE-SEM imaging and EDX analysis techniques. The FE-SEM images of the unshocked samples of M1 can be found in ESI Fig. S3A and B,† which shows the presence of porous grains with different shapes and sizes. A clear change in the morphology of the shocked sample of M1 can be seen in Fig. 3A and B. Fig. 3A shows a compact spherical grain with a diameter of about 15 μm on top of which many smaller grains can be observed. Fig. 3B shows the zoomed view of the region marked by a red rectangle in Fig. 3A, which shows the tiny spherical particles with size ranges from sub-microns to nm. EDX analysis (Fig. S3C†) of these grains suggests that they are an amalgamation of Mg and Si-rich oxides.

As in the case of M1, the morphology of the unshocked mixture of M2 are almost similar; porous grains with different shapes and size can be seen in ESI Fig. S4A and B.† FE-SEM images of the shocked sample of M2 are shown in Fig. 3C and D. Compared to its unshocked counterpart, an apparent change in the morphology of the shocked sample can be observed. Fig. 3C shows spherical dust particles with sizes around 9.4 μm, 12.3 μm, and 18.7 μm. Fig. 3D is a close-in view of the region marked by a blue box in Fig. 3C, which contains smaller spherical aggregates with a typical size range of 300 nm to 30 nm. Point EDX analysis (Fig. S4C†) on one such spherical grain indicates that these grains are made of Mg and Si rich oxides with very little inclusions of Fe.

Fig. S5A and B† shows FE-SEM images of the unshocked mixture of sample M3, which appear similar to those of M1 and M2. Fig. 3E highlights a shock-produced spherical particle approximately 12 μm in diameter, with smaller dust aggregates visible on its surface. Red arrows in Fig. 3E point to a crack located near the equatorial region of the spherical grain. Fig. (3F) reveals smaller spherical grains embedded within a larger spherical grain shown in Fig. 3E, as indicated by a blue box. EDX spectra of the spherical condensate (Fig. S5C†) suggest that these structures consist of iron-rich oxides and silicon.

### 3.4 HR-TEM imaging

#### 3.4.1 Sample M1

**Fig. 4:**
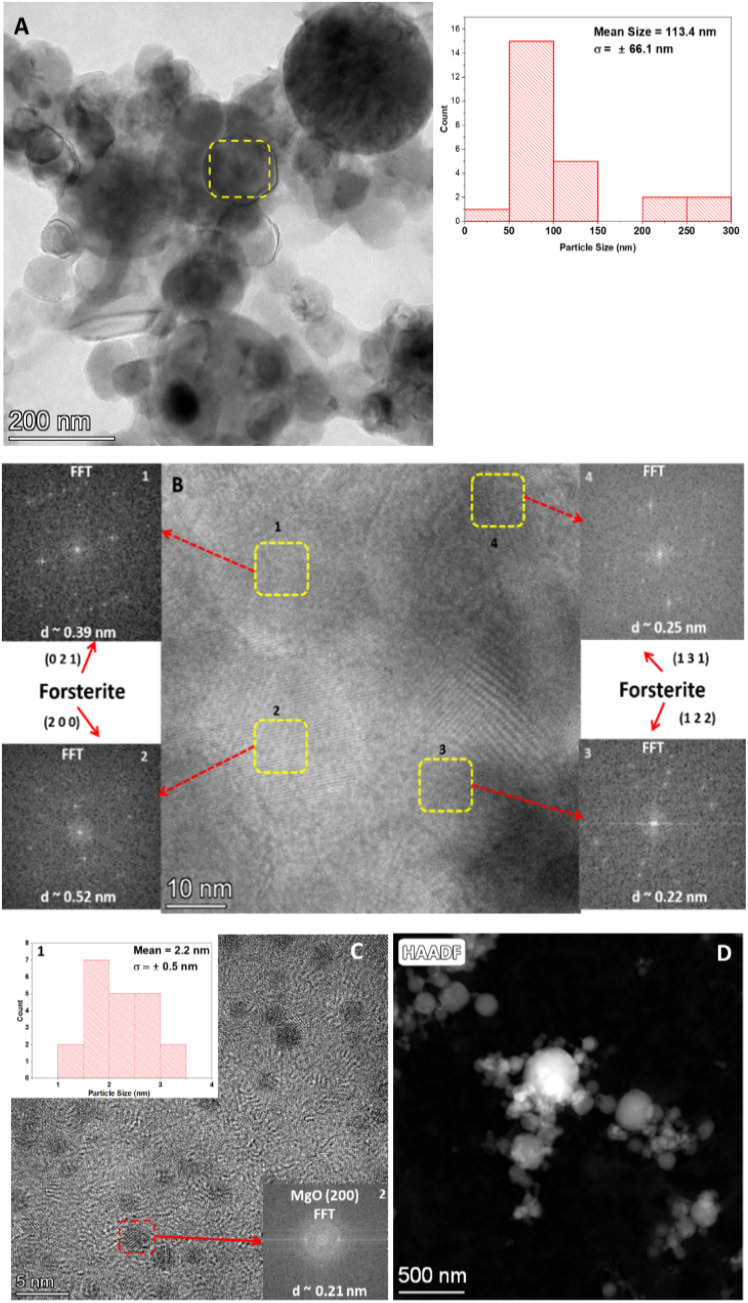
HR-TEM images of the shocked M1 samples: (A) large spherical grains, (B) polycrystalline forsterite, (C) MgO quantum dots, and (D) HAADF image of the distinctive large grains.

HR-TEM images of the shock-processed sample of M1 are depicted in Fig. 4A and D. Fig. 4A is a relatively low magnification image with a scale of up to 200 nm showing the shock-produced spherical dust particles. The inset of this image shows the particle size distribution of the spherical dust particle with with mean particle size around 113.4 nm and a standard deviation (*σ*) of ± 66.1 nm. Fig. 4B shows a zoomed view of the region marked by the yellow rectangle in Fig. 4A. This region consists of many crystalline domains with different orientations. These domains are labelled by 1,2,3,4 and the corresponding selected area electron diffraction patterns can be found in the insets, indicated by a red arrow. The domain 1, located at the top left of Fig. 4B exhibits a lattice spacing of approximately 0.39 nm, which corresponds to the (0 2 1) plane of forsterite (COD ID: 9000319). The domain 2, at the bottom left has a *d* spacing of ∼0.52 nm corresponding to the (2 0 0) plane of forsterite (CODID: 9000319). The domain 3, at the bottom right of Fig. 4B has a *d* spacing of ∼0.22 nm, and corresponds to the (1 2 2) plane of forsterite (CODID: 9000319). The domain 4, at the right top has a *d* spacing ∼0.25 nm, showing the (1 3 1) plane of forsterite (CODID: 9000319). Fig. 4C shows the presence of many nanometer-sized small crystalline domains. Inset 1 of Fig. 4C shows the particle size distribution of these small domains of approximately 2.2 nm and a standard deviation (*σ*) of ±0.5 nm. The interplanar *d*-spacing (0.21 nm) measurement (inset 2) of these small domains indicates that they are made of MgO (2 0 0) crystal plane. Furthermore, their nanometer-sized dimensions suggest they can be classified as MgO quantum dots (QD). Fig. 4D is the High-Angle Annular Dark-Field (HAADF) image of the shock-produced spherical dust particles. The HAADF imaging technique exhibits sensitivity to the atomic number (*Z*) of the elements within the sample. In Fig. 4D the structures that appear bright are composed of elements with a higher *Z* value, specifically magnesium (Mg) and Si. The elemental mapping of these structures can be found in Fig. S6A–C,† whereas the additional MgO quantum dots observed in the shocked M1 sample are shown in Fig. S6D.†

#### 3.4.2 Sample M2

**Fig. 5:**
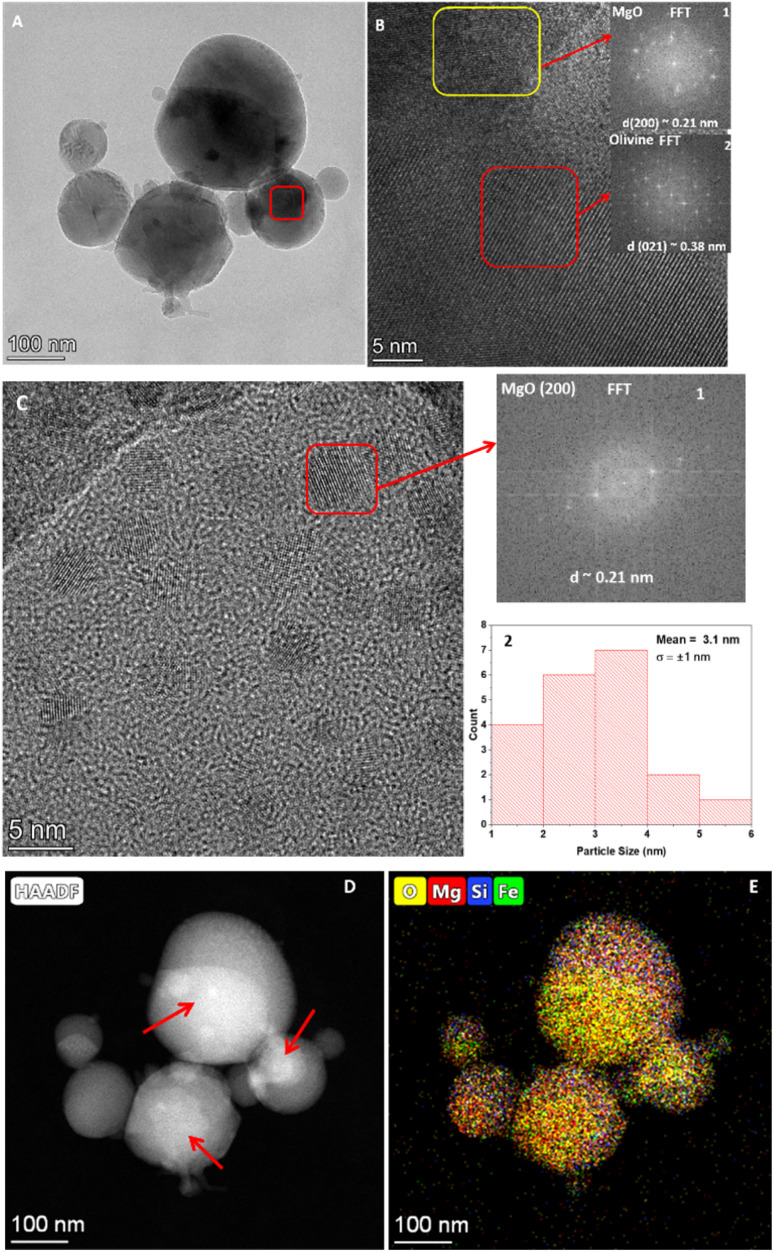
HR-TEM images of the shocked M2 sample: (A) large spherical grains; (B) presence of MgO and olivine within the area marked by a red square in (A); (C) MgO quantum dots along with their particle size distribution; (D) HAADF image of the spherical grains shown in (A); and (E) elemental mapping of the grains depicted in (A).

HR-TEM images of the shocked processed samples of M2 with different magnifications are shown in Fig. 5A–E. Fig. 5A showed the presence of spherical dust grains with a maximum particle size of around 280 nm and a minimum particle size of approximately 35 nm. The inset of Fig. 5A shows the particle size distribution of these spherical dust particles. Fig. 5B shows the high magnification image of the area marked by a red rectangle in Fig. 5A. This area consists of two distinct crystalline phases marked by a yellow rectangle at the top and a red rectangle at the low right corner. Inset 1 of Fig. 5B shows the reciprocal lattice plane orientation of the area marked by a yellow rectangle, and the interplanar *d* spacing of this area is ∼0.21 nm, which corresponds to the (2 0 0) plane of MgO (CODID-1000053). Inset 2 of Fig. 5C indicates the reciprocal lattice orientation of the area marked by a red rectangle. Its interplanar *d* spacing is ∼0.38 nm and corresponds to olivine's (0 2 1) plane (CODID-1010497). Fig. 5C displays the presence of numerous nanometer-sized domains, with their particle size distribution shown in Inset 2. The mean particle size is approximately 3.1 nm, with a standard deviation of ±1 nm. The particle sizes range from 5.1 nm to 1.7 nm. Inset 1 of Fig. 5C describes the reciprocal lattice plane orientation of such a nanometer-size domain marked by a red rectangle. Its interplanar *d* spacing is ∼0.21 nm, which suggests the presence of (2 0 0) plane of MgO (CODID- 1000053), and their nanometer order dimension indicates that they could be MgO (QD). Fig. 5D shows the HAADF image of the same spherical dust grains shown in Fig. 5A. The HAADF image suggests that the core of these spherical grains (indicated by the red arrow) is made of a heavy element; in this case, it is Fe. On top of the iron core, shell-like structures formed, which were made of relatively less heavy elements, in this case, Mg, Si, and O. Fig. 5E shows the elemental mapping of the spherical dust grains in good agreement with the HAADF image in Fig. 5D.† The individual elemental mapping of the above-described spherical particle can be found in Fig. S7A–D.†

#### 3.4.3 Sample M3

**Fig. 6:**
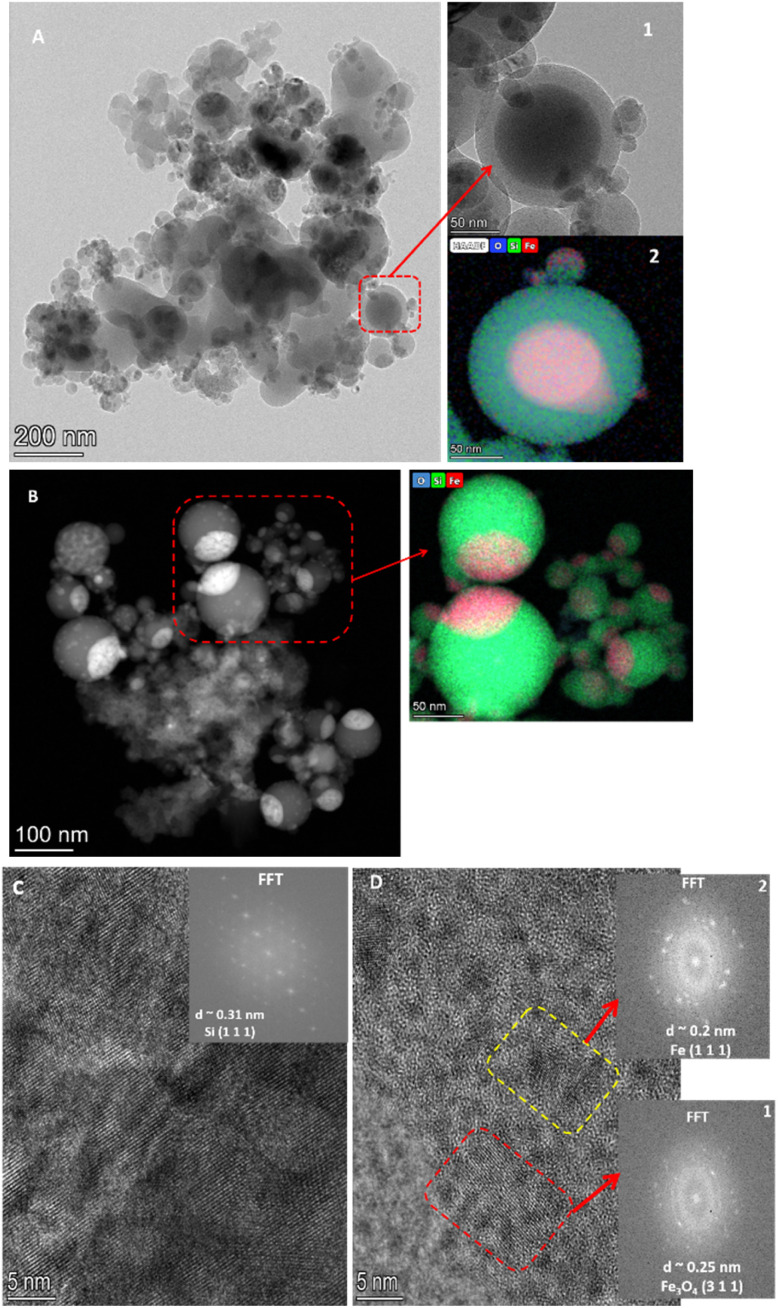
HR-TEM images of the shocked M3 sample. (A) Spherical grains with dense cores and less dense shells; (B) HAADF image highlighting high *Z*-value cores and low *Z*-value shells; (C) nanocrystalline Si; and (D) polycrystalline phases with magnetite (311) in red and Fe (111) in yellow.

The HR-TEM images of the shocked samples of M3 are shown in Fig. 6A–D. Fig. 6A is a relatively low-resolution image up to scale size 200 nm and shows the presence of shock-produced aggregates with a preponderance of spherical particles with particle sizes ranging from 10 to 200 nm. Inset 1 of Fig. 6A shows the zoomed view of the region marked by a red box. The red arrow in the inset shows a core–shell structure with a dense core with a diameter around ∼102 nm and a shell around 140 nm. Inset 2 shows the HAADF images of the core–shell structure along with the elemental mapping. This suggests that the core is made of iron, and the shell is made of Si and O. On top of this core–shell structure, many smaller spherical structures have grown. Observations from Fig. 6A are consistent with the observations of the FE-SEM images of the shocked samples of M3. Fig. 6B shows the HAADF image of spherical dust particles present in the shocked sample of M3. Its inset shows the elemental mapping of the particles within the red box, and it describes that these spherical dust particles are made of Si and have heavy element Fe deposited on the top. With presence of oxygen, these grains are possibly the Fe–Si–O mineral complexes. Fig. 6C is a high-resolution image of the shocked sample with a scale size of up to 5 nm. The inset of this image shows the electron diffraction pattern. The calculated *d* spacing is around 0.31 nm, which can be ascribed to the (1 1 1) plane of Si. Fig. 6D shows the presence of polycrystalline domains. Two of these domains are marked by a red box and by a yellow box, and their corresponding electron diffraction patterns can be found in insets 1 and 2, respectively. The *d* spacing of the structure within the red box is around 0.25 nm, assigned to the (3 1 1) plane of the magnetite (Fe_3_O_4_).^44^ The *d* spacing of the structure within the yellow box is around 0.2 nm, which shows the presence of Fe's (1 1 1) plane. Lin *et al.*^45^ assigned these structures as magnetite QD. However, their formation pathway to synthesize such QD differs from ours. The absence of clear signatures of magnetite, and magnetite quantum dots in the XRD (Fig. 2C) pattern of the shocked sample of M3 can be attributed to the use of a Cu Kα source in the XRD machine. It is known that the energy associated with Cu Kα radiation (∼8 keV) is higher than the K absorption edge of iron. As a result, it can induce X-ray fluorescence from Iron,^46,47^ which may subsequently suppress or mask the peaks corresponding to Fe_3_O_4_ in the XRD pattern.

## 4. Discussions

We have studied shock processing (5.6 M) of interstellar silicate dust analogue precursors M1, M2, and M3 up to a reflected shock temperature of about 7300 K and a reflected shock pressure of about 25 bar for a short period of 2 ms. Under these conditions the sample will experience flash heating with a heating rate of about 10^6^ K s^−1^, with the heating usually persisting for around 2 ms, followed by a rapid quenching with a rate of about 10^5^ K s^−1.^^35,36^ In the shock tube, the processing time is limited, so the reflected shock pressure and temperature play a vital role in the conversion process.^48^ The conversion rate from the precursors to the final product in shock tube operation depends on the properties of the precursor, such as the melting point/sublimation temperature.^48^ Unlike static compression, the shock compression of powder constitutes a non-equilibrium state, which leads to the formation of chemically active species.^49^ These chemically active particles can cause rapid reactions and give rise to new materials. The high pressure and temperature generated by the reflected shock creates a supersaturation state consisting of the precursor fragments. As the high pressure and temperature start to fall, the supersaturated vapour nucleate, and tiny solid grains are deposited on the inner walls of the shock tube and its end flange.^48^

In the M1 shocked sample, the interaction of Mg with the shock-heated Ar (∼7300 K) transforms Mg from its solid phase to the melt phase as suggested in eqn (1).^50^ SiO_2_ also vaporizes at high temperatures and pressures according to eqn (2). Using hydrocode modelling, Melosh^51^ investigated the abundance of different SiO_2_ decomposition products over a wide range of conditions. They found that SiO and O_2_ dominate the gas phase at lower temperatures (with 3500–4500 K at 1 bar), but the composition shifts to predominantly monoatomic Si and O gases as the temperature increases (>4500 K). Integrated results from Melosh^51^ and Toguri and Pidgeon,^52^ and our analysis of shocked M1 samples allowed us to develop a set of chemical equations that may lead to the formation of forsterite and MgO. The possible reactions can be as follows:

1 Mg + Ar → Mg* + Ar

2 SiO_2_ + Ar → SiO + O + Ar

3 Mg + O → MgO

4 2Mg + 4SiO → Mg_2_SiO_4_ + 3Si

The reaction involving the formation of MgO and forsterite (eqn (3) and (4)) is exothermic. The release of heat facilitates the conversion of the reactants into their respective solid phases leading to a non-classical two-stage particle attachment mechanism that can be used to understand the crystallization from high-temperature melt or extreme metamorphic phases.^53^ This process starts with the formation of multi-ion complexes. In the first stage, nanoparticles are formed, which undergo oriented aggregation. During this aggregation process, the building blocks of the crystals may exhibit a common crystallographic orientation or have slight rotations relative to each other. Fig. 4A indicates a scenario where different crystalline domains consist of various phases of forsterite oriented in different directions. In the second stage, the grains undergo coarsening through the attachment of the nano-flakes. In this step, the individual nano-flakes can have varying orientations, and the attachment process helps grow larger grains.

Analysis of the shocked sample of M2 showed new crystalline phases such as polycrystalline olivine (Mg_1.8_Fe_0.2_SiO_4_) and MgO. The olivine showed a significantly lower iron inclusion. However, the HAADF image (Fig. 5E and F) of the core–shell structure in the shocked sample shows that iron provides a finite area for silicate dust growth. During the shock processing, the shock-heated Ar will transfer the heat to the Mg, Fe and SiO_2_. At the shock-heated gas temperature ∼7300 K, most of the Mg and Fe will be in the melt phase^54^ and start to react with the SiO and O, which are formed due to destruction of SiO_2_ according to eqn (2). In these circumstances, the MgO formation pathways can be similar to what we see in the case of M1 *via*eqn (4). MgO will starts condensing below 2500 K but Mg will still be available and again react with the available SiO and Fe, which leads to the formation of olivine *via* the following equation.

5 Mg + SiO + Fe + Ar → [MgFeSiO_4_] + Fe + Ar

here [MgFeSiO_4_] is the solid solution of silicate dust.

In the shocked sample of M3, we observed the presence of crystalline Si, Fe, and magnetite QDs. Iron will be in the melt phase during the shock, heating up to a gas temperature of about 7300 K,^54^ whereas SiO_2_ will dissociate into species like SiO, O, and Si.^51^ The reaction between shock-heated Fe and SiO_2_ can lead to FeO and FeSi forming *via* the following reaction (6), suggested by Dubrovinsky *et al.*^55^

6 3Fe + SiO_2_ → 2FeO + FeSi

FeSi and FeO can further oxidize, leading to the formation of Fe_2_O_3_*via* the following reaction.^56^

7 4FeSi +7O_2_ → 2Fe_2_O_3_ + 4SiO_2_

8 4FeO + O_2_ → 2Fe_2_O_3_

Fe_2_O_3_ can further be oxidized and turned into Fe_3_O_4_.^57^

As dust cools from the high temperature melts, spherical grains are often formed due to the surface tension.^58^ The varying melting points of its constituent elements shape the structure of the spherical grains. In Fig. 6A, insets 1 and 2 reveal that iron (Fe) forms the core of the dust grains, encapsulated by a shell composed of silicon (Si) and oxygen (O). This core–shell structure likely arises from the differing melting points of Fe, Si. Iron has a significantly higher melting point, causing it to condense first, providing a spherical surface upon which Si and O can react and form the surrounding thick shell. These core–shell structures have varying particles sizes and for the case of M1 (Fig. 3A and 4D) it ranges between ∼30 μm to 300 nm. Because of their varying particle size these spherical grains may experience differential drag during the expansion of the shock wave and collide with each other and make larger grains.^59^ In some cases, as suggested by Jacquet and Thompson,^60^ these collisions can cause cracks on the dust grains and lead to the destruction of the grains. Fig. 3B and 3E shows some of the cracks in the shocked samples of M1and M3, whereas ESI Fig. S3D† shows the presence of a fractured spherical grain in the shocked sample of M1.

## 5. Astrophysical significance

We have observed the presence of Mg-rich olivine and MgO signatures in both shocked samples of M1 and M2. According to the theoretical work by Gail and Sedlmayr,^61^ a fraction of MgO is expected to condense along with iron grains during the growth of silicate dust. Therefore, the presence of MgO in both shocked samples of M1 and M2 aligns well with the predictions. Furthermore, observations of crystalline dust in various circumstellar regions have shown a prevalence of Mg-rich compositions rather than iron despite the similar abundance of magnesium and iron in stellar envelopes.^62^ The results from the shock processing of the sample M2 indicate that, starting from a mixture with an equal proportions of Mg and Fe, the formation of Mg-rich crystalline silicate dust, particularly in the olivine class, is the most probable outcome, compared to pyroxene or iron-rich silicate dust. For sample M3, the newly formed phases are crystalline Si and magnetite (Fe_3_O_4_) QD. Magnetite is a permanent magnetic material and ferromagnetic^63^ below 580 °C. Magnetic nanoparticles such as magnetite in the ISM could potentially contribute to anomalous microwave emission^64^ and can contribute to interstellar polarization by aligning themselves towards the galactic magnetic field.^65^ They can also influence the dust growth process as the collisional efficiency of the magnetically aligned grains is different from that of the non-magnetic grains; hence, the rate of dust growth will also be different.^66^ Magnetically aligned dust grains may also form elongated and compact grains compared to non-magnetic grains.^67^

The results of the present work can be used to explain the formation of chondrules in the early solar nebula. Chondrules are mm-size spherical dust grains made of silicates, one of the major constituents of the chondritic meteorites. Their texture and mineral composition suggest that they are formed by short bursts of melting, happening repeatedly in localized areas, with each melting event lasting only minutes to hours.^68^ Amongst the different proposed formation pathways, nebular shock processing of the chondrules precursors, induced by gravitational instabilities or bow shock generated from the supersonically moving planetesimal, shows good agreement with the petrological and geological tests.^68,69^ Such shock waves with a velocity of 5 to 9 km s^−1^ are sufficient to raise the nebular gas temperature to 4000 K and more, which can melt the available chondrule precursors. In the presence of optically thin shocks, these melts quickly turn into supercooled droplets. These supercooled droplets may undergo multiple collisions, leading to the condensation of chondrule behind the shock front.^33,59^ To the best of our knowledge, this study represents the first application of a shock tube to investigate the formation of cosmic minerals behind a shock front. By effectively replicating shock-induced mineral grain formation, our work demonstrates that shock processing (5.6 M, 7300 K, for 2 ms) of chondrule precursors can produce chondrule-like structures, providing experimental validation for theoretical models proposed over the past 50 years.

In the laboratory, the shock processing time scale is limited to a few milliseconds, which is significantly shorter compared to conditions in the early solar nebula. Despite this, our experiments demonstrate good consistency with various models of shock-induced chondrule formation in the ISM described above. For instance, the presence of spherical metallic grains in all three shocked samples indicates that their elemental precursors underwent shock-induced melting followed by rapid cooling at a rate of approximately 10^5^ K s^−1^. FE-SEM images in Fig. 3B and D–F suggest that during this rapid cooling, these grains or molten droplets, exhibiting a wide range of particle sizes, experienced collisions and leading to the formation of larger grains.

IR spectra of comets like Hale–Bopp^39,70^ and Comet NEAT^5^ show the presence of crystalline silicates. Different formation pathways of the crystalline silicates in comets have been proposed, which include: (i) thermal annealing of amorphous pre-existing silicate grains in the presolar nebula where the comets are formed,^71^ (ii) direct incorporation of crystalline silicate grain into the comets from the ISM.^72^ (iii) The shock processing of amorphous silicate dust through high-velocity shock waves in the outer regions (∼10 AU) of the solar nebula has also been proposed to lead the formation of crystalline silicate dust, which may subsequently be incorporated into comets.^31,32^ A shock wave with velocity ∼5 km s^−1^ propagating through a medium with pre-shock density ∼5 × 10^−10^ g cm^−3^ can lead to flash heating of amorphous micron size dust grains up to a temperature of ∼1000 K, and this sharp thermal spike will remain in the grain for ∼2 s.^31,32^ The exact sources of such shock waves in the outermost part of the presolar nebula are still a topic of ongoing discussion. However, a spiral density wave^73^ in a gravitationally unstable disk could form a shock wave with a velocity of ∼10 km s^−1^, which can thermally process amorphous silicate dust and convert it to crystalline silicate dust. Our experiments suggest that shock processing of the cometary silicate dust precursors can lead to the formation of the crystalline olivine and MgO (Fig. 2B) class of dust, which has been reported in different comets.

## 6. Conclusions

We have shown the bottom-up formation of cosmic minerals by subjecting their elemental precursor to intense shock conditions (5.6 M, 7300 K, for 2 ms). Analysis of the shock-processed samples leads to the following observations:

(1) Shock processing of a mixture of Mg and SiO_2_ leads to the formation of forsterite and MgO.

(2) Shock processing of mixture Fe, Mg and SiO_2_ leds to the formation of Mg-rich olivine dust and MgO.

(3) Shock processing of a Fe and SiO_2_ mixture leads to the formation of magnetite at the nm level, suggesting the presence of magnetite QDs.

(4) We have not seen any signatures of the pyroxene class of dust in any of the three sets of experiments.

(5) The results are in good agreement with the hypothesis that shock processing of the mineral dust precursors can lead to the formation of chondrules and crystalline silicate dust known to be present in chondritic meteorites and comets.

(6) The variation in melting points of different elements under intense shock conditions plays a key role in shaping the morphology of the dust grains. During the formation of dust grains, elements with higher melting points condense first and solidify, forming a core. Lower-melting-point materials then condense around this core, creating an outer shell.

From the experimental results, we also propose that mineral QD's play a major role in the interstellar emissions which is yet to be explored.

## Data availability

The data supporting this article can be found https://prlnabh.prl.res.in/prlnabh/s/DmRsCcQdxJB2rsF.

## Author contributions

Conceptualization: Arijit Roy, Surendra V. Singh, R. Ramachandran, B. Sivaraman. Data curation: Arijit Roy, Surendra V. Singh, R. Ramachandran, M. Ambresh, V. Venkatraman, N. J. Mason, B. Sivaraman. Formal analysis: Arijit Roy, Surendra V. Singh, R. Ramachandran, J. K. Meka, M. Ambresh, V. Venkatraman, P. Janardhan, A. Das, H. Hill, N. J. Mason, B. Sivaraman. Funding acquisition: Anil Bhardwaj, P. Janardhan, N. J. Mason. B. Sivaraman. investigation: Arijit Roy, Surendra V. Singh, R. Ramachandran, B. Sivaraman. Methodology: Arijit Roy, Surendra V. Singh, R. Ramachandran, J. K. Meka, M. Ambresh, T. Vijay, V. Venkatraman, P. Janardhan, H. Hill, N. J. Mason, B. Sivaraman. Supervision: B. Sivaraman. Writing – original draft: Arijit Roy, R. Ramachandran, B. Sivaraman. Writing – review & editing: Arijit Roy, Surendra V. Singh, R. Ramachandran, J. K Meka, M. Ambresh, T. Vijay, P. Janardhan, V. Jayaram, V. Venkatraman, A. Das, H. Hill, Anil Bhardwaj, N. J. Mason, B. Sivaraman.

## Conflicts of interest

There are no conflicts to declare.

## Supplementary Material

RA-015-D5RA01088H-s001

RA-015-D5RA01088H-s002
