# Supplementary material for: Instantaneous formation of interstellar minerals and mineral quantum dots
Source: RSC Adv. 2025 Apr 17;15(16):12309–20. doi: 10.1039/d5ra01088h (PMC12004361; doi:10.1039/d5ra01088h)
Supplement: RA-015-D5RA01088H-s001 [file RA-015-D5RA01088H-s001.zip › additional images of shocked samples.pptm]

## Slide 1
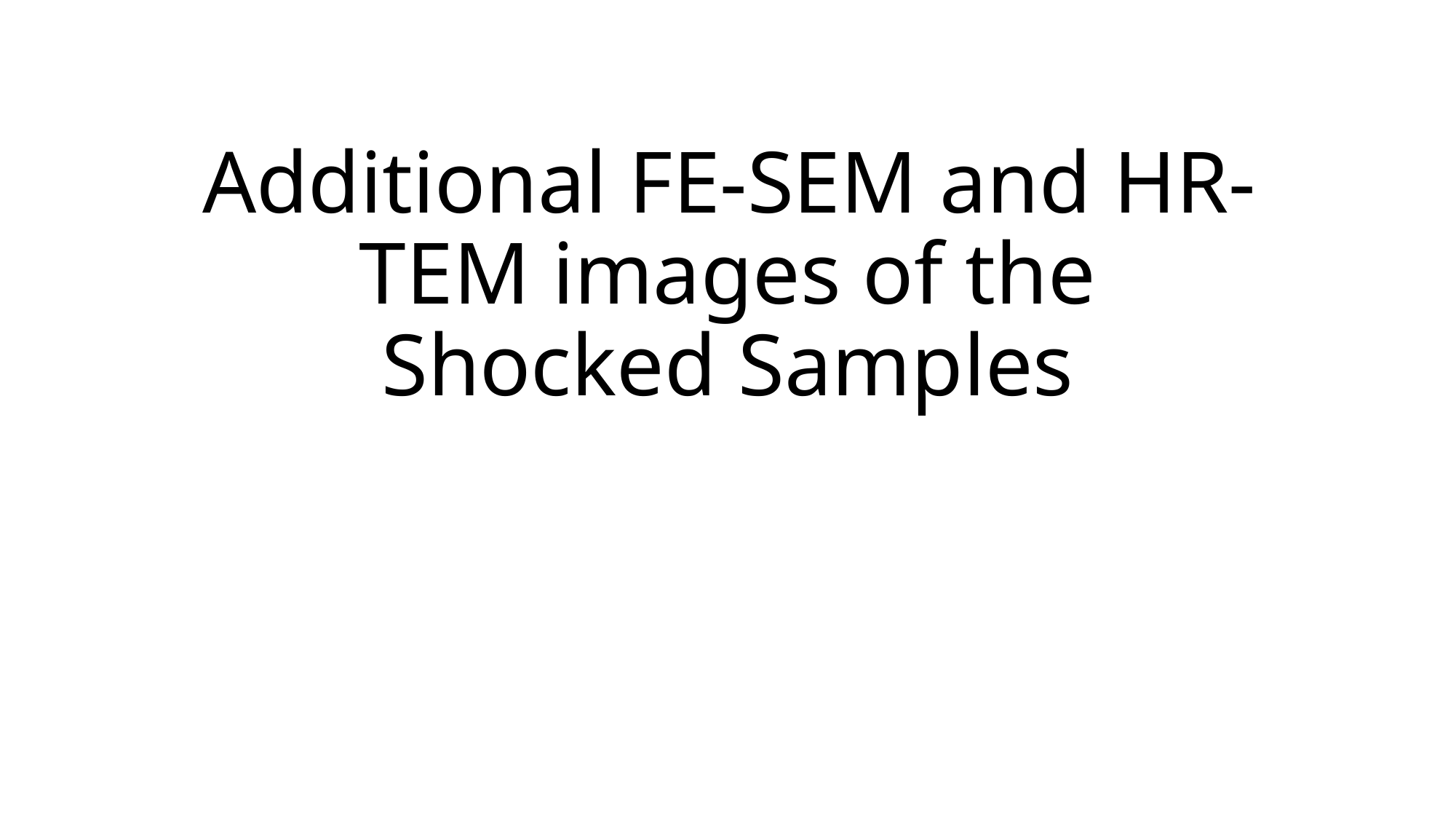

# Additional FE-SEM and HR-TEM images of the Shocked Samples

## Slide 2
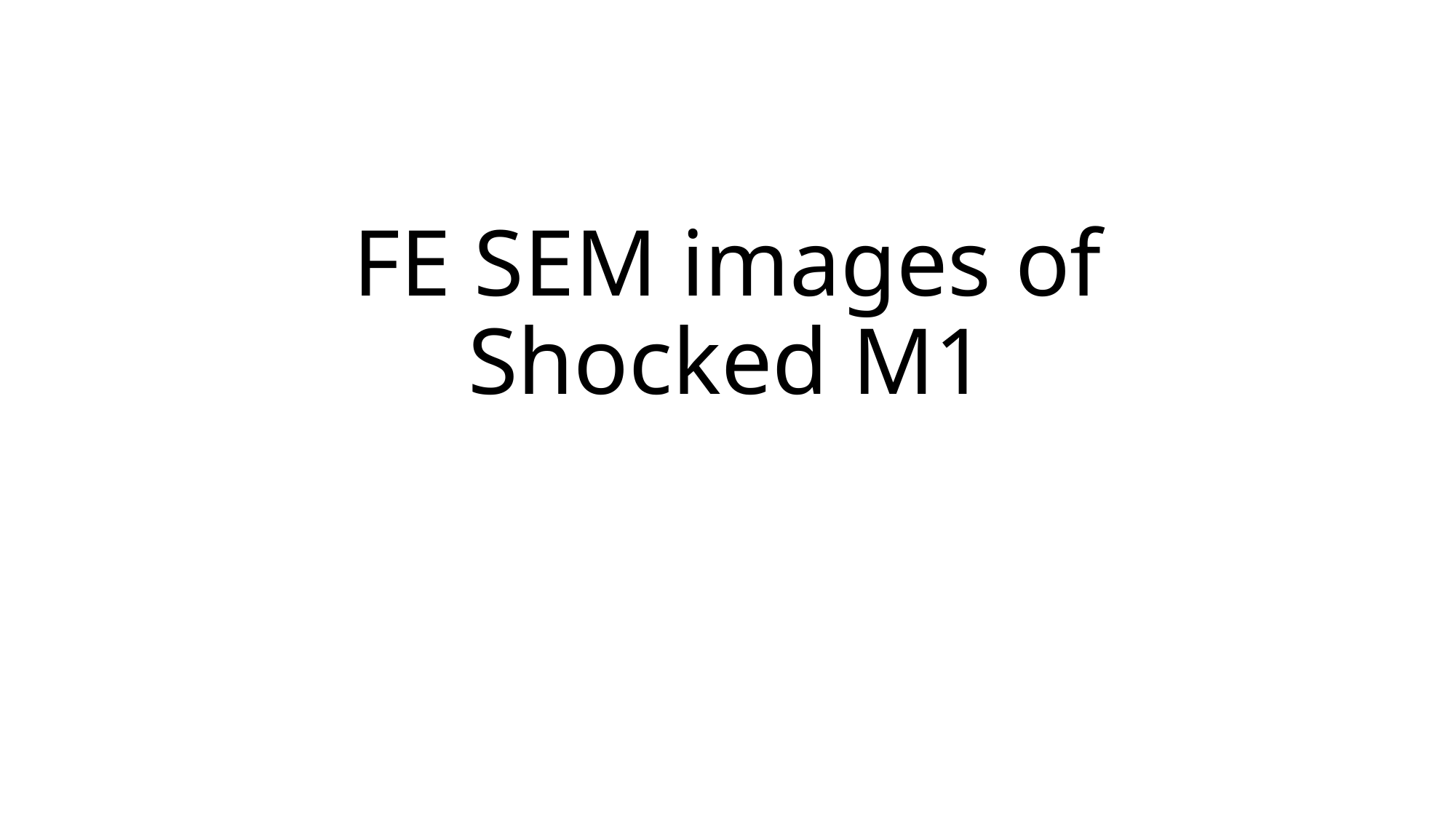

# FE SEM images of Shocked M1

## Slide 3
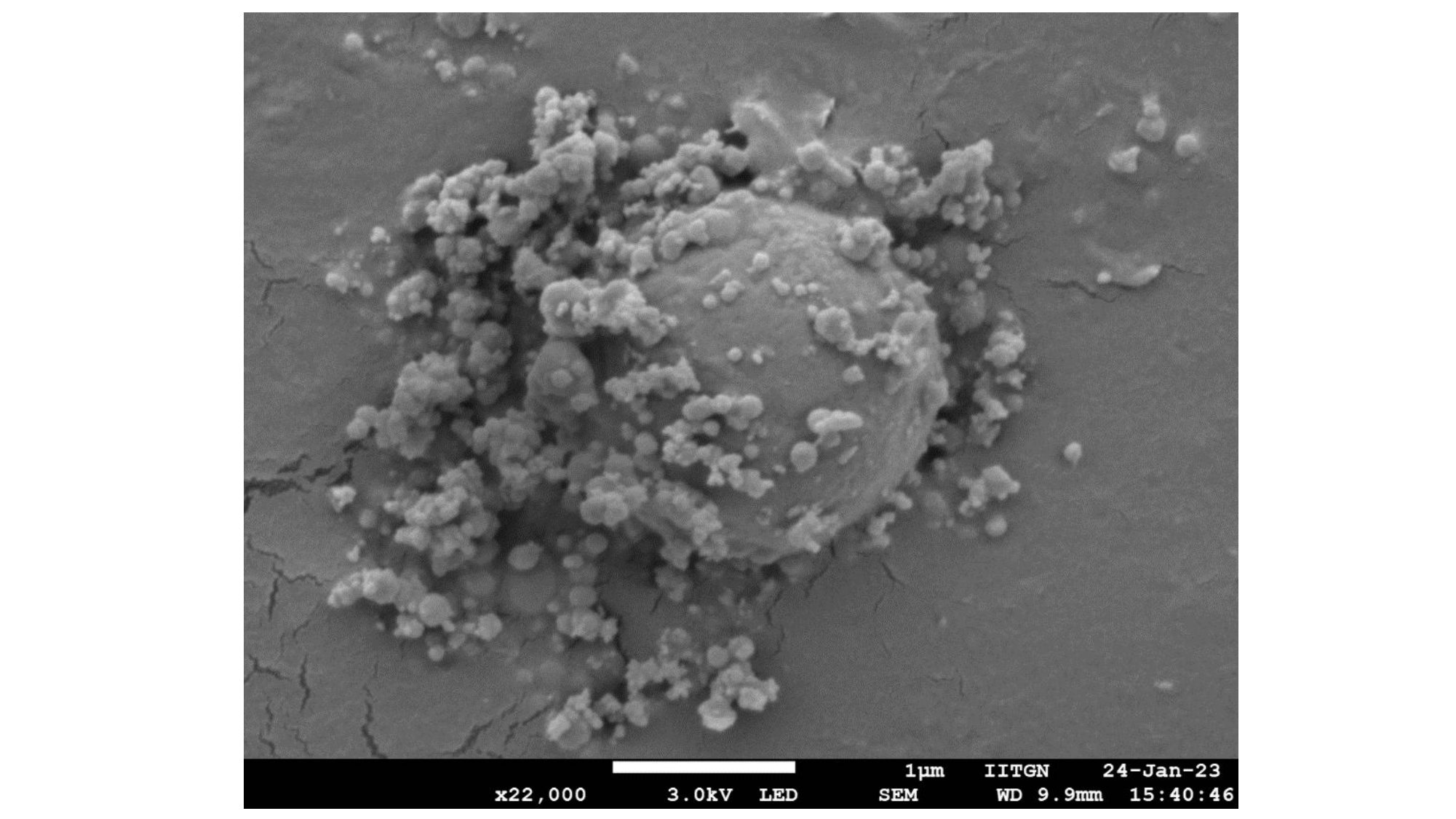

## Slide 4
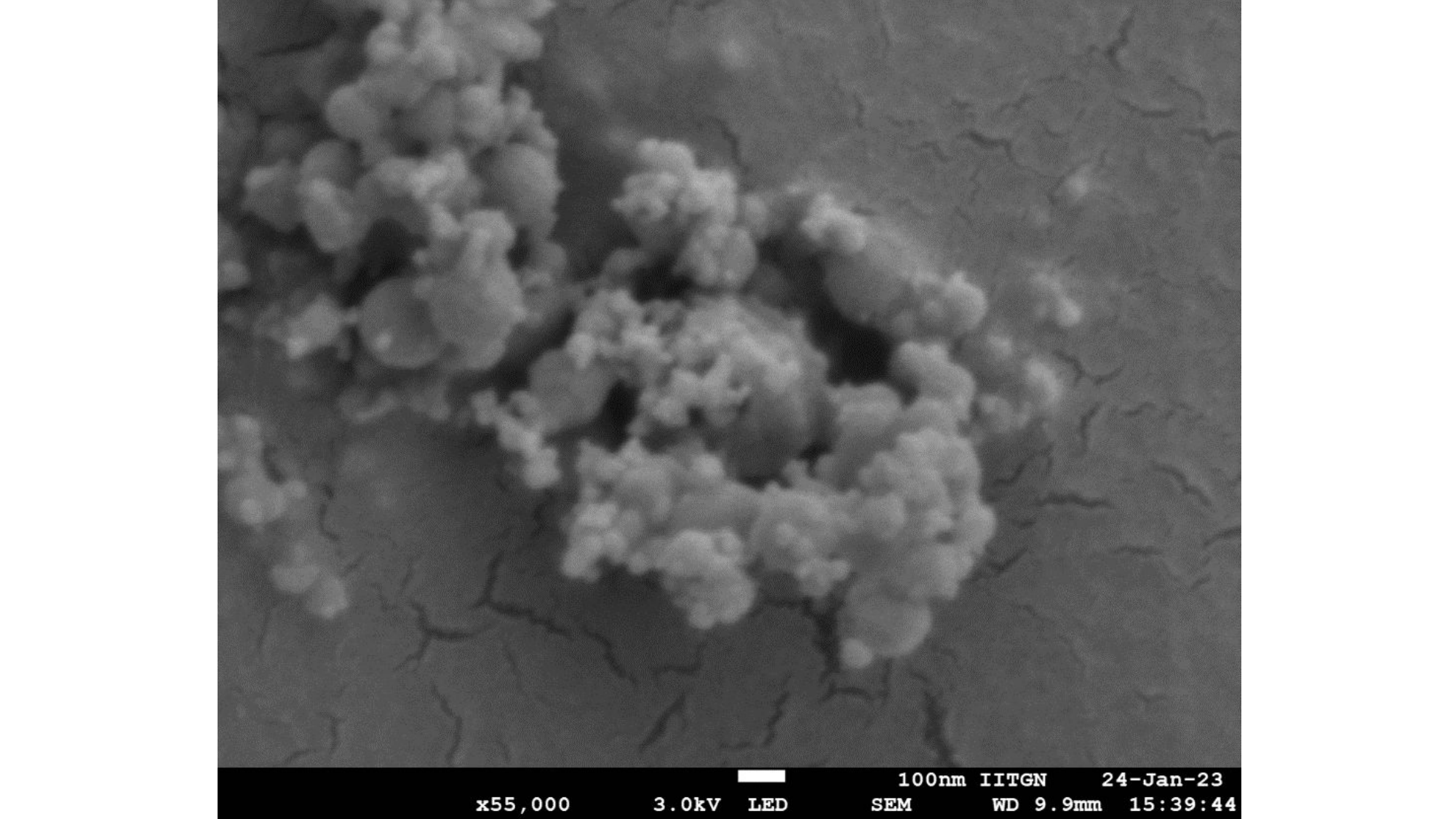

## Slide 5
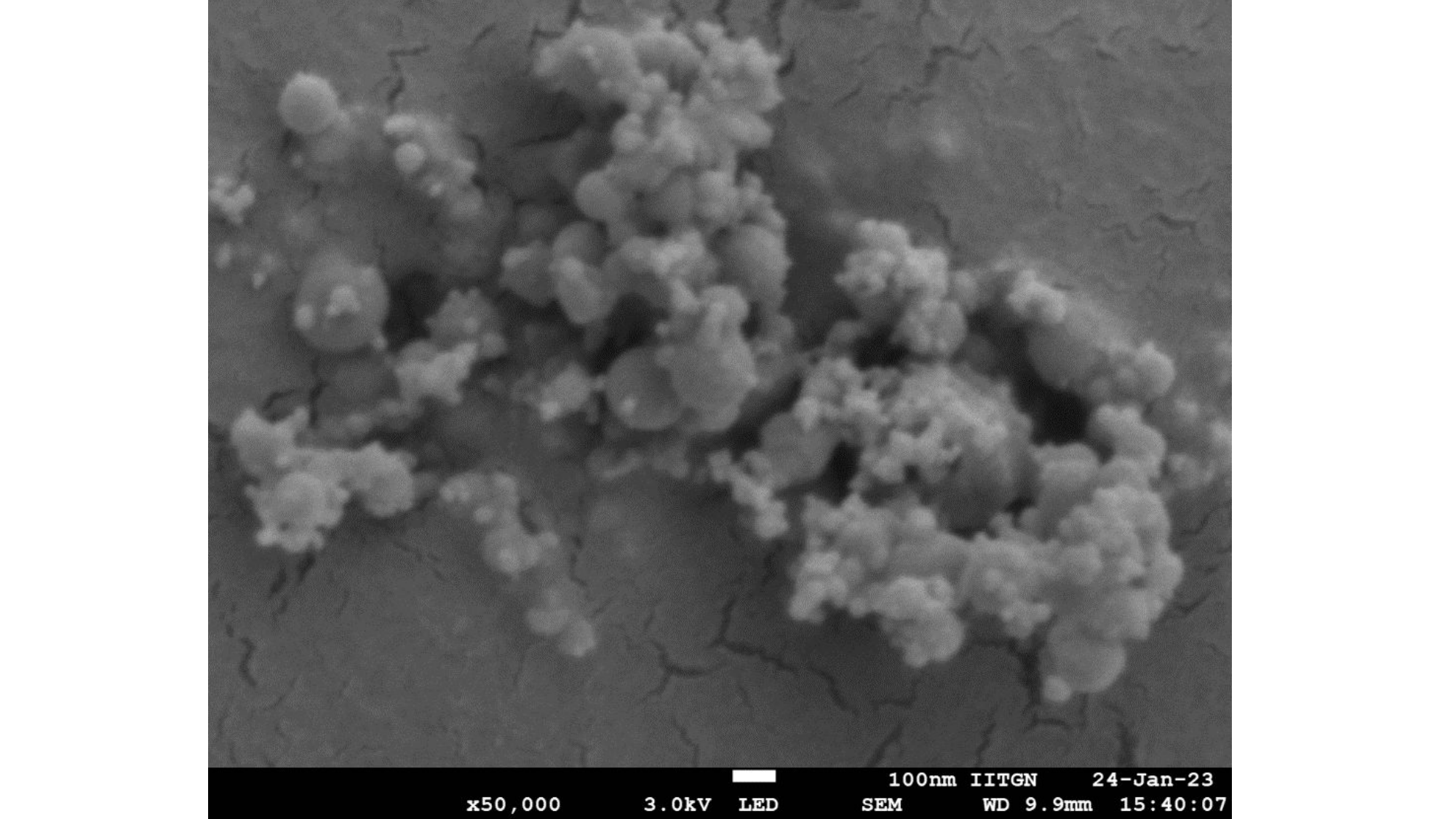

## Slide 6
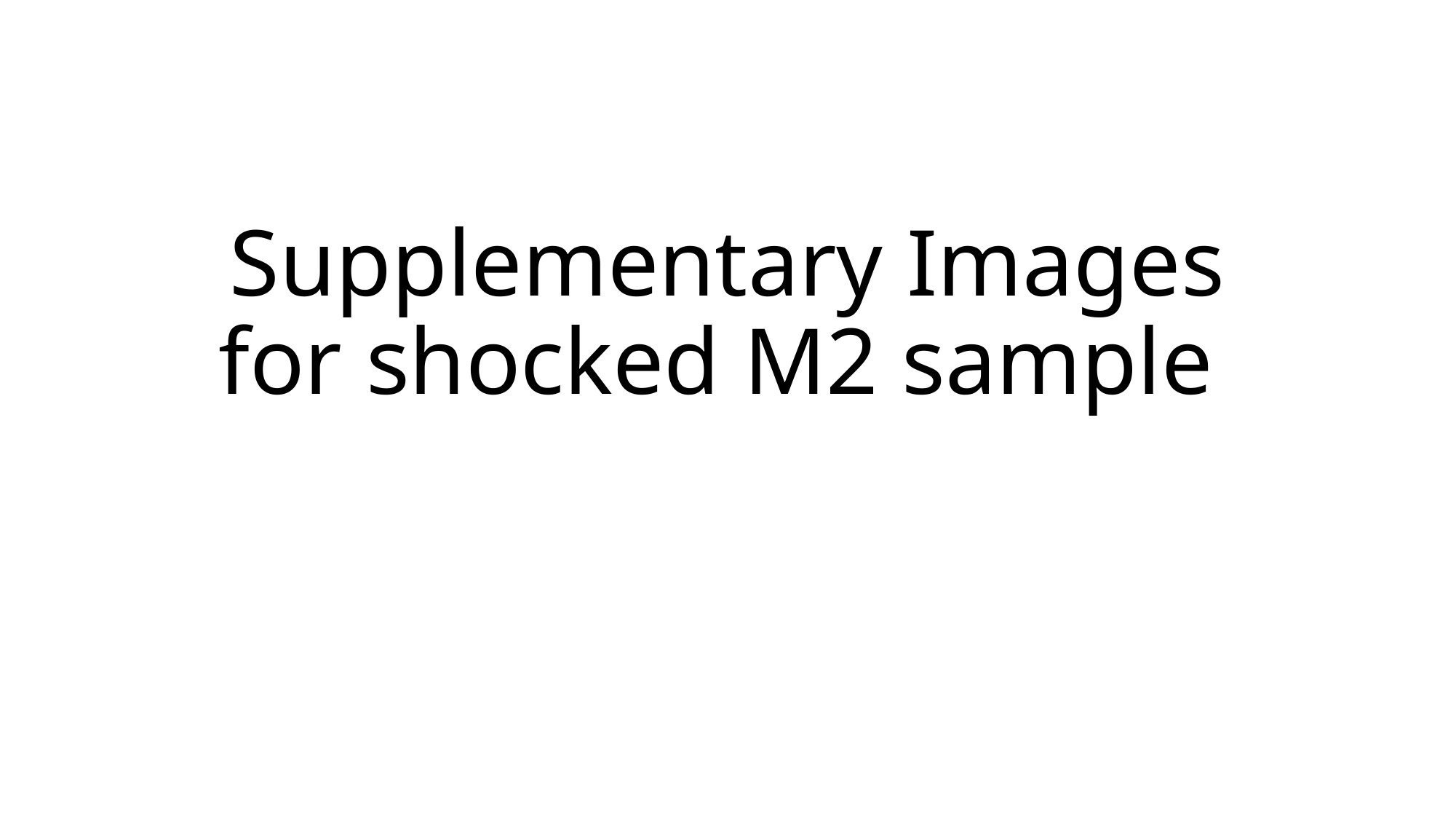

# Supplementary Images for shocked M2 sample

## Slide 7
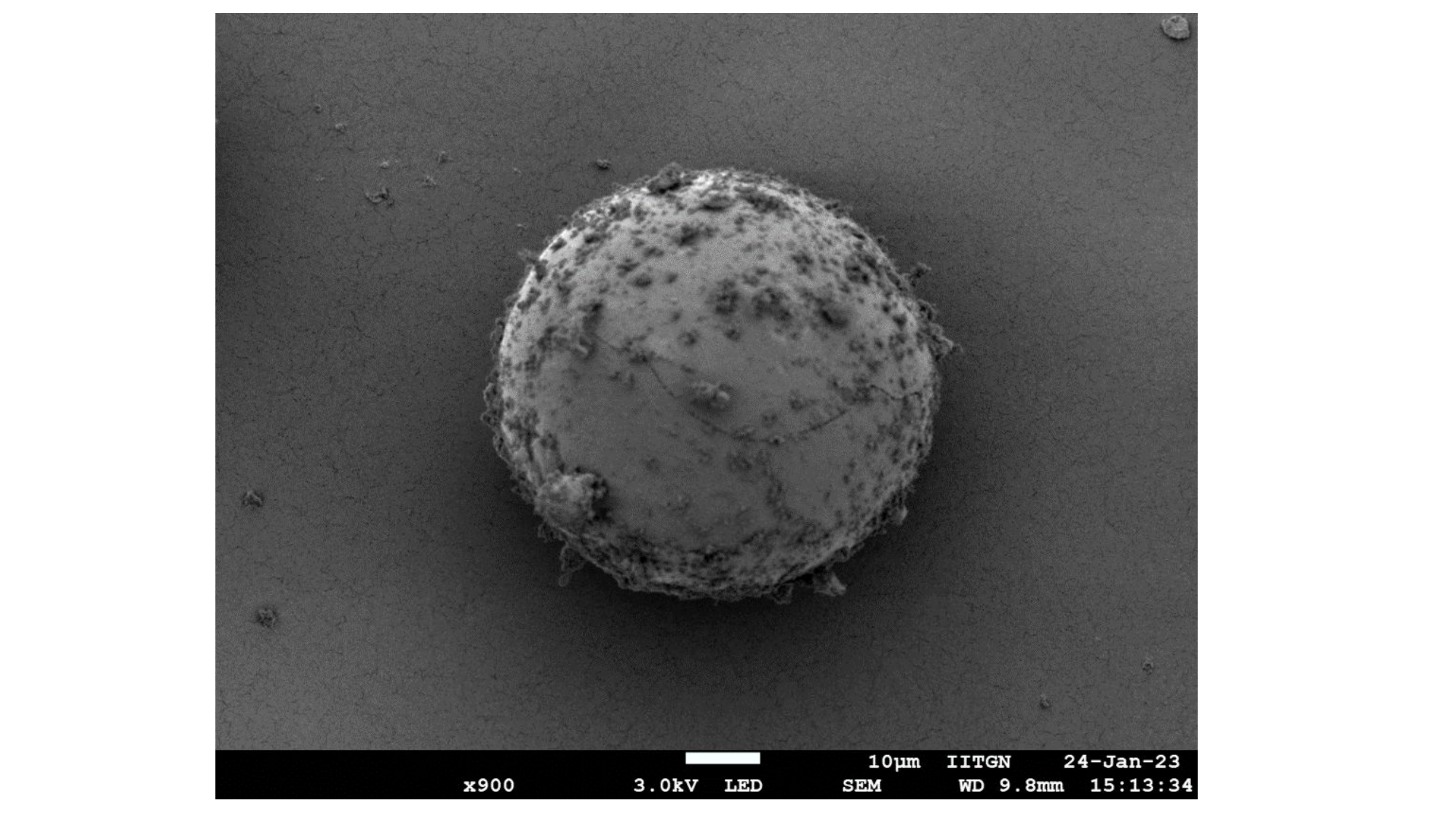

## Slide 8
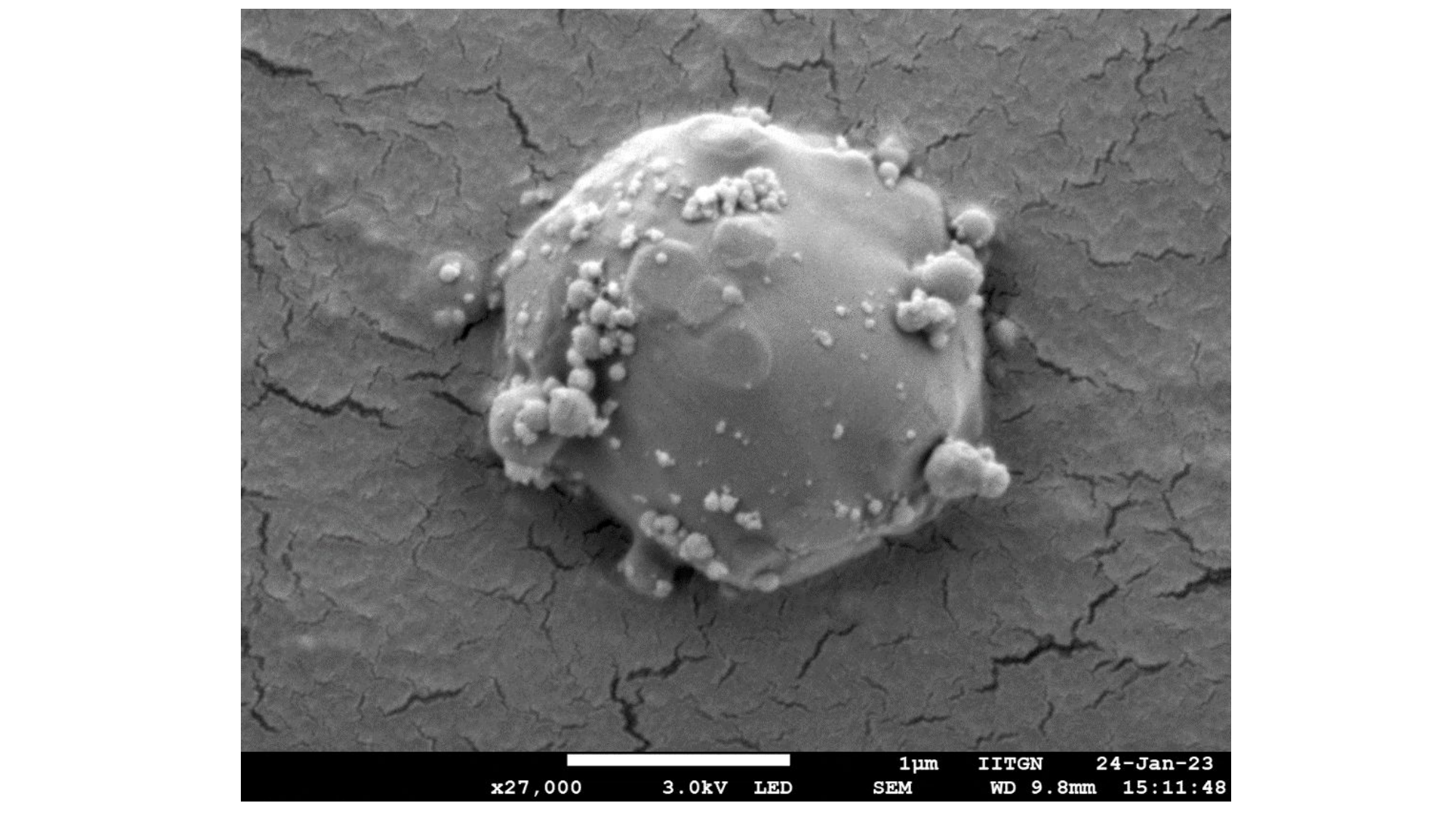

## Slide 9
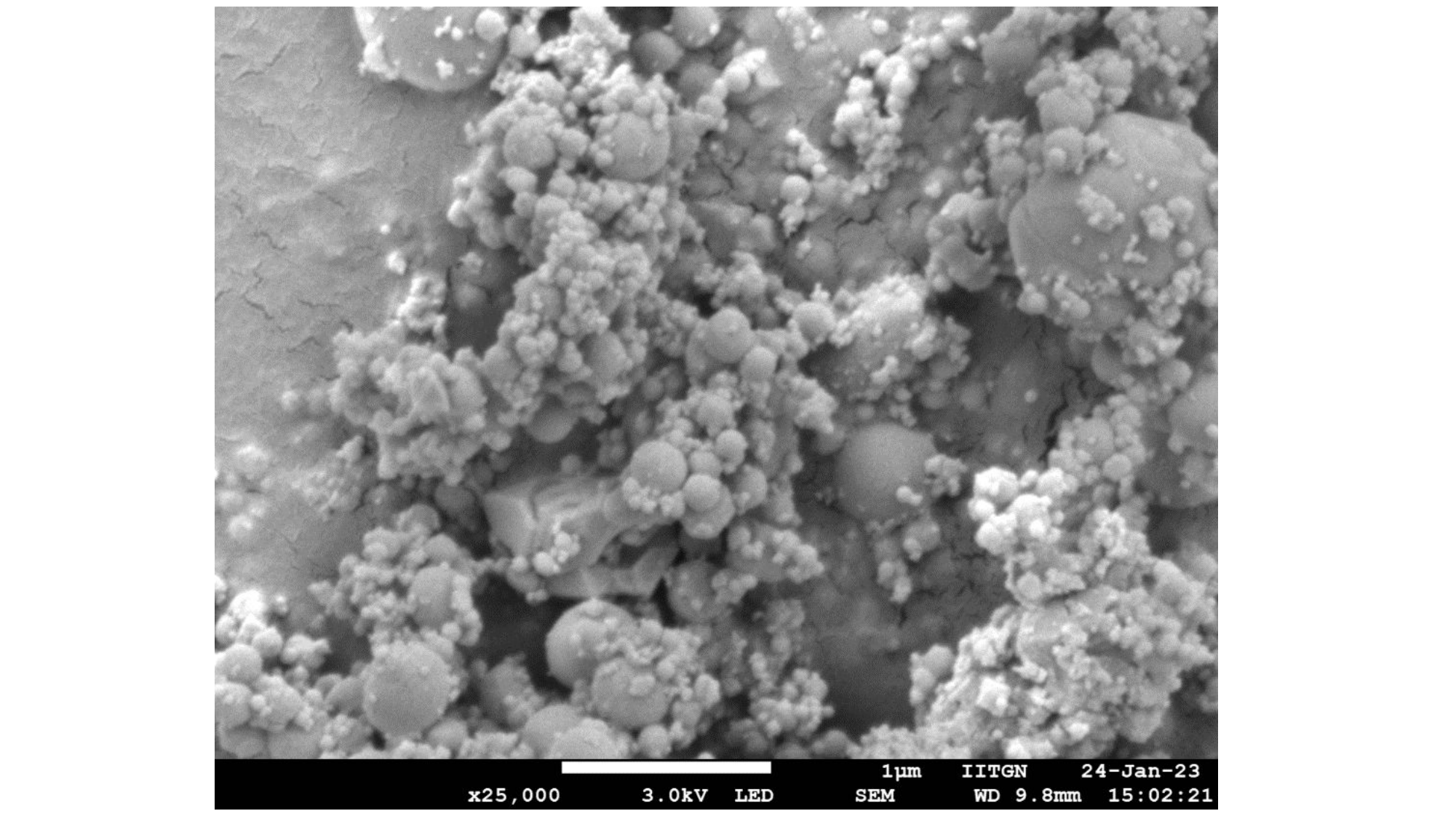

## Slide 10
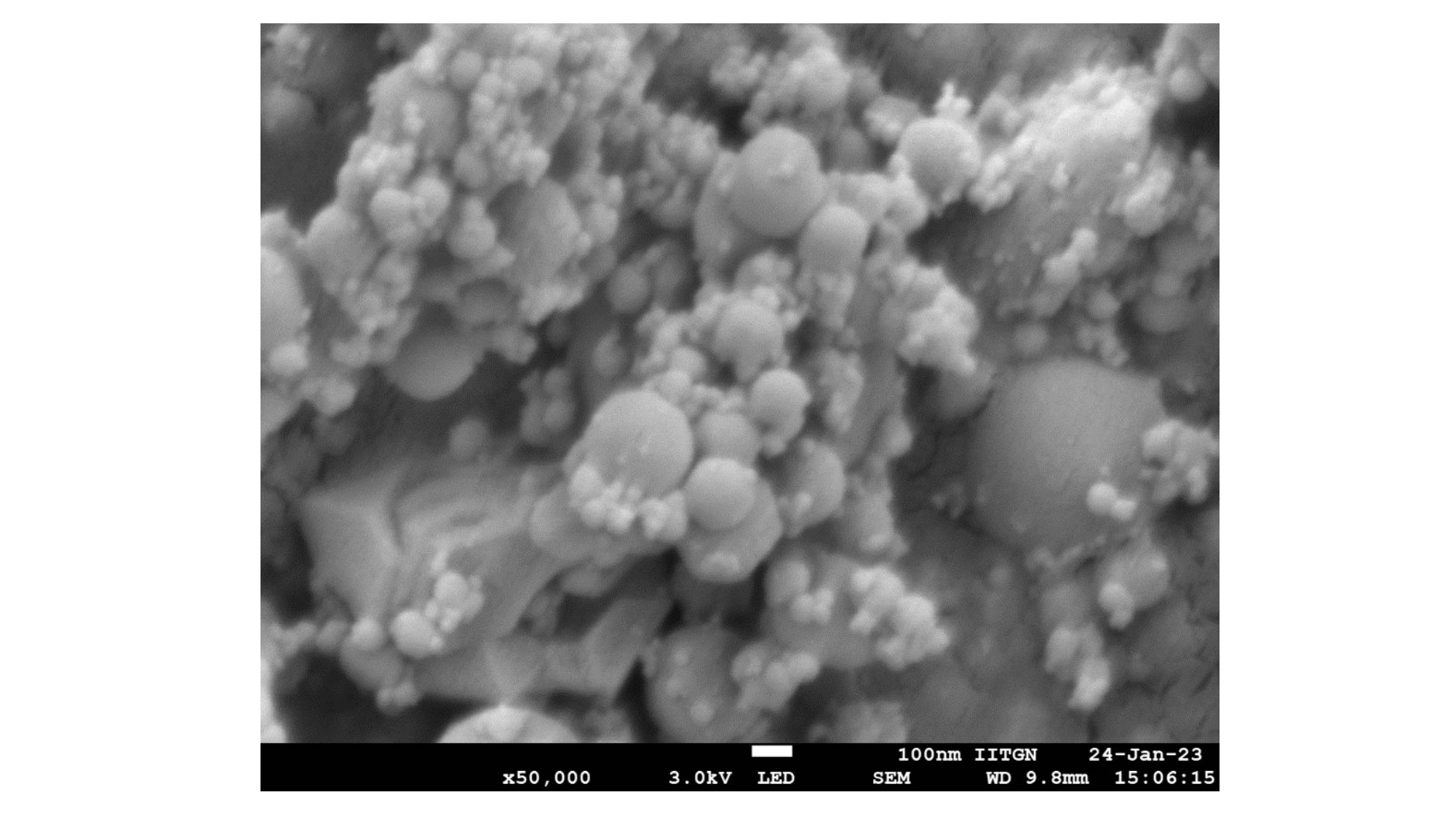

## Slide 11
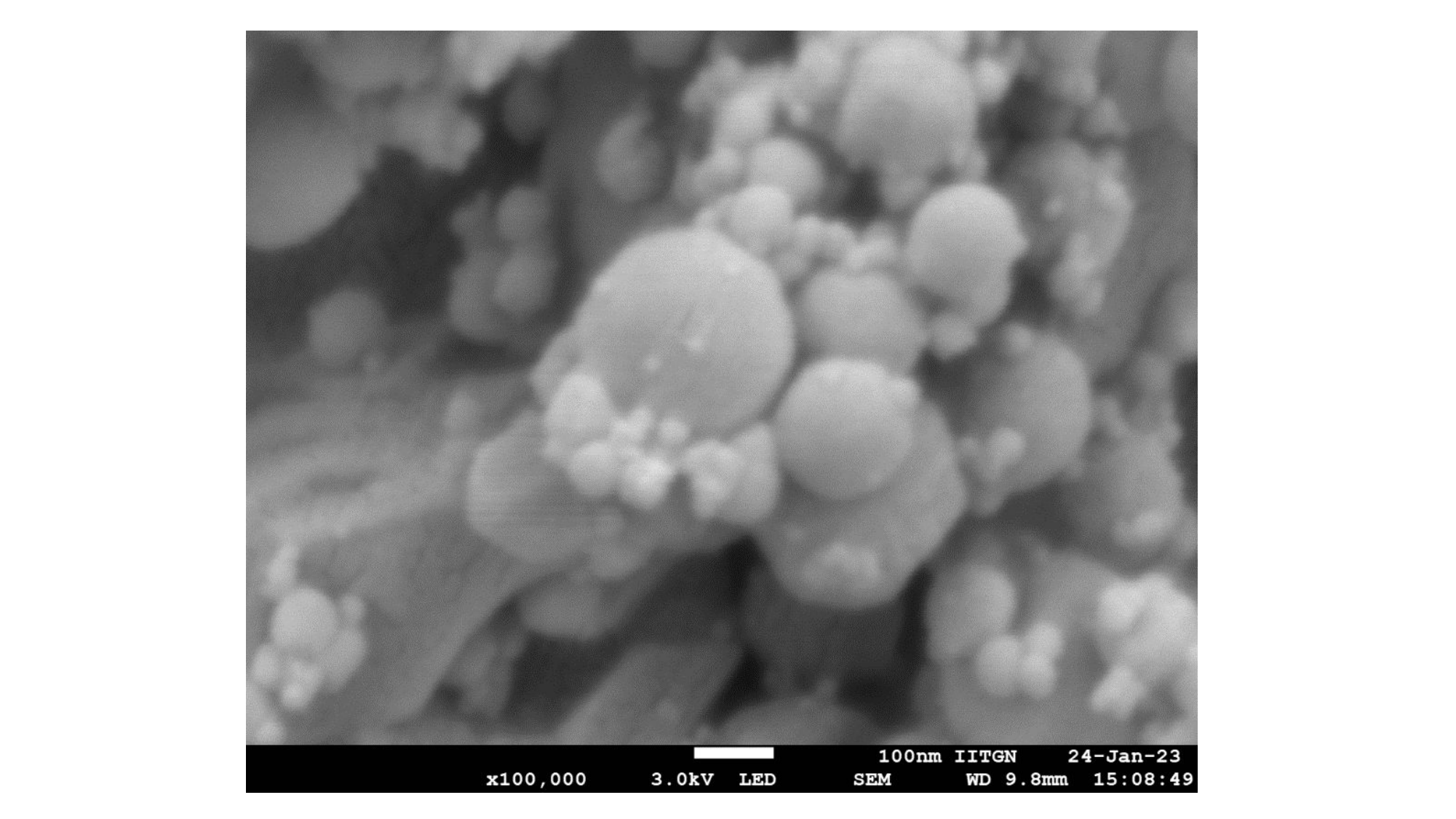

## Slide 12
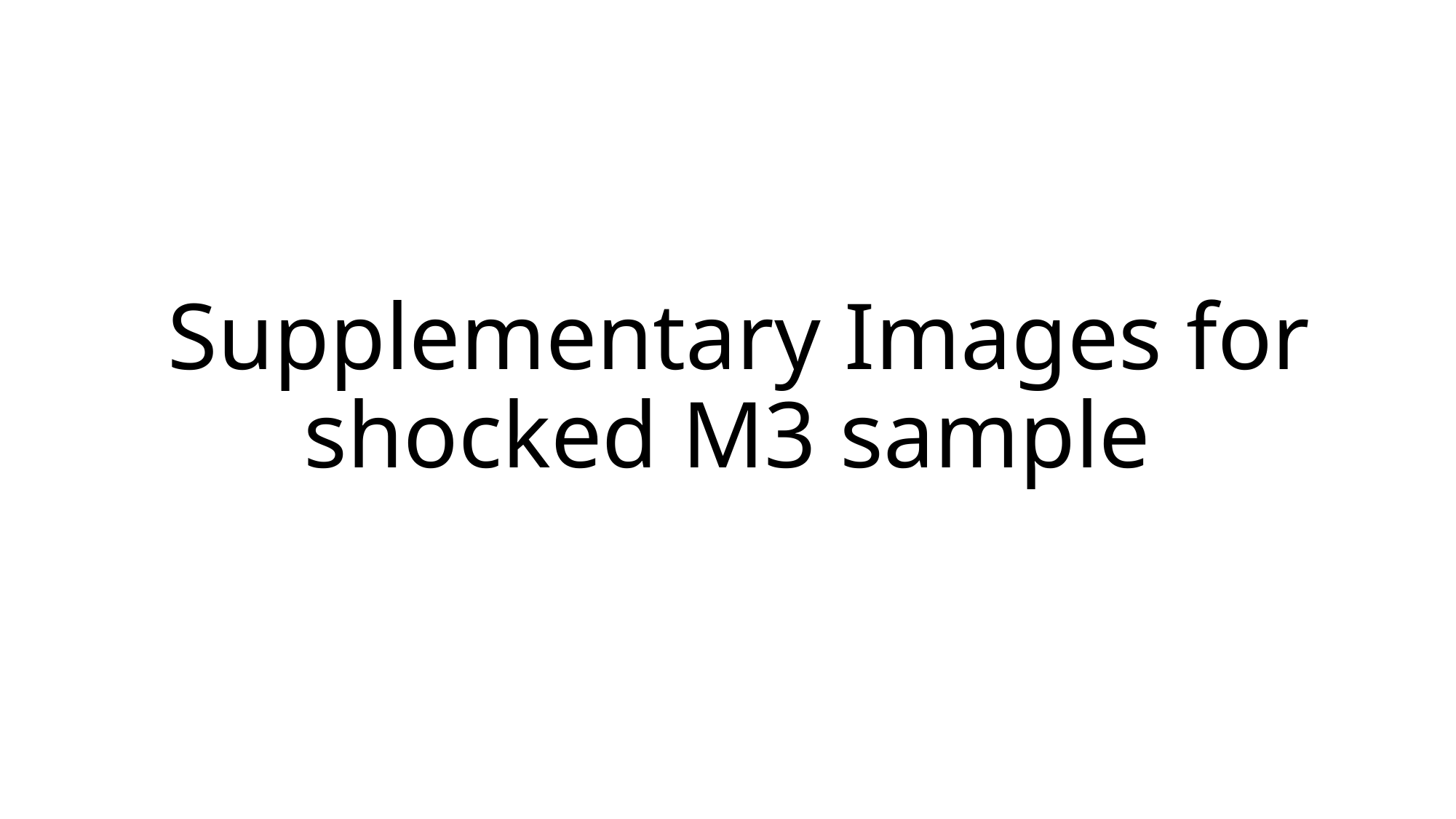

Supplementary Images for shocked M3 sample

## Slide 13
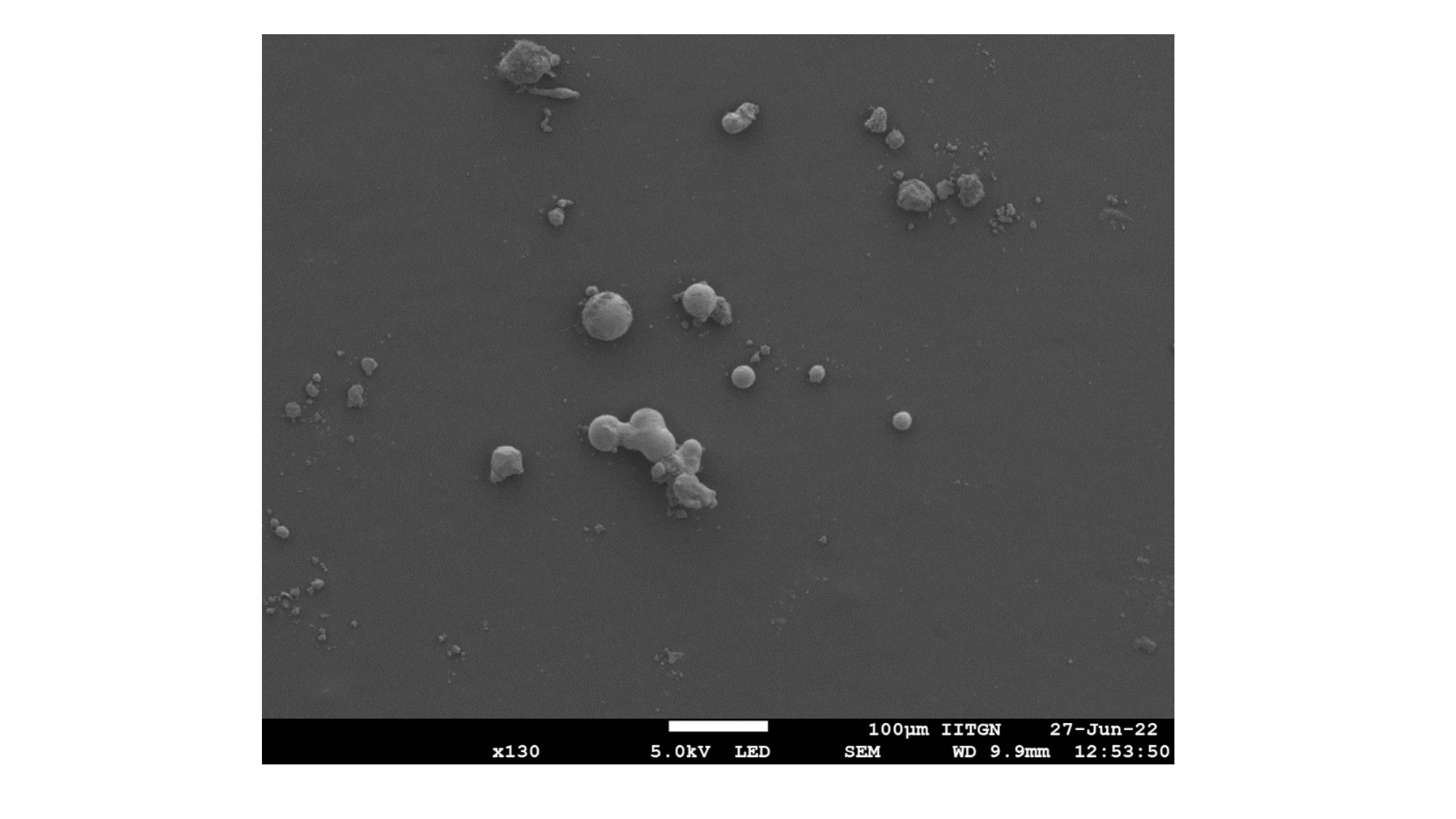

## Slide 14
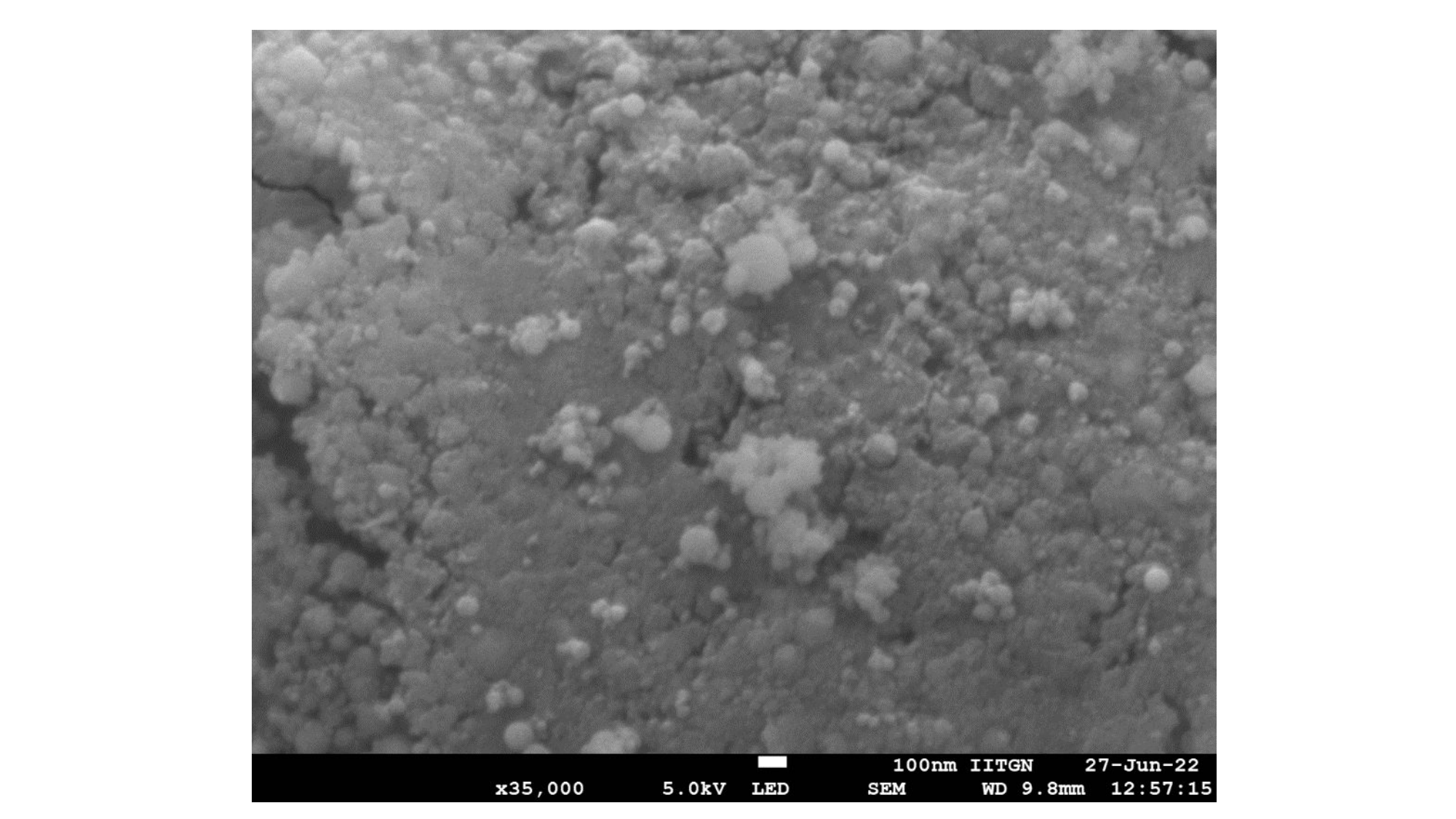

## Slide 15
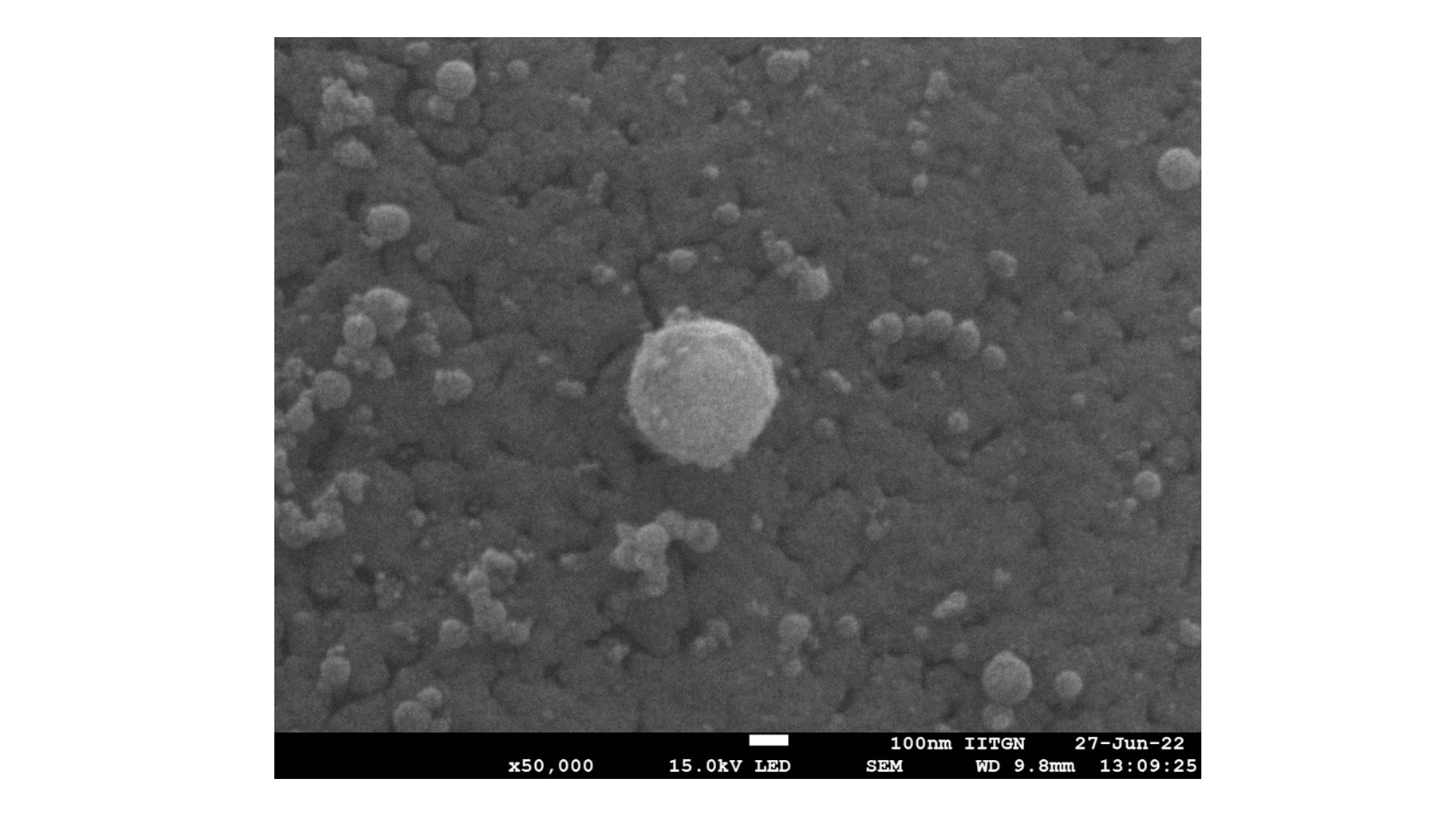

## Slide 16
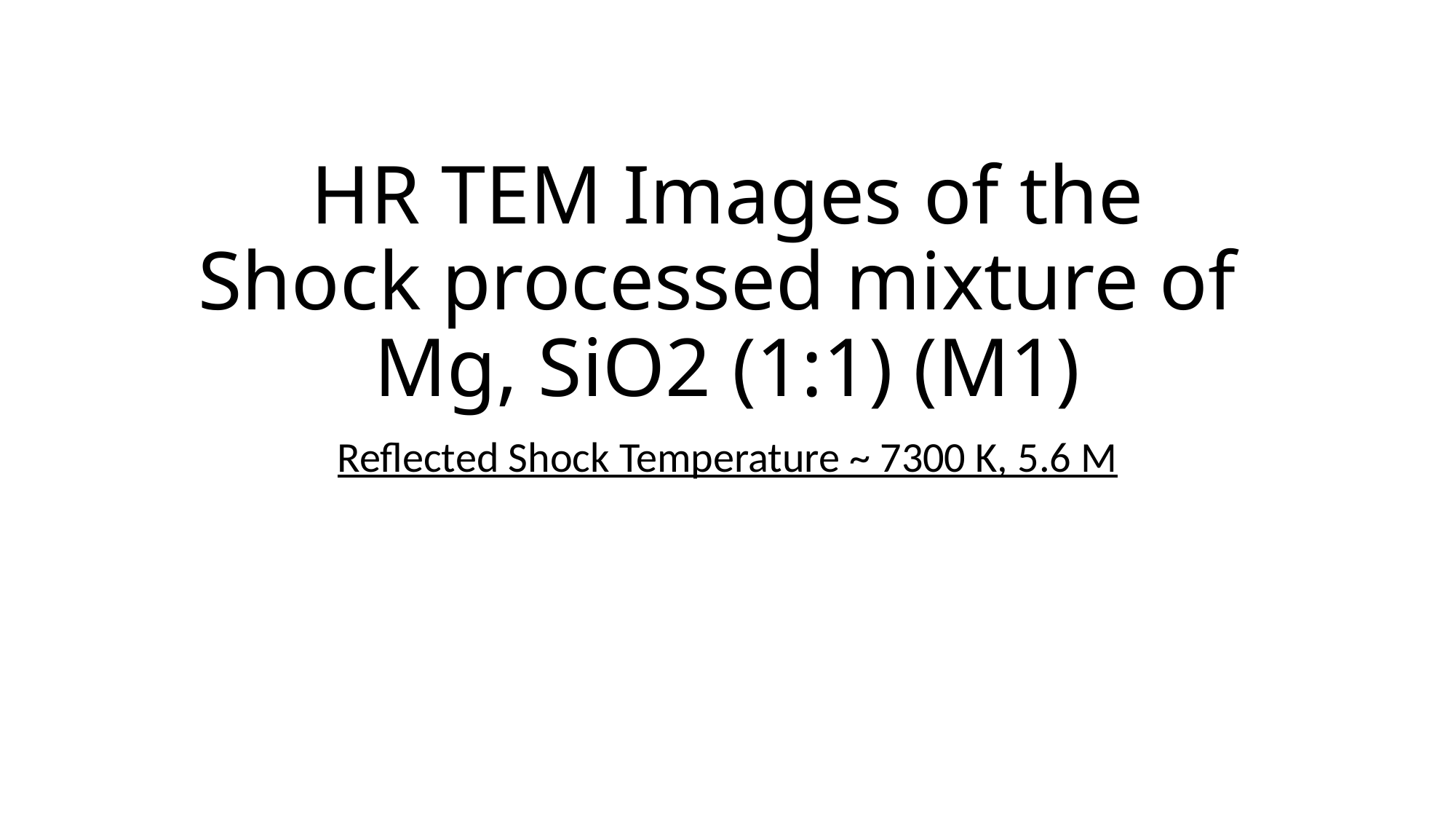

# HR TEM Images of the Shock processed mixture of Mg, SiO2 (1:1) (M1)
Reflected Shock Temperature ~ 7300 K, 5.6 M

## Slide 17
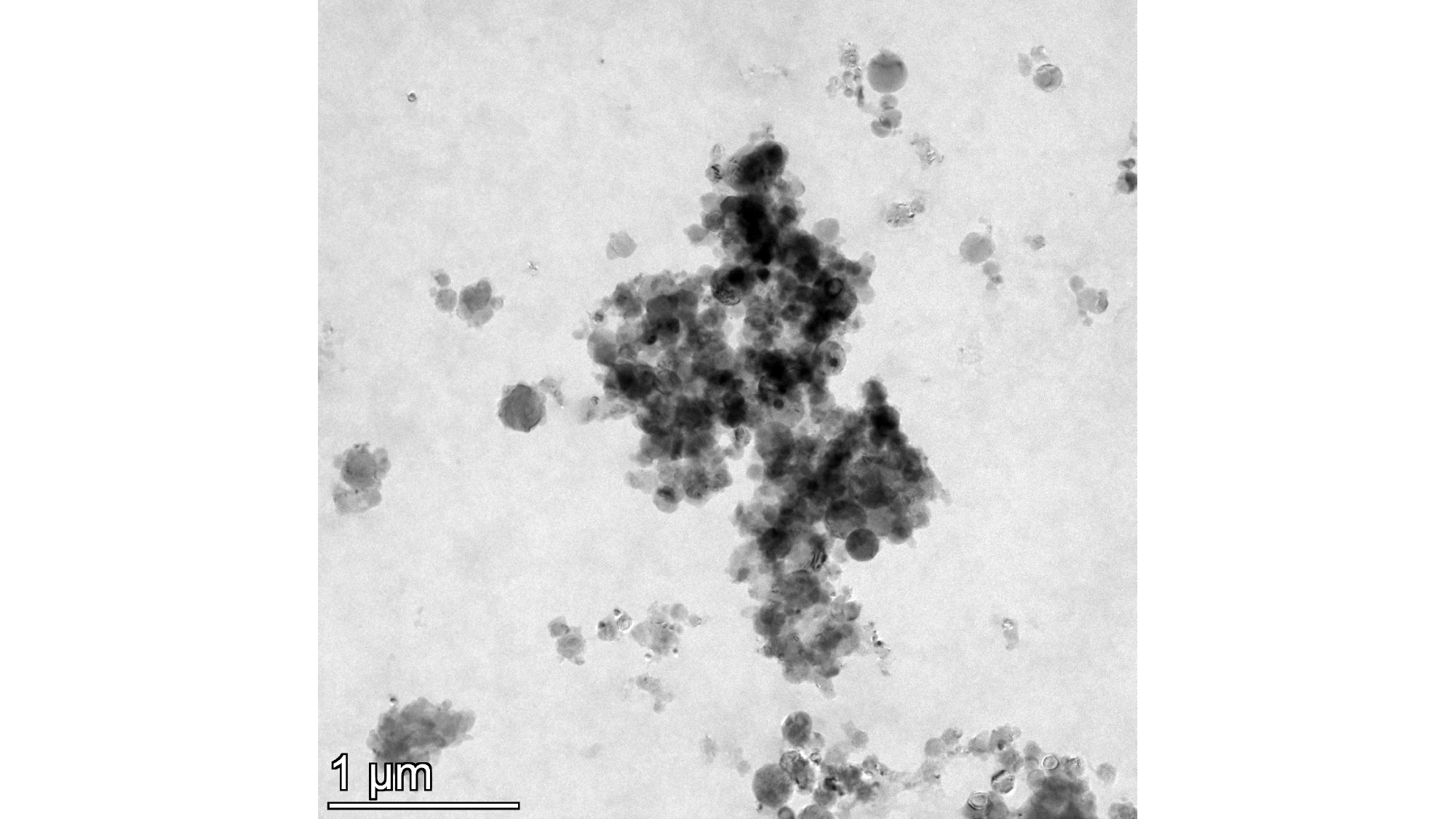

## Slide 18
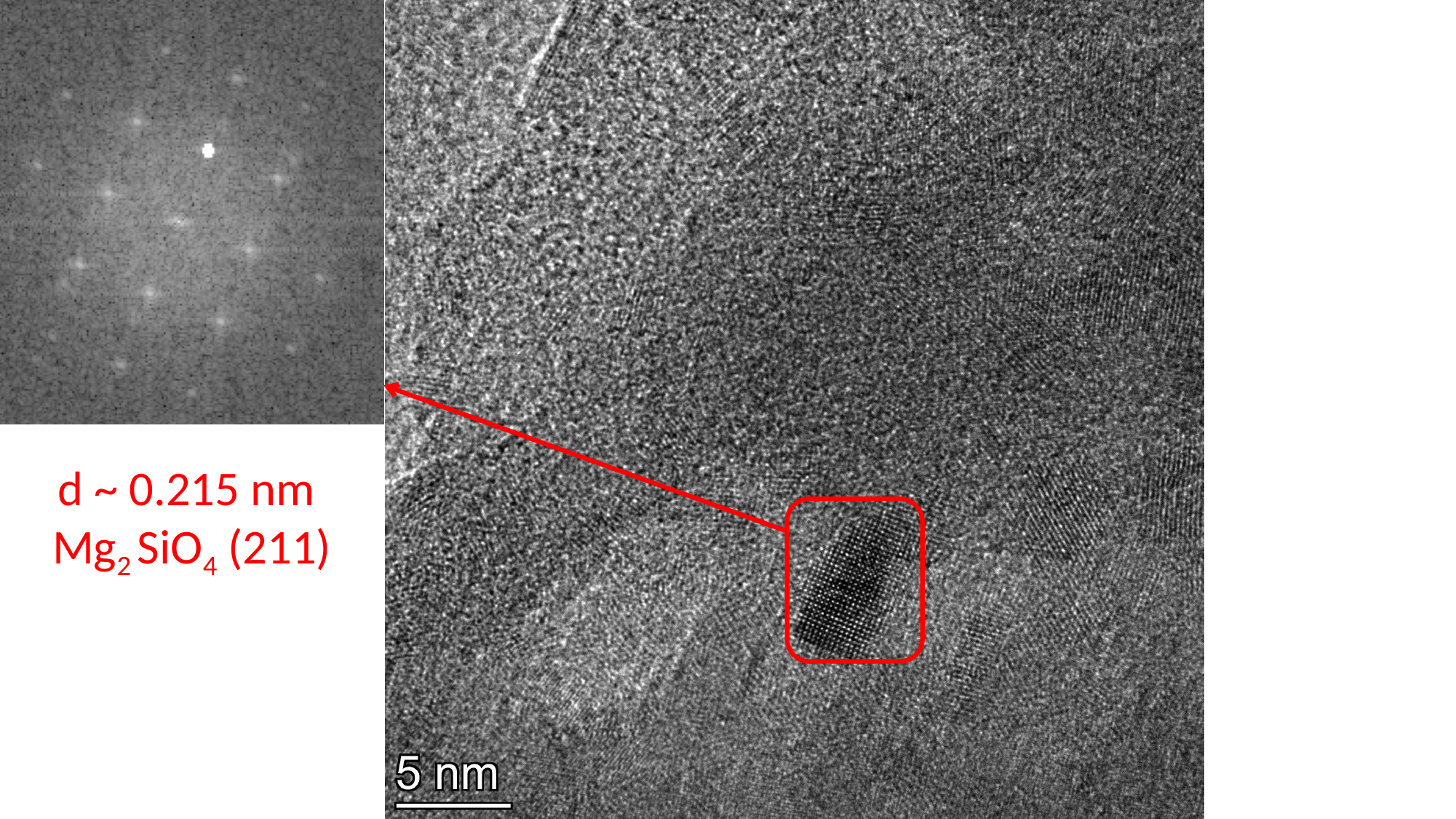

d ~ 0.215 nm
Mg2 SiO4 (211)

## Slide 19
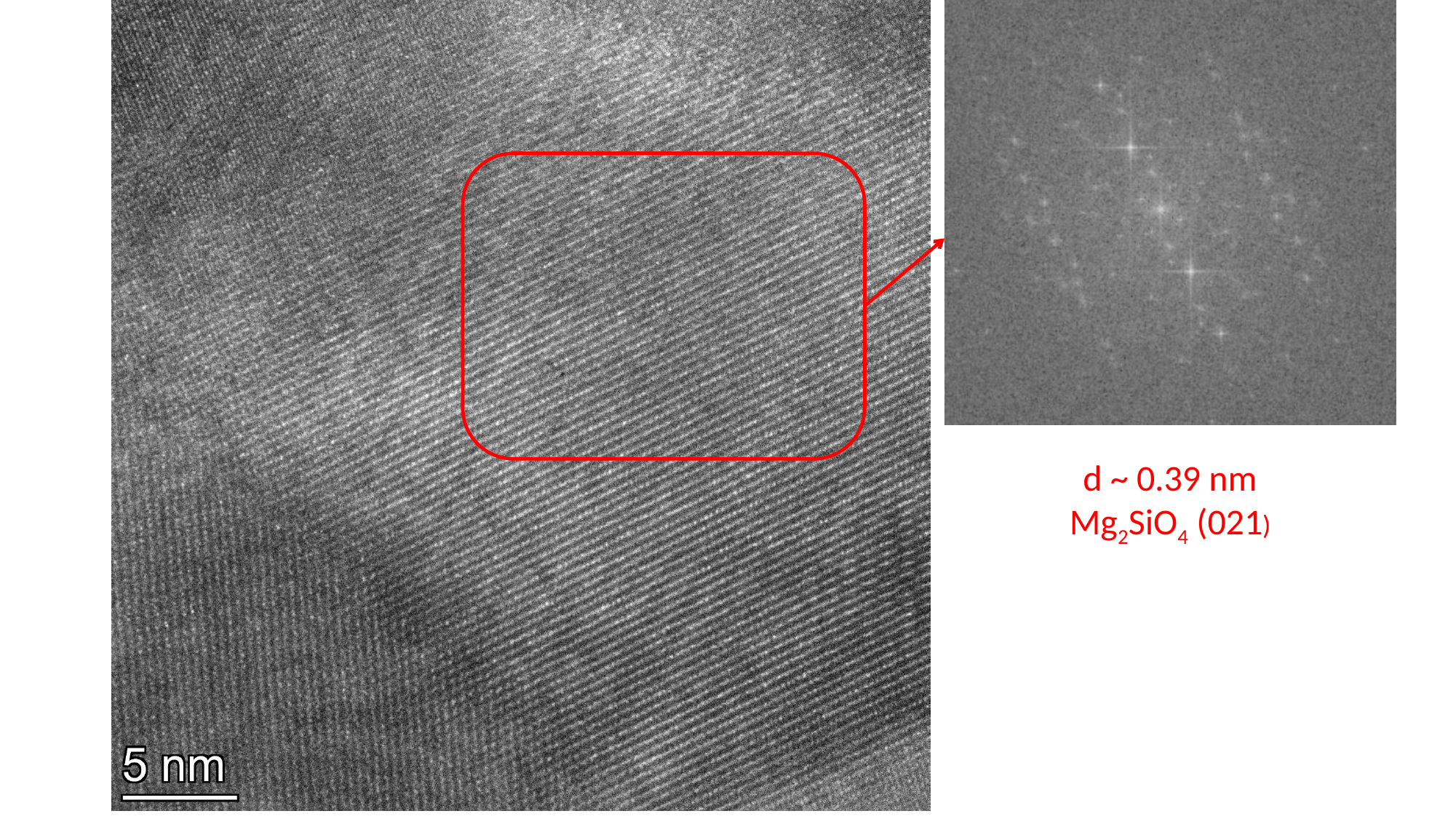

d ~ 0.39 nm
Mg2SiO4 (021)

## Slide 20
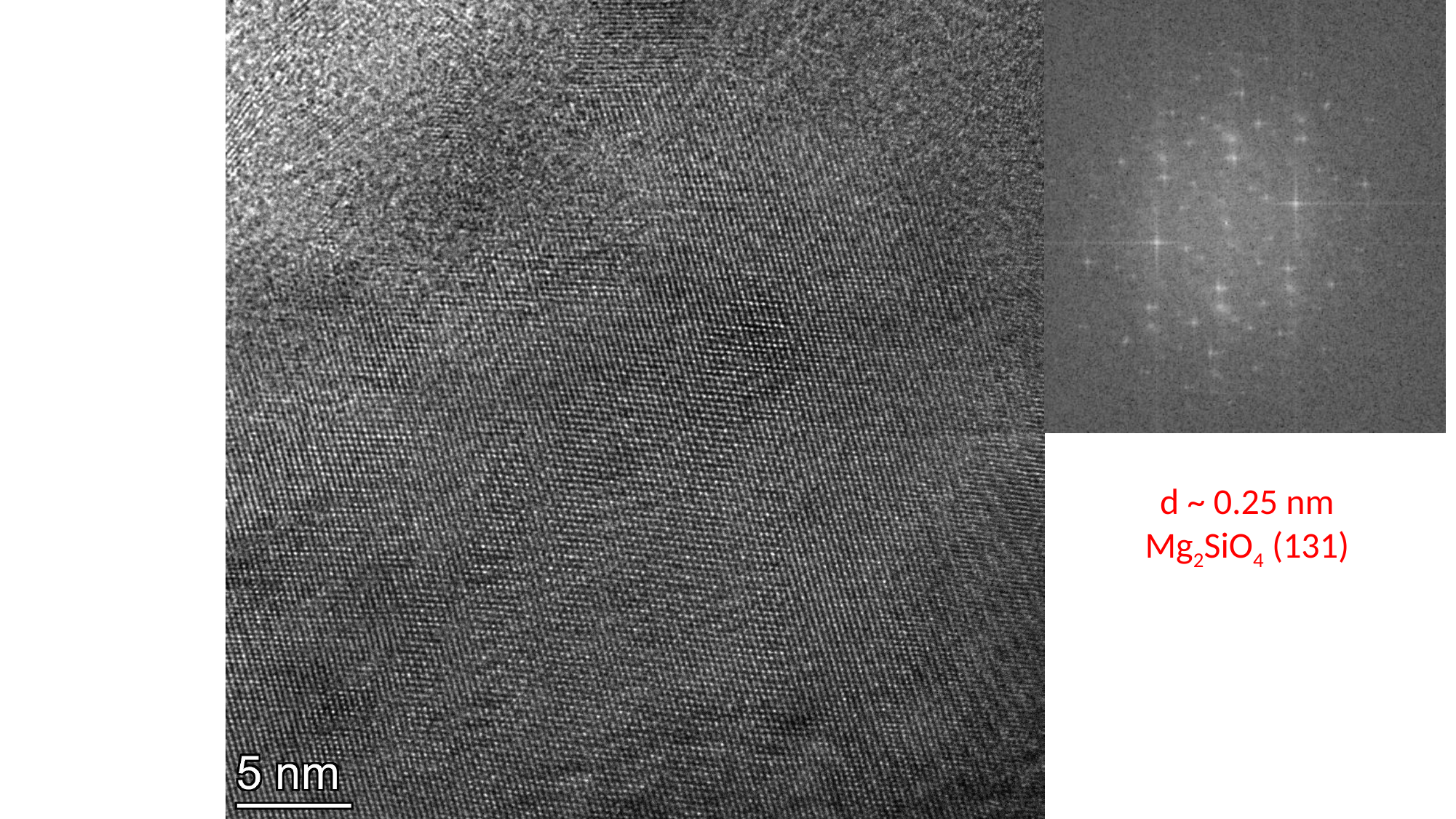

d ~ 0.25 nm
Mg2SiO4 (131)

## Slide 21
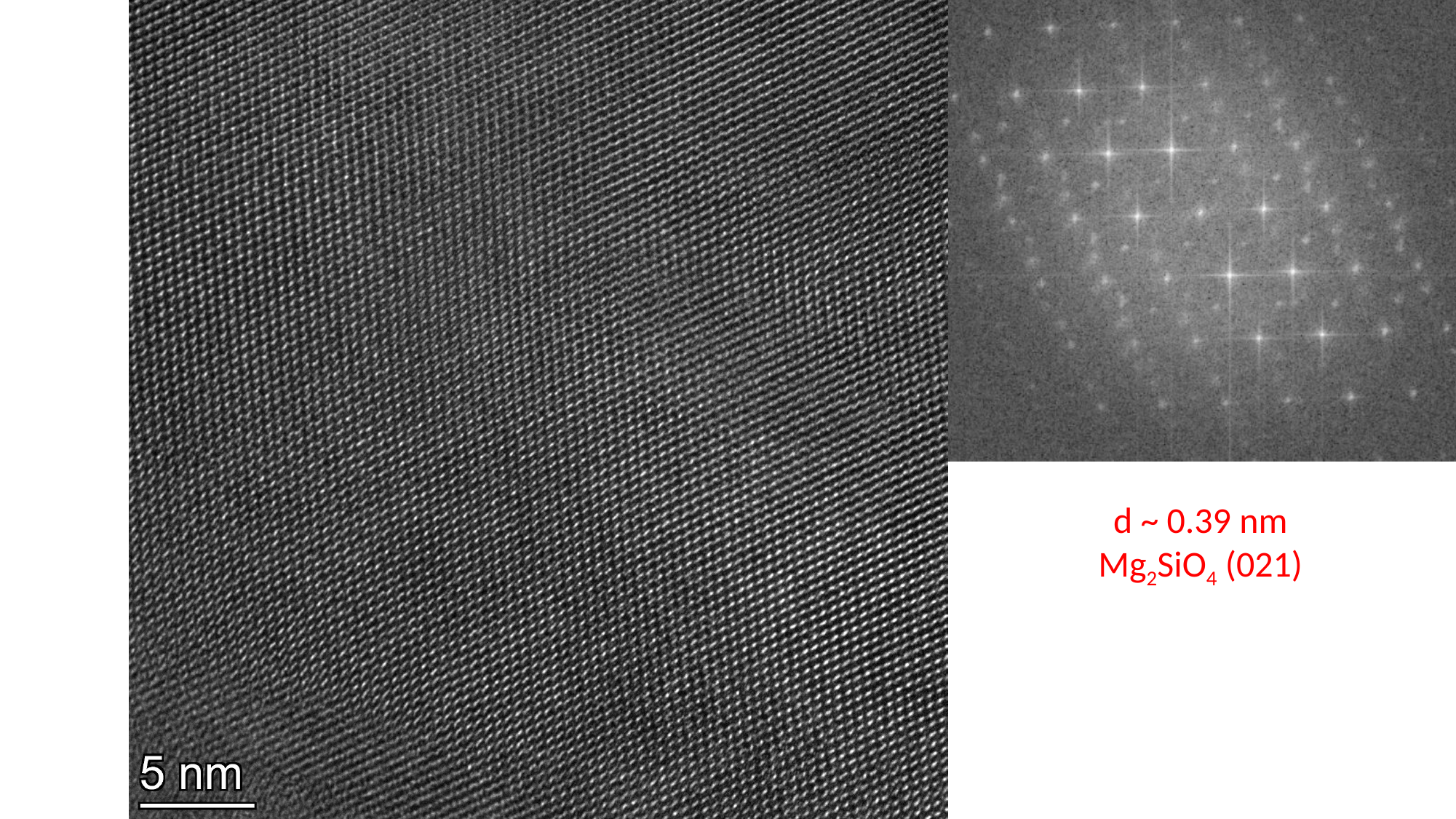

d ~ 0.39 nm
Mg2SiO4 (021)

## Slide 22
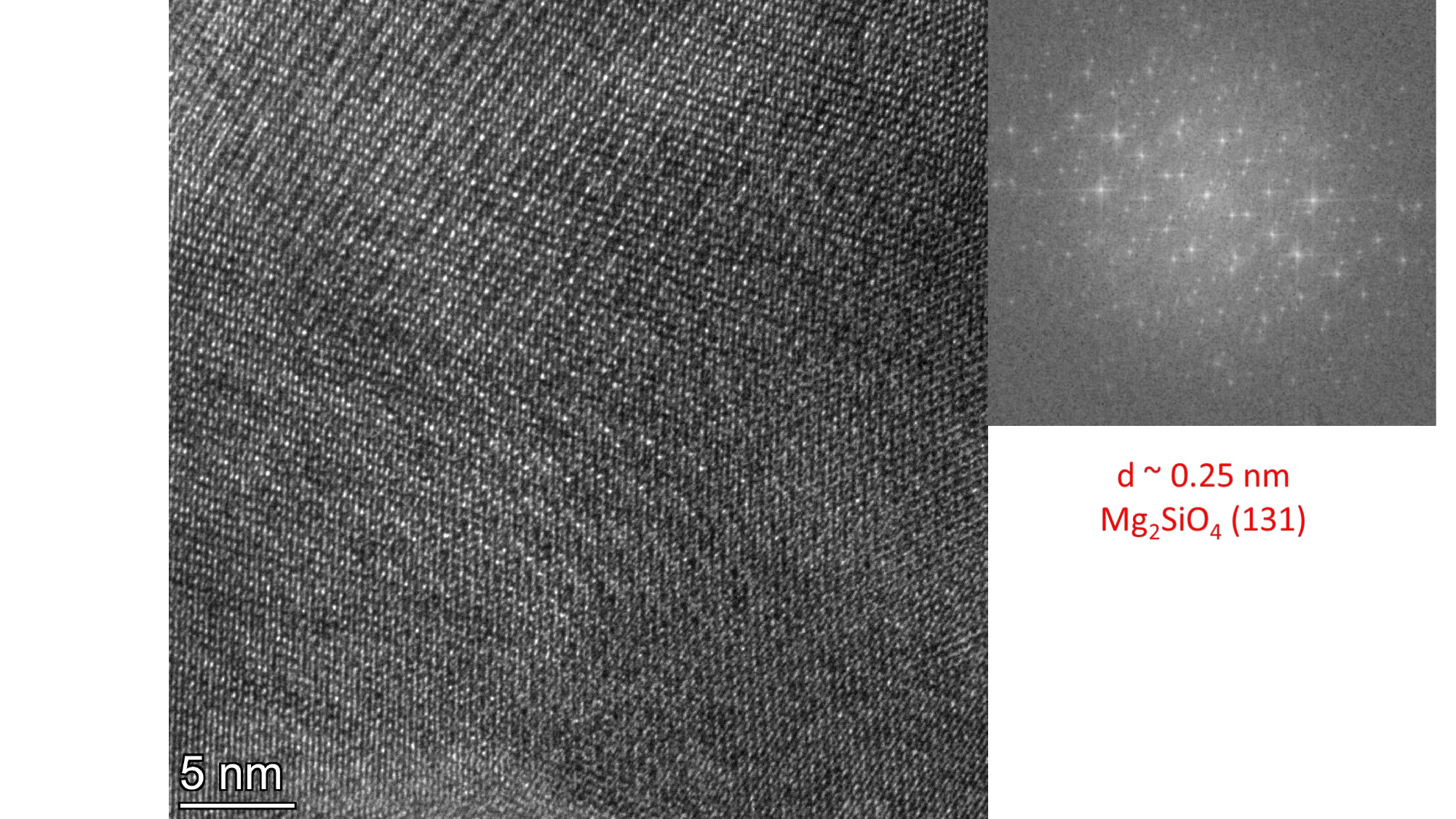

## Slide 23
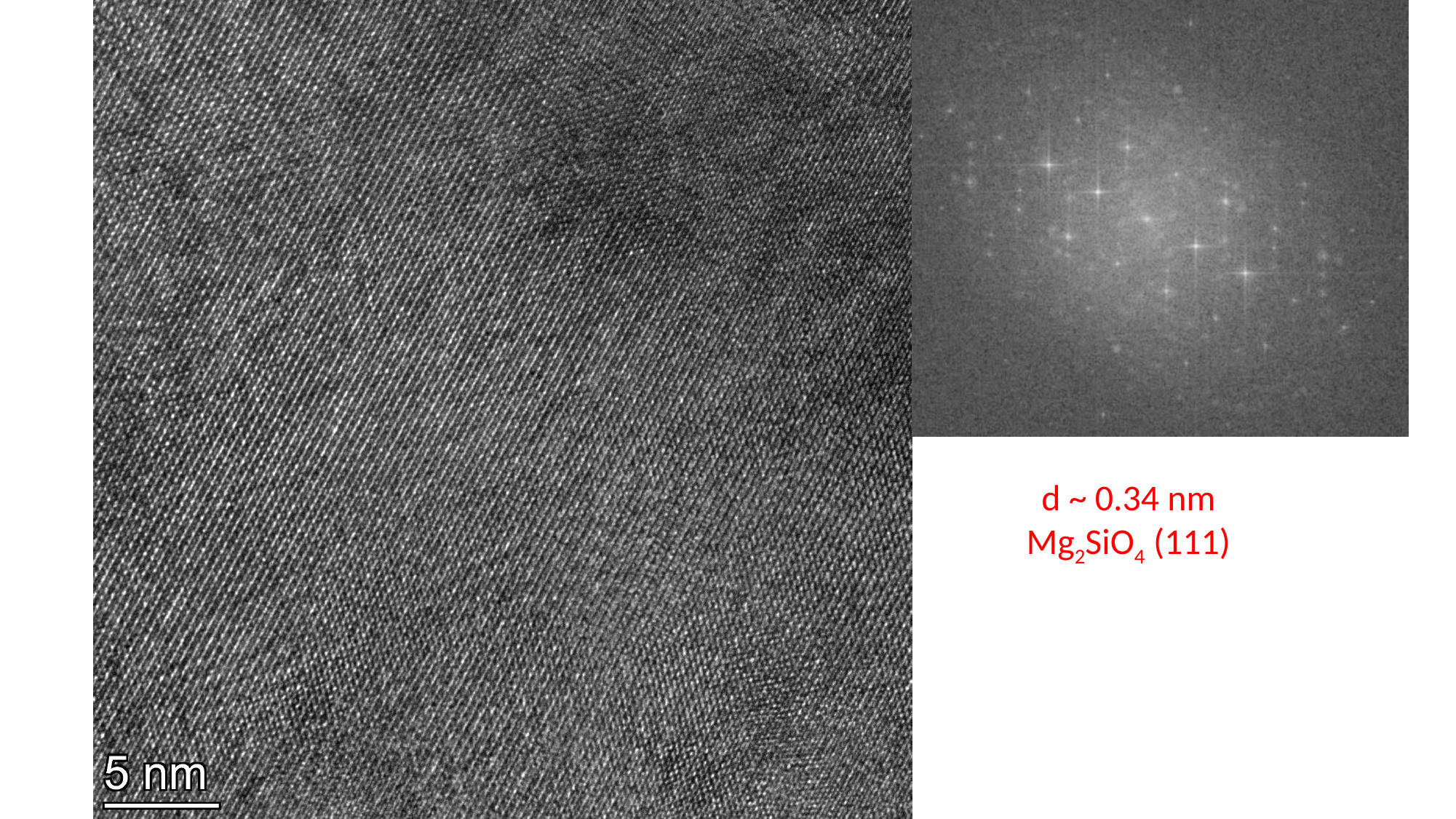

d ~ 0.34 nm
Mg2SiO4 (111)

## Slide 24
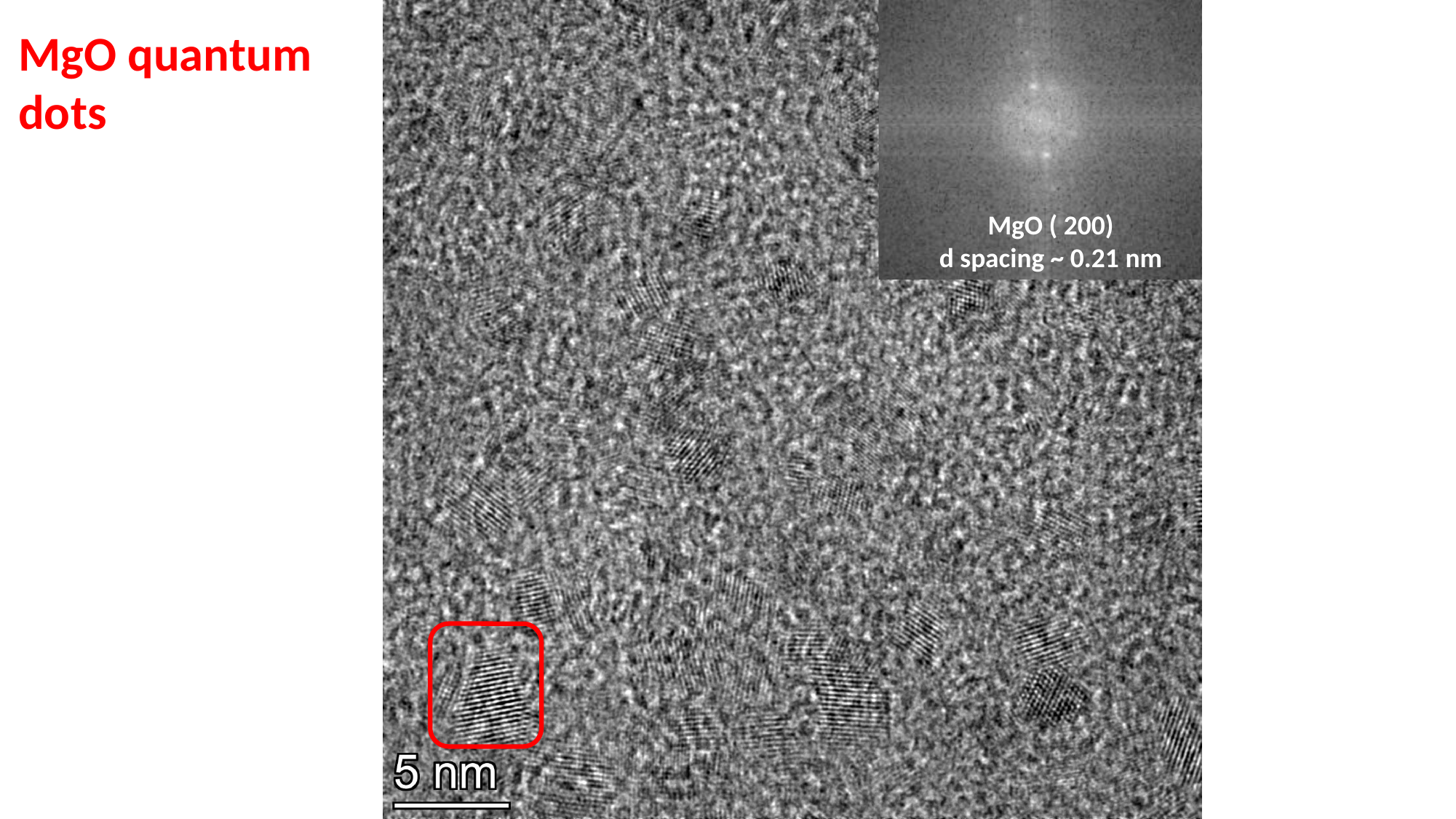

MgO ( 200)
d spacing ~ 0.21 nm
MgO quantum dots
FFT

## Slide 25
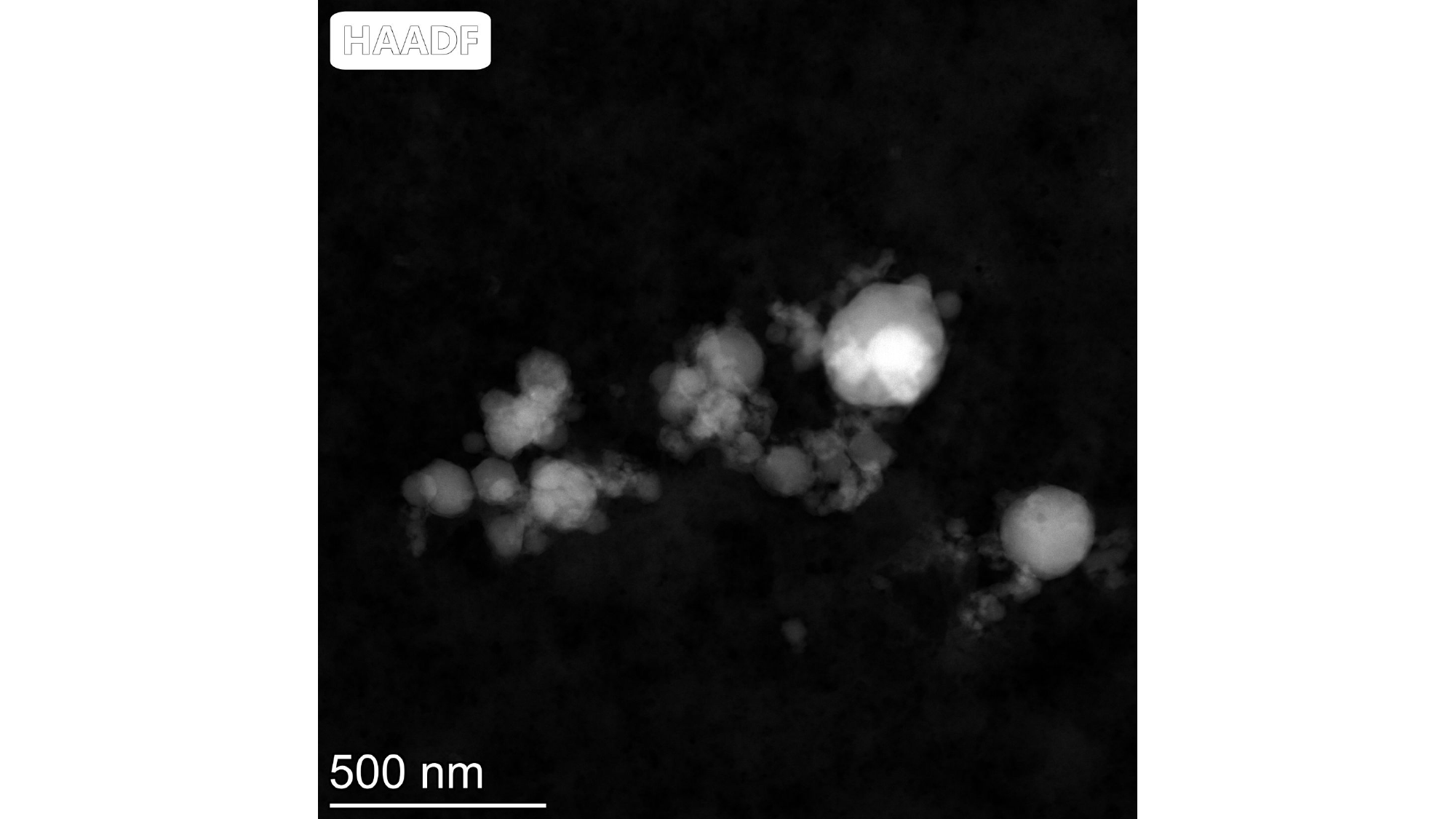

## Slide 26
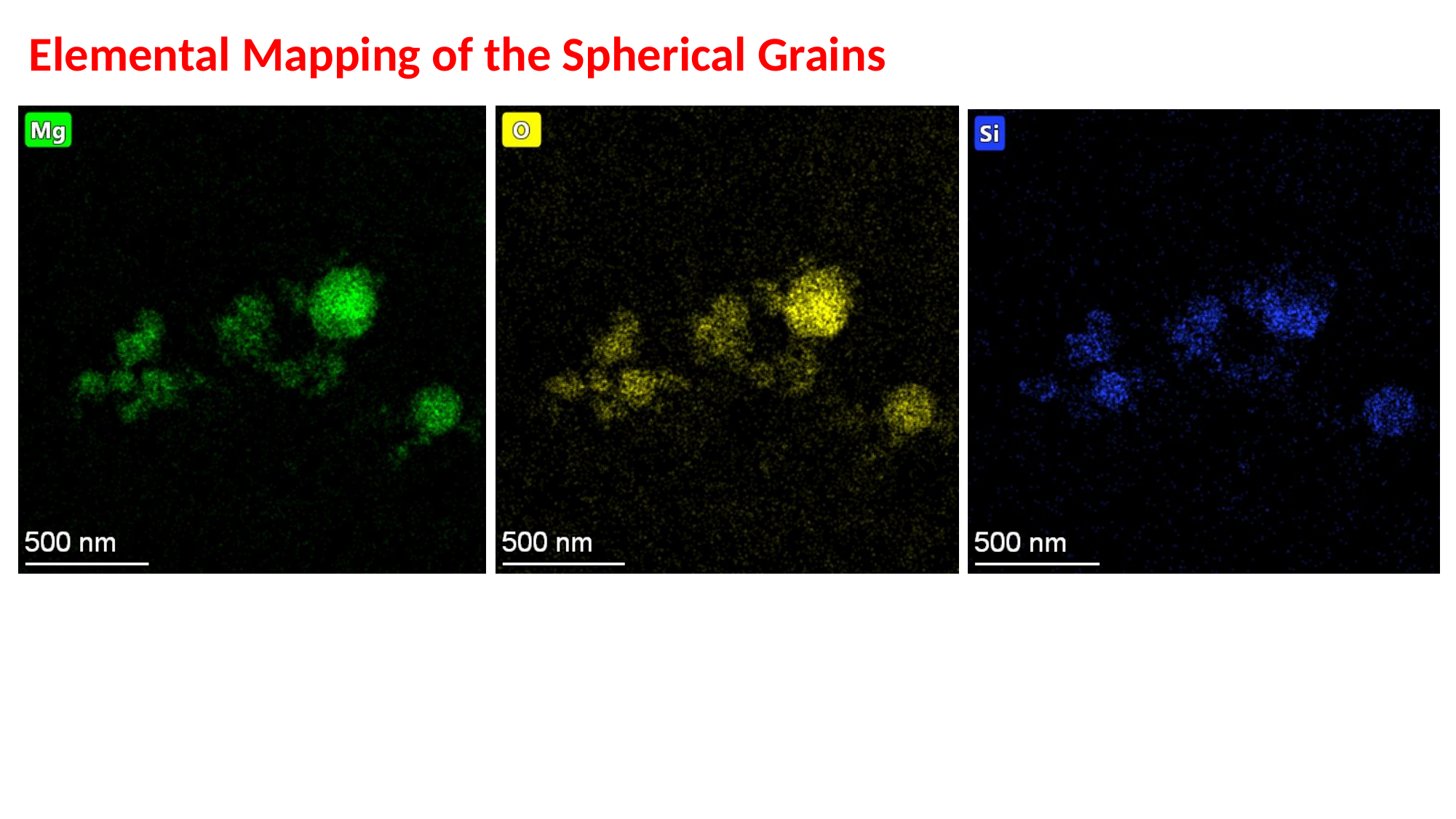

Elemental Mapping of the Spherical Grains

## Slide 27
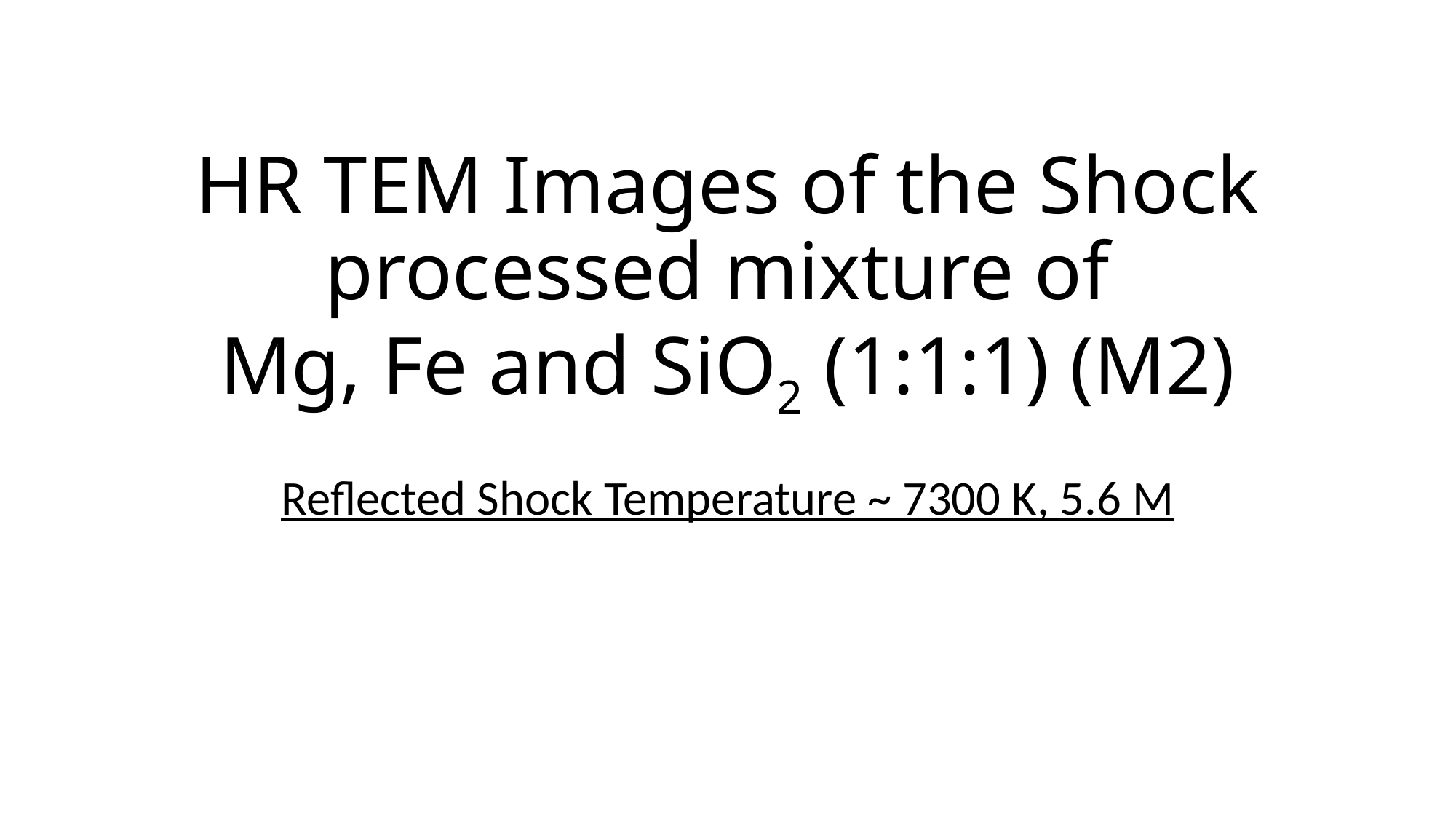

# HR TEM Images of the Shock processed mixture of Mg, Fe and SiO2 (1:1:1) (M2)
Reflected Shock Temperature ~ 7300 K, 5.6 M

## Slide 28
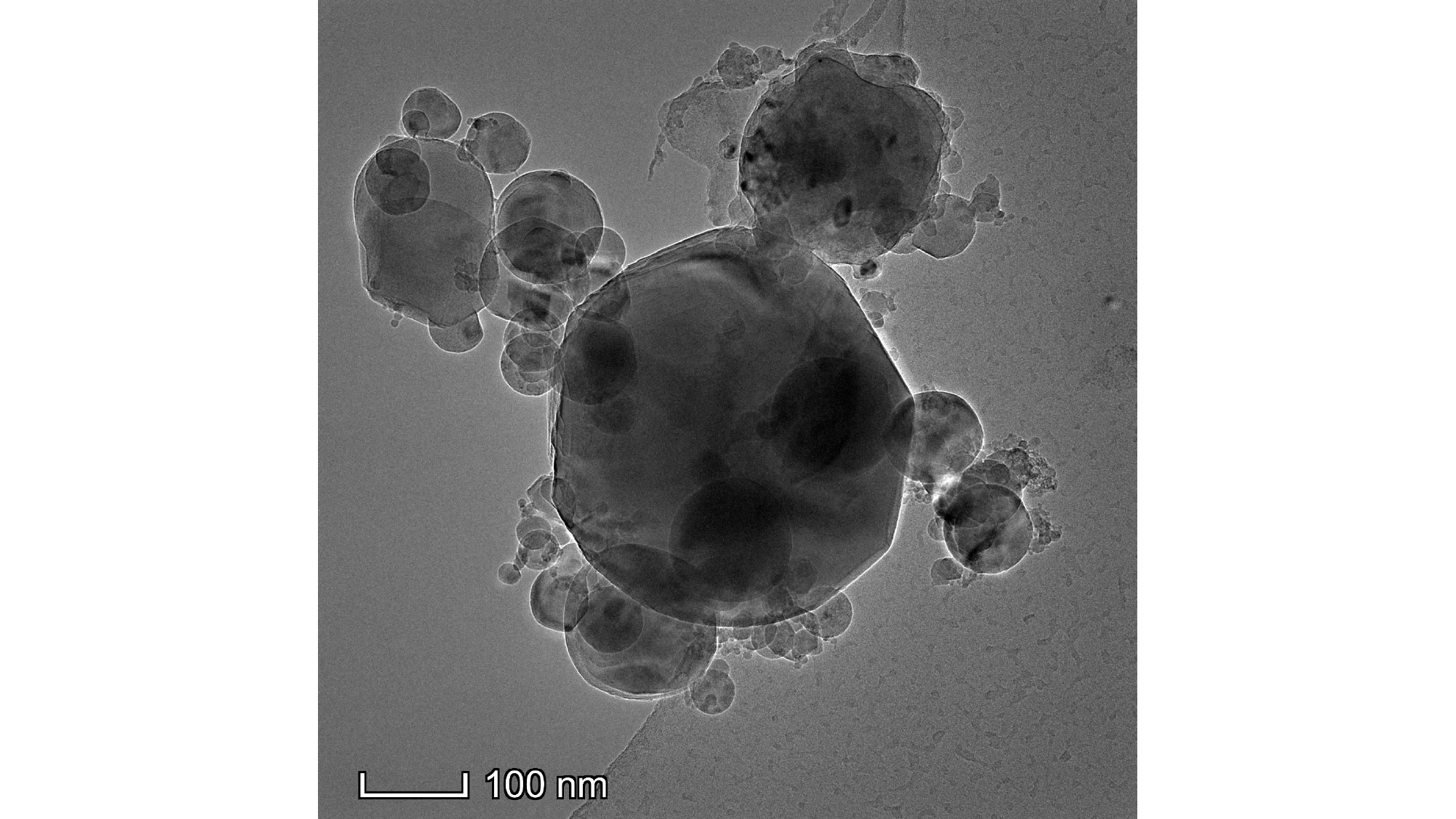

## Slide 29
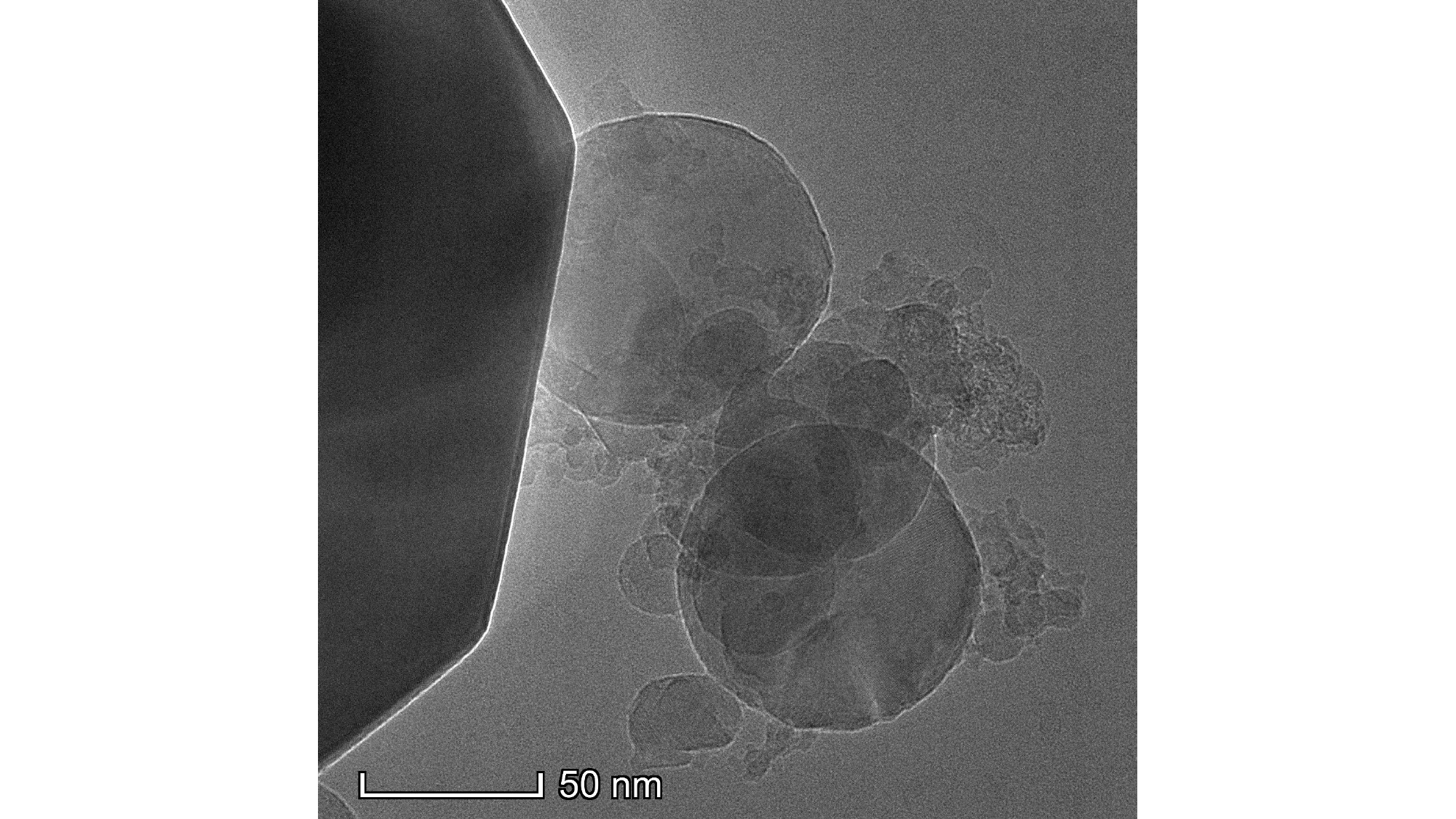

## Slide 30
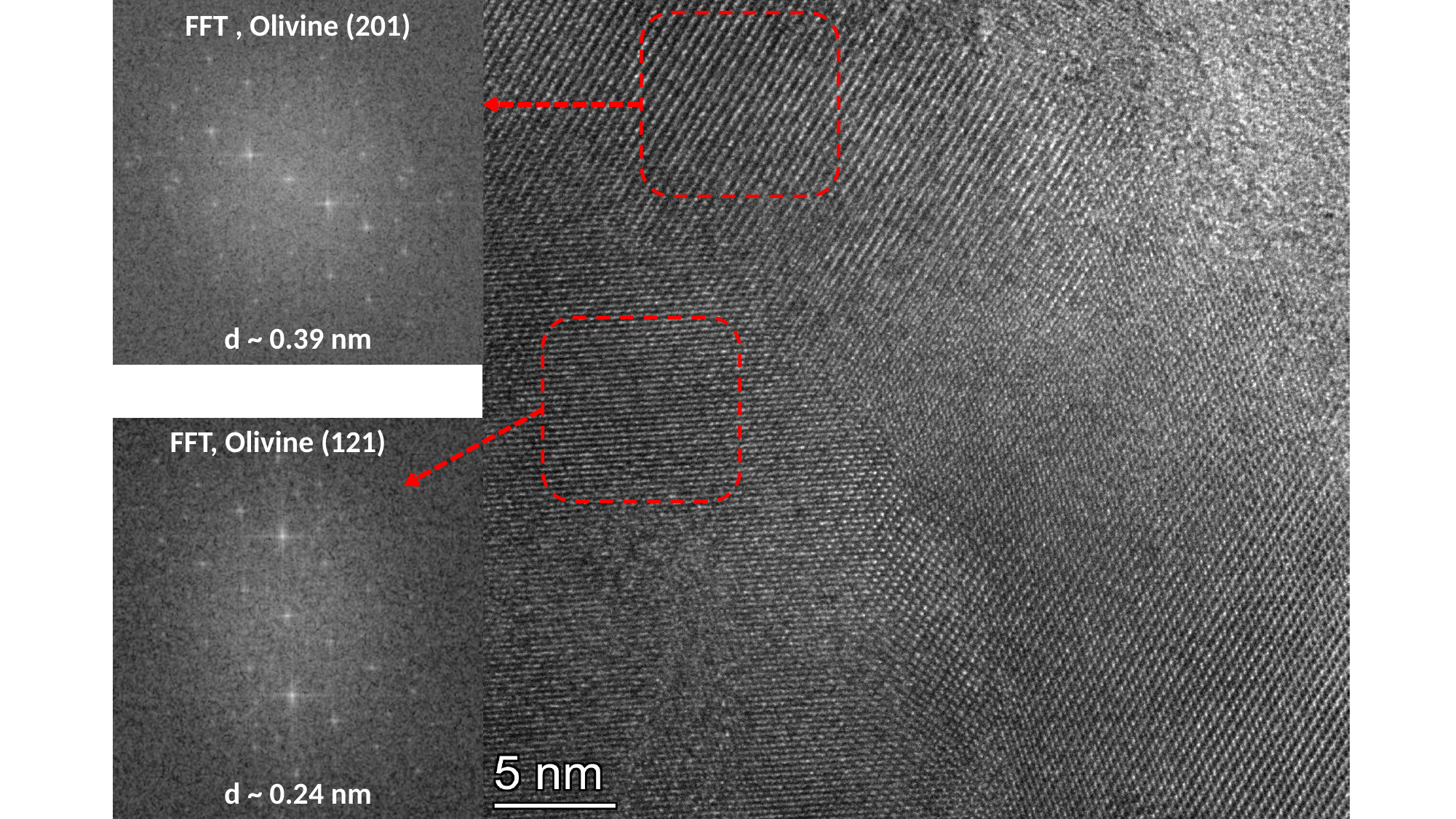

FFT , Olivine (201)
d ~ 0.39 nm
FFT, Olivine (121)
d ~ 0.24 nm

## Slide 31
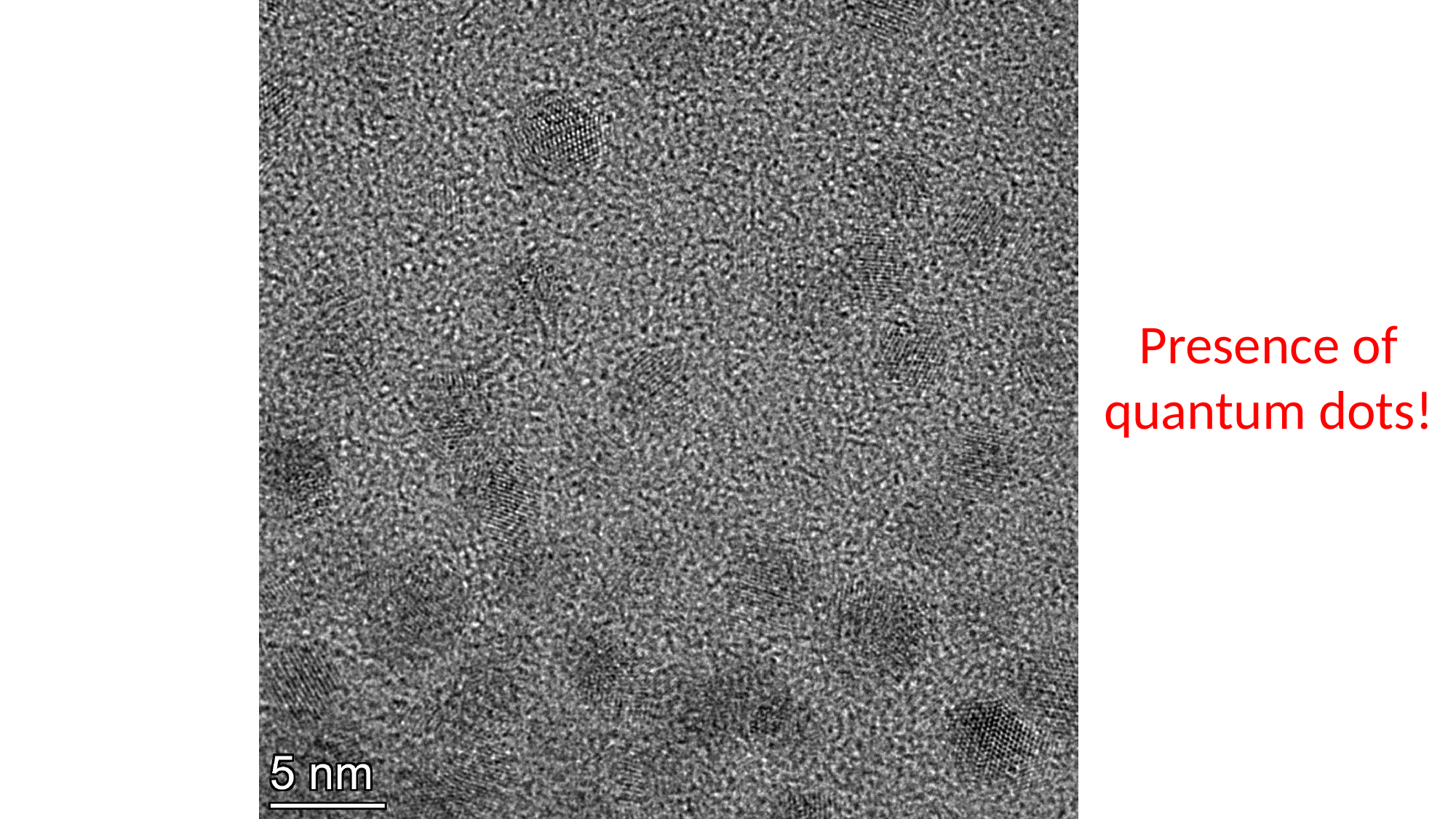

Presence of quantum dots!

## Slide 32
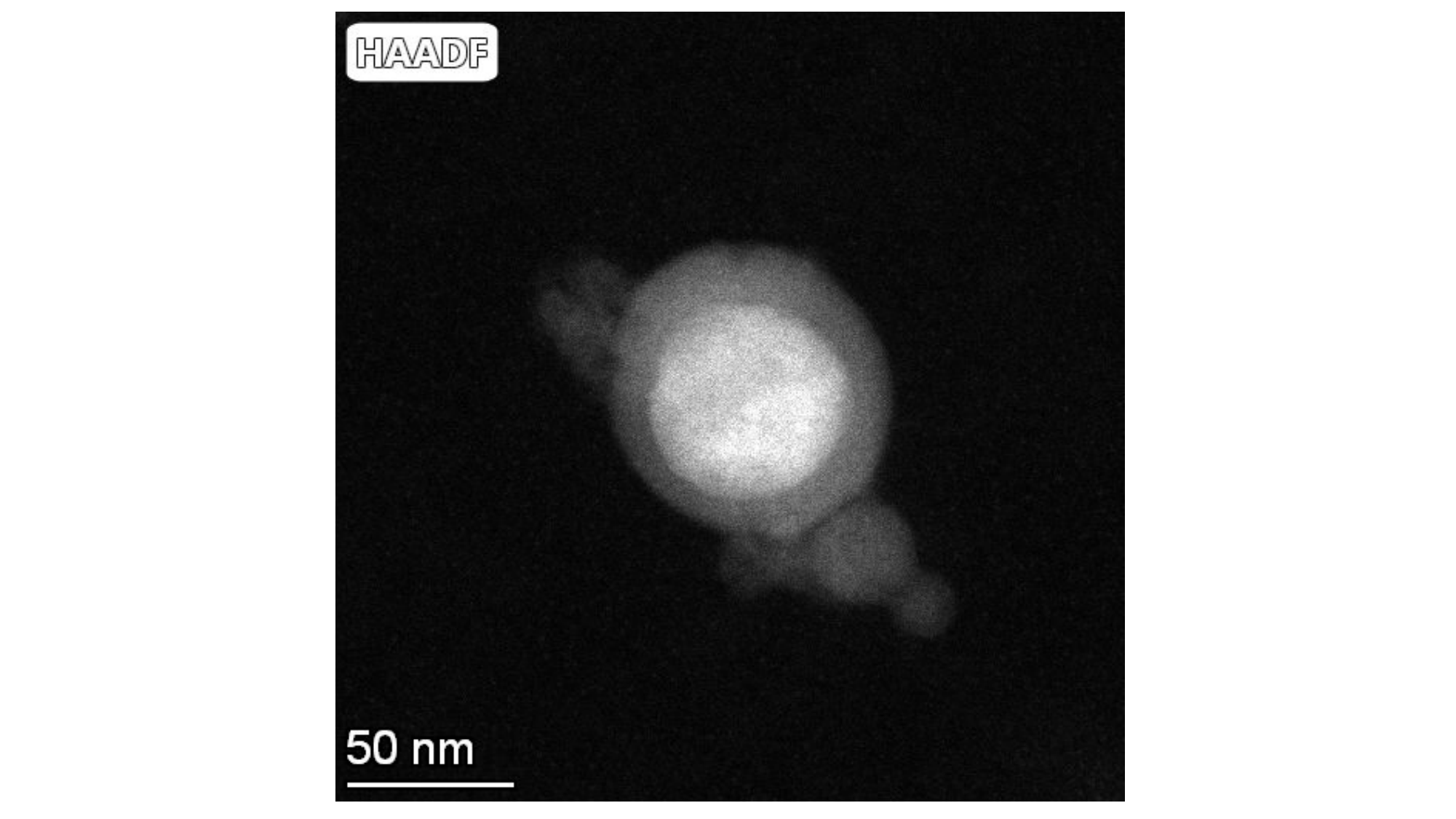

## Slide 33
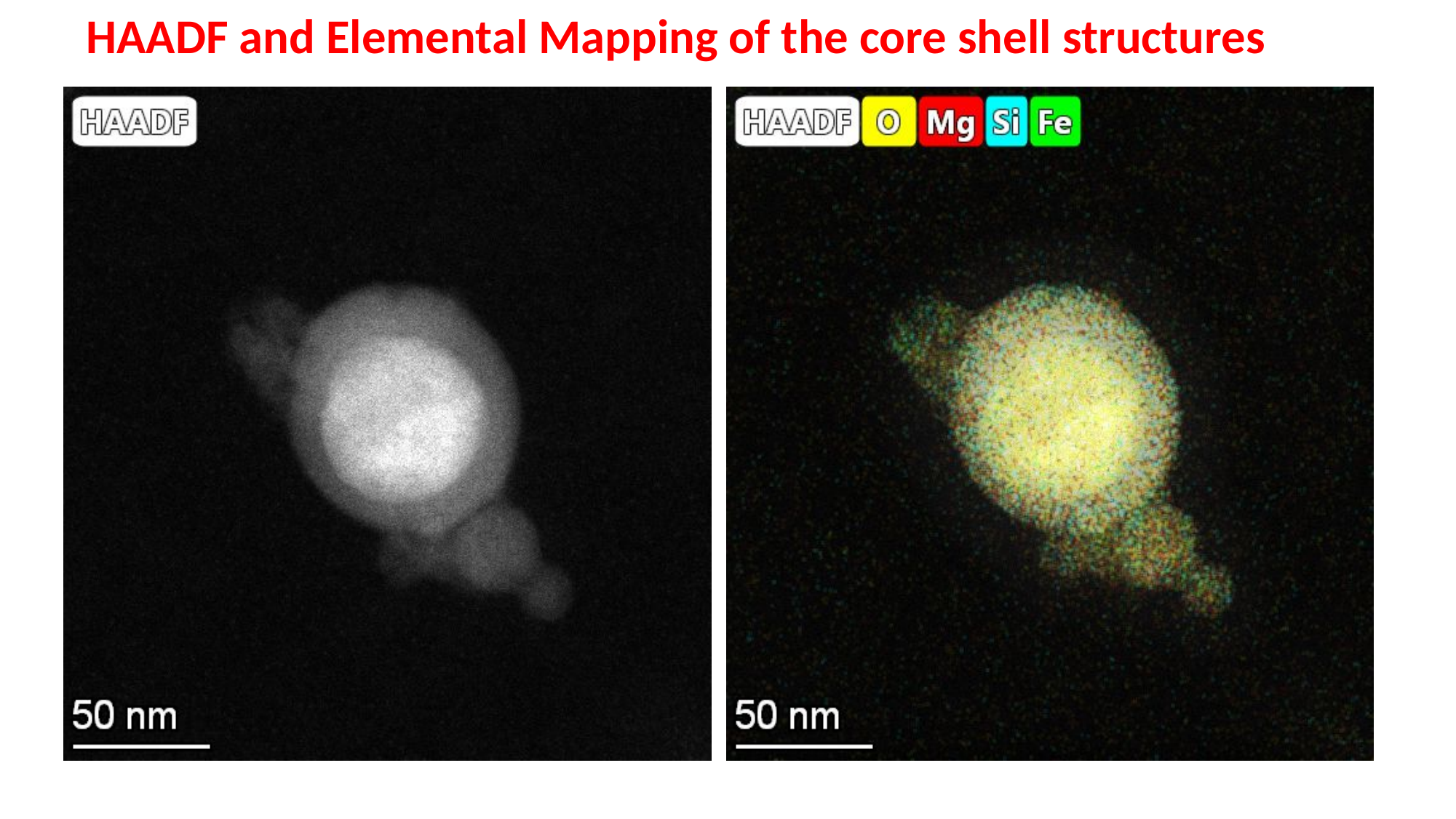

HAADF and Elemental Mapping of the core shell structures

## Slide 34
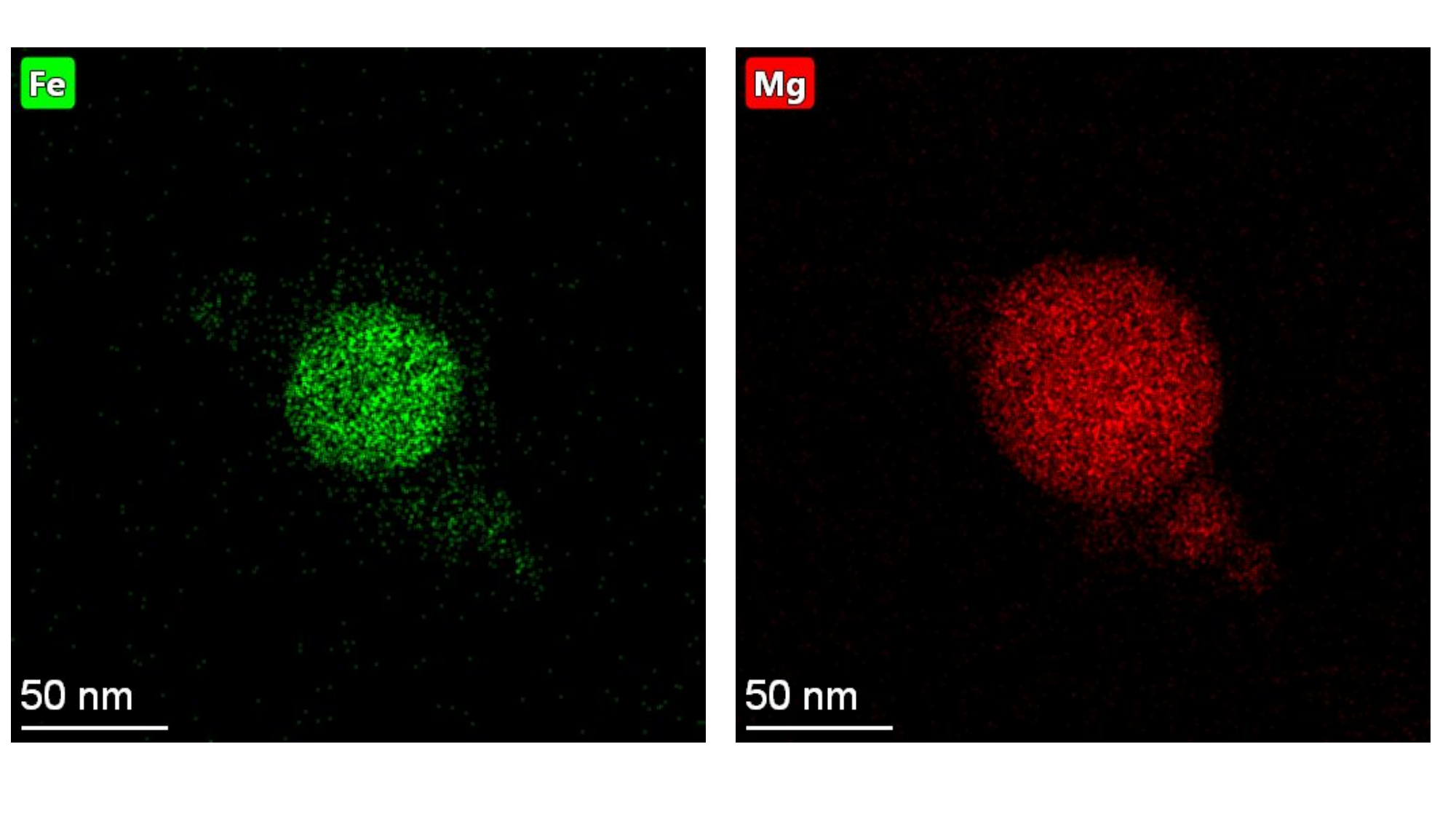

## Slide 35
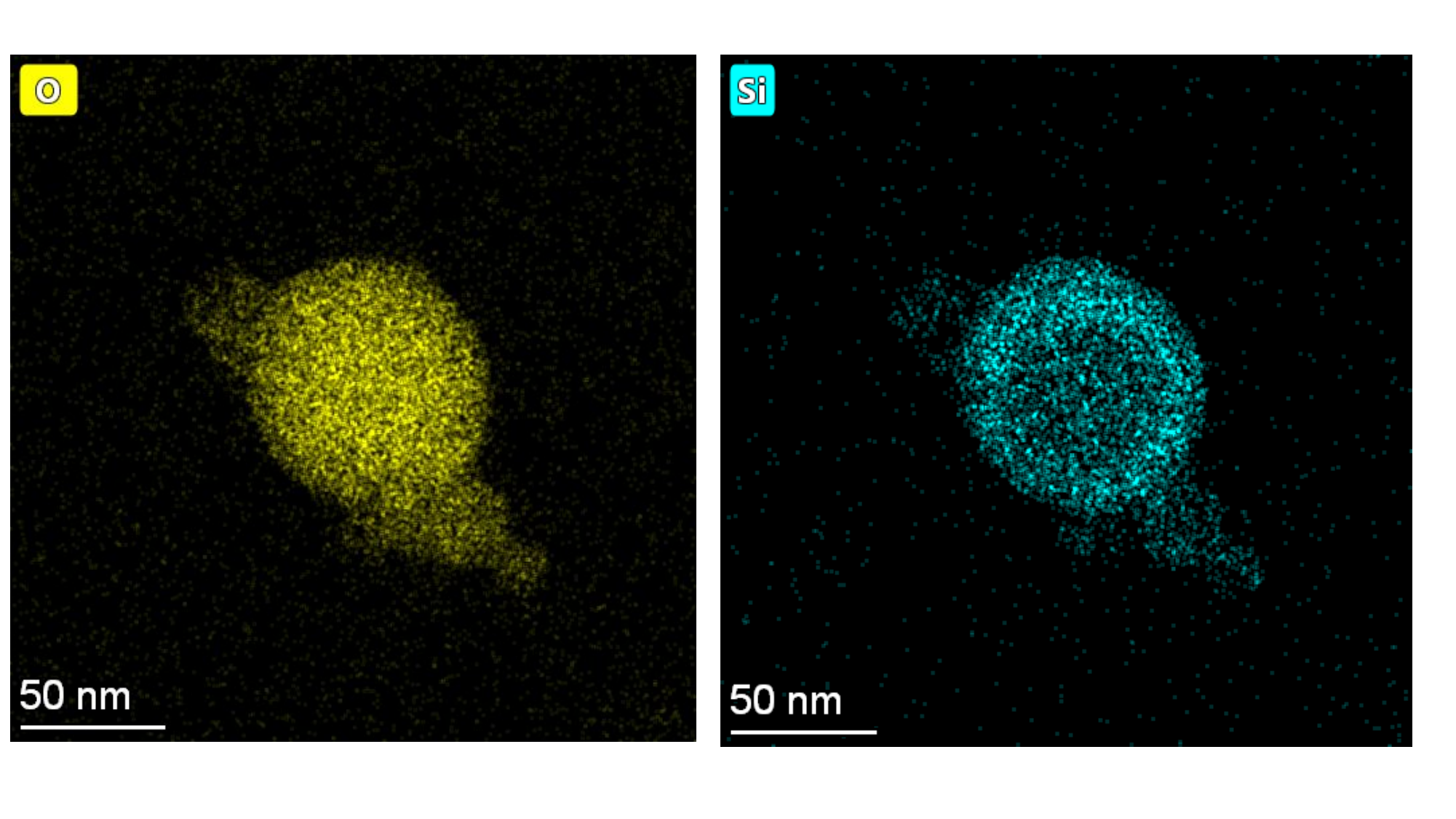

## Slide 36
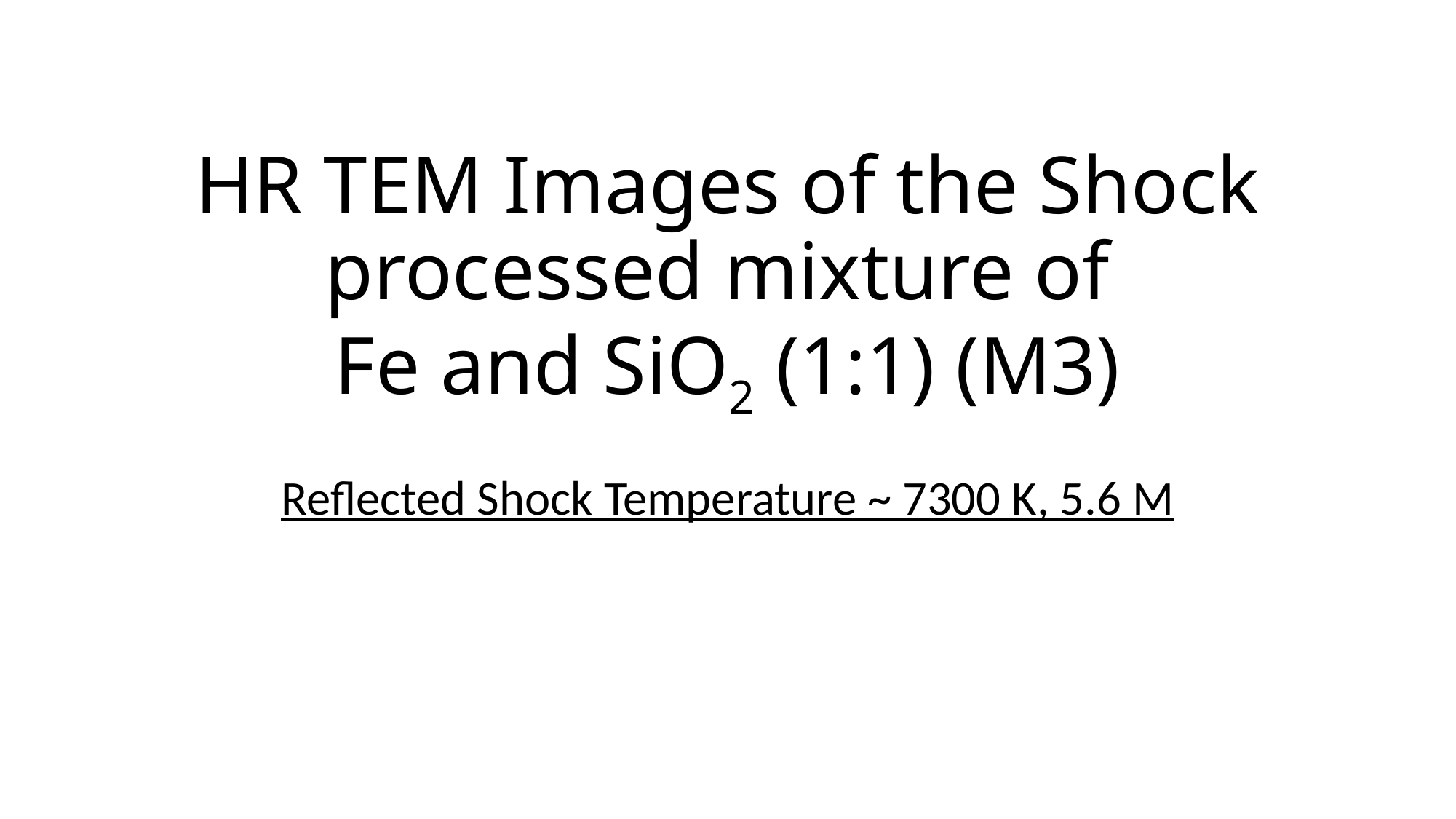

# HR TEM Images of the Shock processed mixture of Fe and SiO2 (1:1) (M3)
Reflected Shock Temperature ~ 7300 K, 5.6 M

## Slide 37
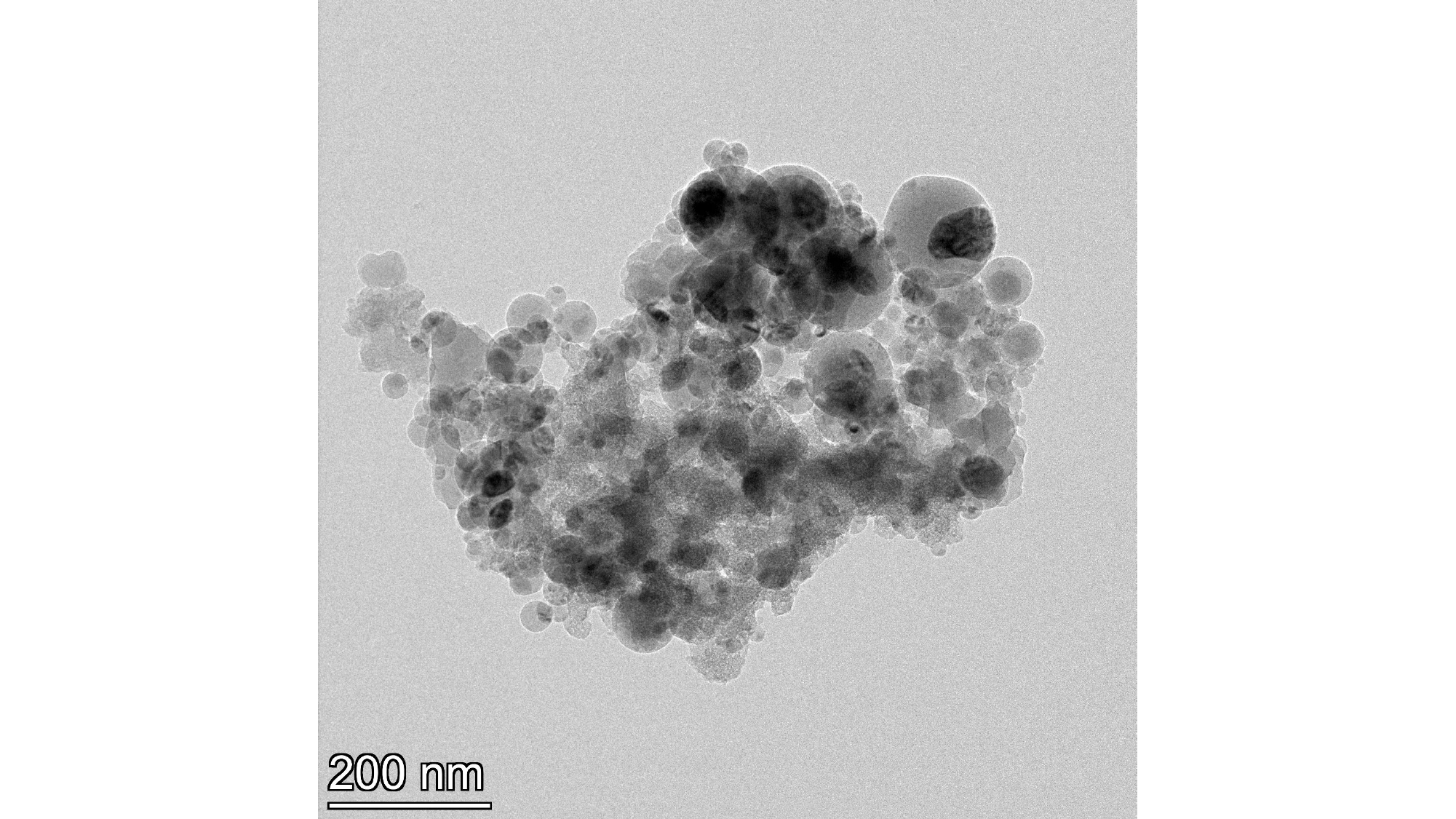

## Slide 38
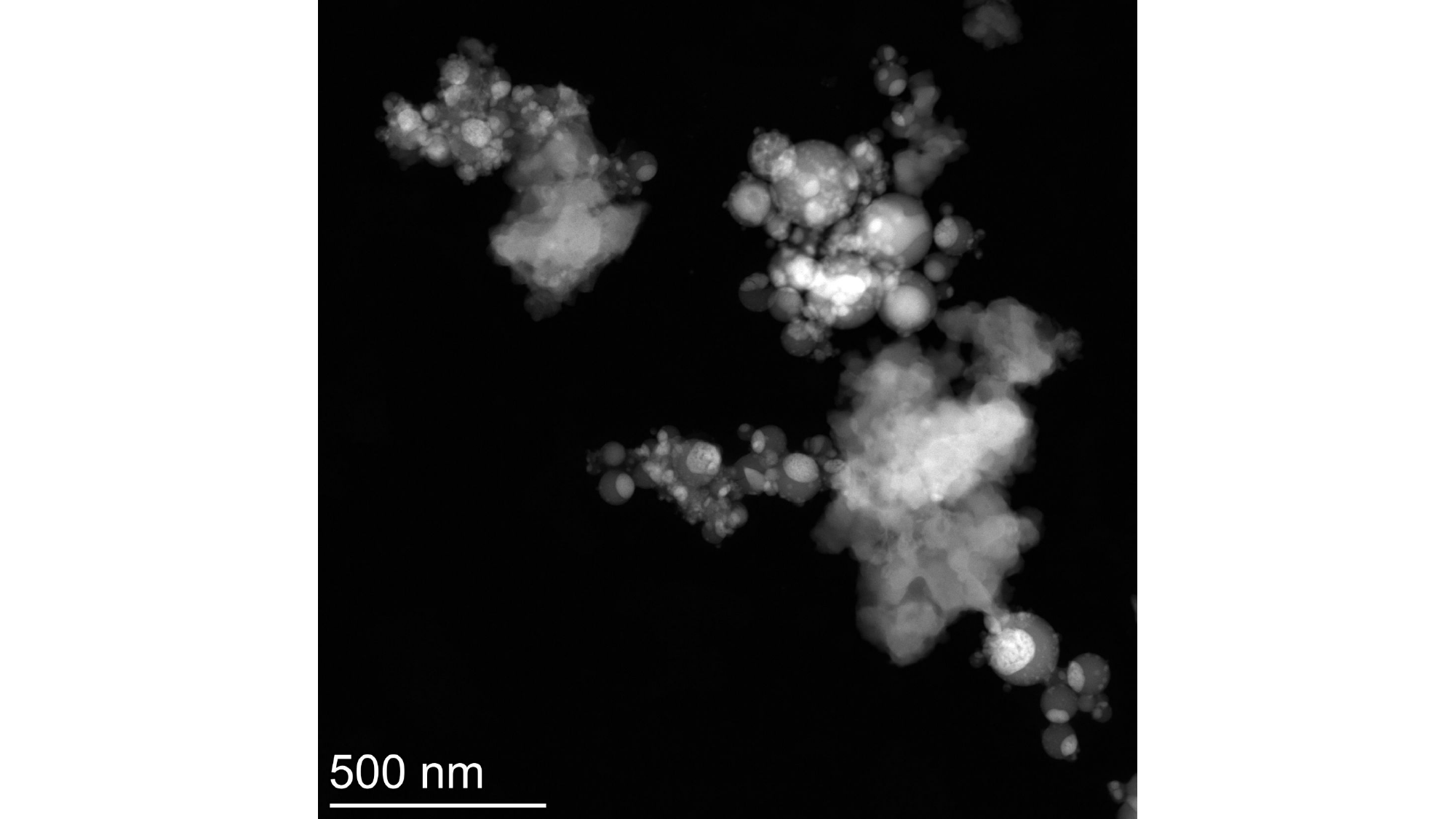

## Slide 39
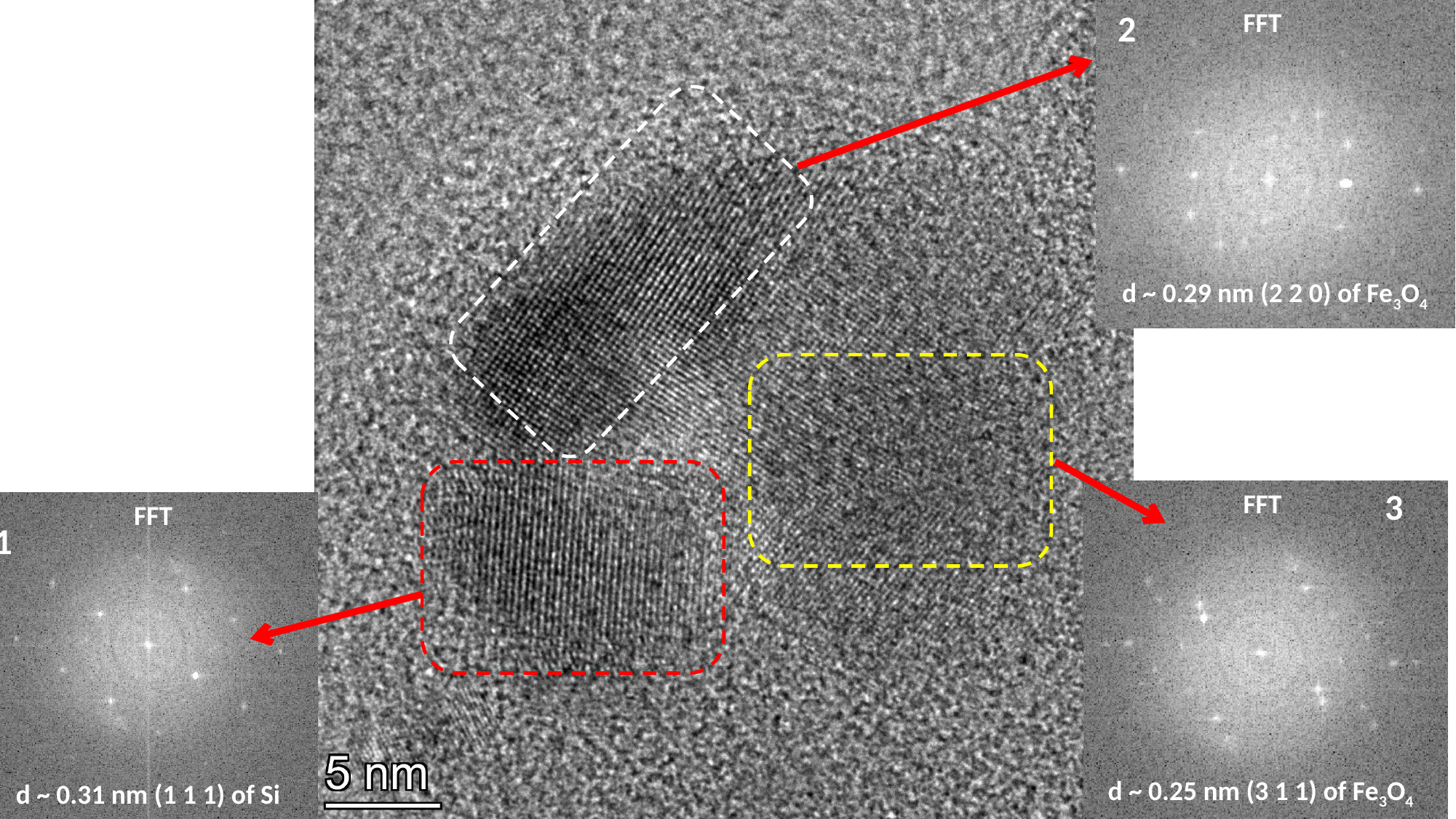

FFT
d ~ 0.29 nm (2 2 0) of Fe3O4
FFT
FFT
d ~ 0.25 nm (3 1 1) of Fe3O4
d ~ 0.31 nm (1 1 1) of Si
2
3
1

## Slide 40
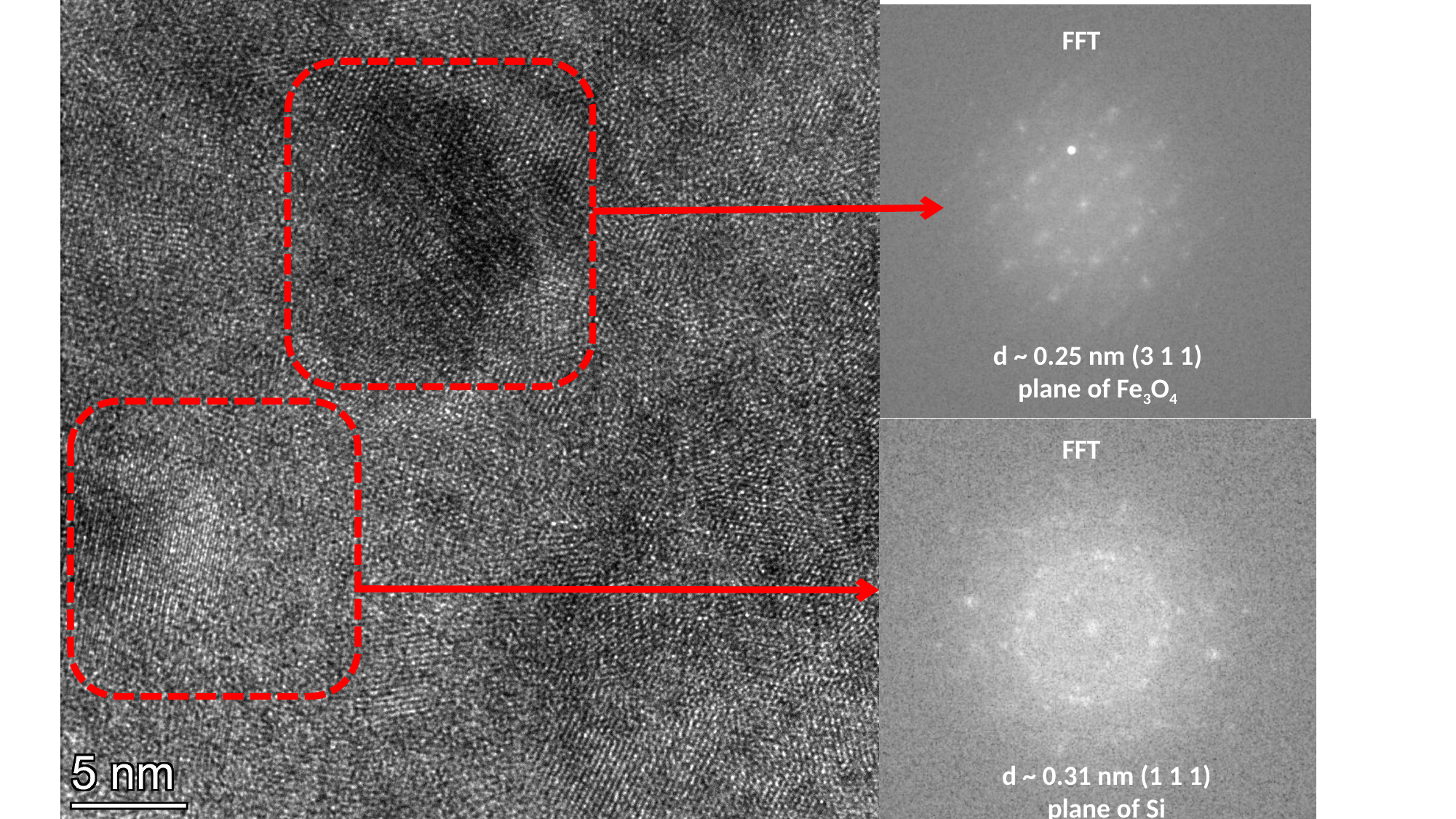

FFT
d ~ 0.25 nm (3 1 1) plane of Fe3O4
FFT
d ~ 0.31 nm (1 1 1) plane of Si

## Slide 41
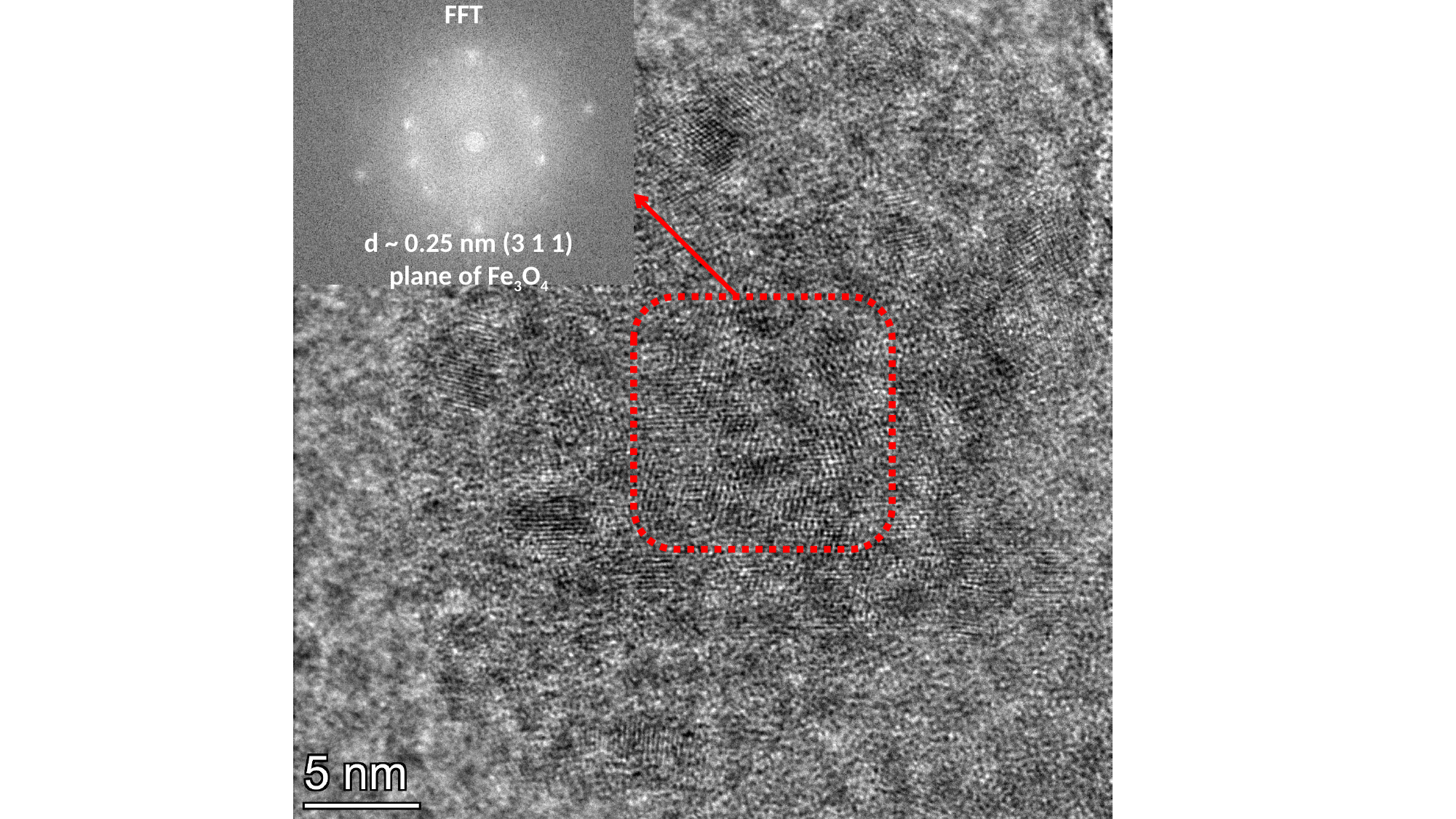

FFT
d ~ 0.25 nm (3 1 1) plane of Fe3O4

## Slide 42
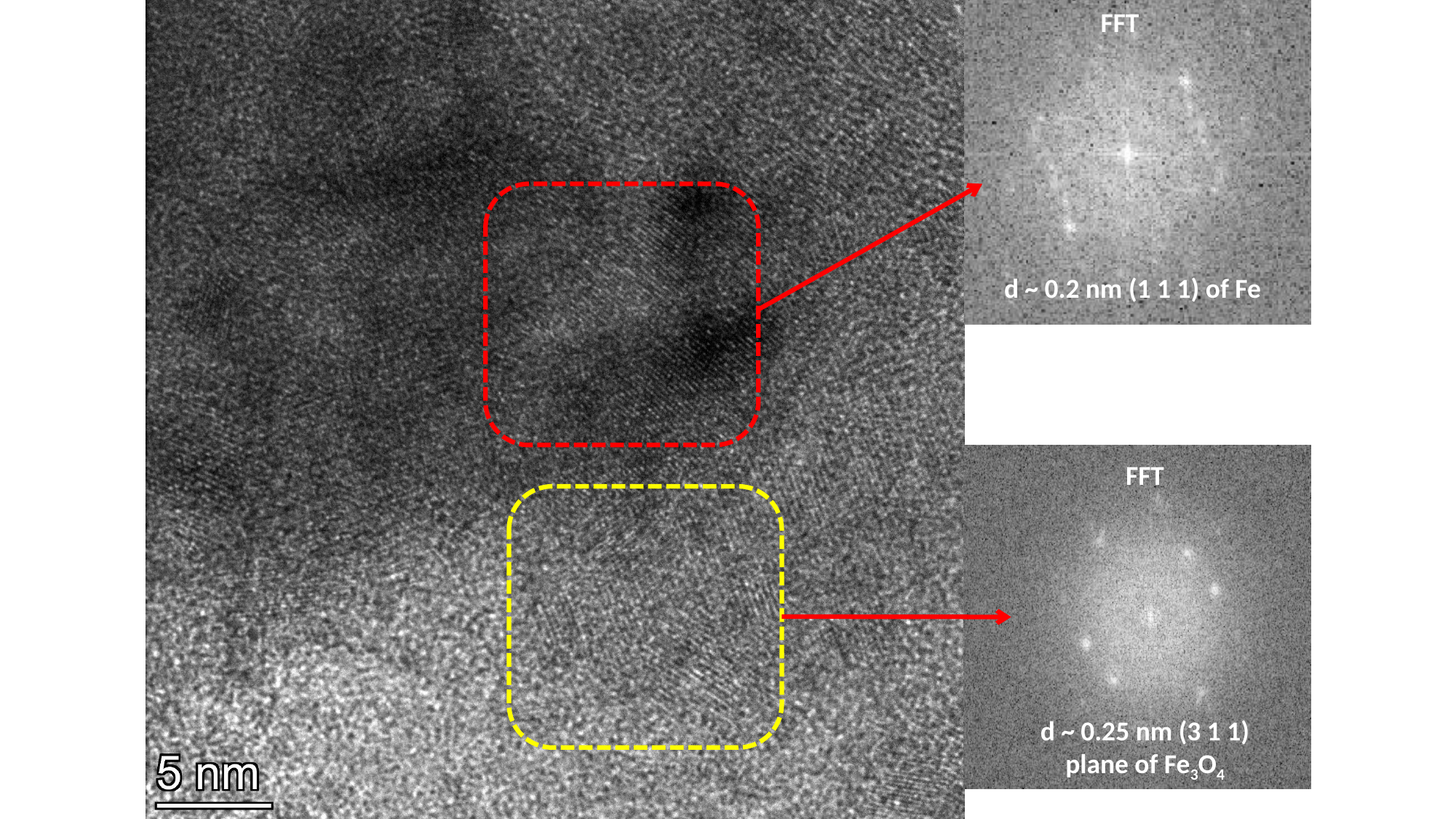

FFT
d ~ 0.2 nm (1 1 1) of Fe
FFT
d ~ 0.25 nm (3 1 1) plane of Fe3O4
