# Supplementary material for: Instantaneous formation of interstellar minerals and mineral quantum dots
Source: RSC Adv. 2025 Apr 17;15(16):12309–20. doi: 10.1039/d5ra01088h (PMC12004361; doi:10.1039/d5ra01088h)
Supplement: RA-015-D5RA01088H-s002 [file RA-015-D5RA01088H-s002.pdf]

Supplementary Information (SI) for RSC Advances.
This journal is © The Royal Society of Chemistry 2025

Supplementary Materials:

Table S1: Relative comparison between the IR active peaks present in shocked sample of M1 and the IR active peaks of Forsterite (Mg_2SiO_4) from Jäger et al. 1998.

Peak position (µm) Shocked Sample (M1)	Peak Position of Forsterite (µm) (Jäger et al. 1998)	Assignment
9	9.3	
9.8	10	**Asymmetric stretching of Si-O bond**
10.1	10.2	**do**
10.4	10.4	**do**
11.1	11.2	
11.9	11.9	**Symmetric stretching of Si-O bond**
16.2	16.3	**Asymmetric bending of Si-O-Si bond**
19.7	19.5	**Symmetric bending of Si-O-Si**
23.8	23.5	**Translation of Mg^{2+}**
25	24.7	
27.8	27.5	
33	33.5	**Translation of one Metal cation**

Table S2: Relative comparison between the IR active peaks present in the shocked sample of M2 and the IR active peaks of natural olivine ($Mg_{1.8}Fe_{0.2}SiO_4$) from Jäger et al.1998.

Peak position (µm) Shocked Sample (M2)	Peak Position of Natural Olivine (µm) (Jäger et al. 1998)	Assignment
9		
9.9	10	**Asymmetric stretching of Si-O bond**
10.2	10.2	**do**
10.4	10.5	**do**
11.3	11.3	**do**
11.9	11.9	**Symmetric stretching of Si-O bond**
16.4	16.4	**Asymmetric bending of Si-O-Si bond**
18.9	18.3	
19.8	19.5	**Symmetric bending of Si-O-Si**
21.5	21.6	**Rotation of SiO_4**
23.9	23.9	**Translation motion of metal cation**
25.3	25.3	
33	33.5	**Translation motion of metal cation**

Table S3: XRD peaks observed in the shocked sample of M1, along with their corresponding d-spacing values and assignments to different crystalline phases (Forsterite, MgO, Mg, and Si).

			Assignment (Millar indices (d spacing in Å))			
**2 θ (deg)**	**θ**	**d spacing (Å)**	**Forsterite (Mg_2SiO_4)**	**MgO**	**Mg**	**Si**
			**CODID: 9000319**	**CODID:1000053**	**CODID: 9008506**	**CODID: 1526655**
17.65	8.83	5.02	**2 0 0 (5.10)**			
23.12	11.56	3.84	**0 2 1 (3.88)**			
24.13	12.07	3.69	**1 0 1 (3.72)**			
25.74	12.87	3.46	**1 1 1 (3.49)**			
28.76	14.38	3.1				**1 1 1 (3.10)**
30.05	15.03	2.97	**0 0 2 (2.99)**			
32.43	16.22	2.76	**1 3 0 (2.76)**			
32.57	16.29	2.75			**1 0 0 (2.77)**	
34.63	17.32	2.59			**0 0 2 (2.60)**	
35.97	17.99	2.49	**1 3 1 (2.51)**			
36.82	18.41	2.44	**1 1 2 (2.45)**	**1 1 1 (2.43)**		
37.16	18.58	2.42			**1 0 1 (2.45)**	
38.64	19.32	2.33				
39.92	19.96	2.26	**1 2 2 (2.26)**			
40.32	20.16	2.24	**1 4 0 (2.24)**			
42.04	21.02	2.15	**2 1 1 (2.15)**			
43.15	21.58	2.09		**2 0 0 (2.10)**		
44.86	22.43	2.02				
47.57	23.79	1.91				**2 0 0 (1.90)**
48.04	24.02	1.89			**1 0 2 (1.90)**	
52.49	26.25	1.74	**2 2 2 (1.74)**			
56.44	28.22	1.63				**3 1 1 (1.62)**
57.58	28.79	1.6			**1 1 0 (1.60)**	
62.02	31.01	1.5	**0 0 4 (1.49)**			
62.53	31.27	1.48		**2 2 0 (1.49)**		

62.69	31.35	1.48	**0 6 2 (1.47)**			
63.25	31.63	1.47			**1 0 3 (1.47)**	
68.85	34.43	1.36			**1 1 2 (1.36)**	
70.16	35.08	1.34	**3 4 0 (1.34)**			
71.19	35.6	1.32	**1 3 4 (1.31)**			
73.05	36.53	1.29	**0 6 3 (1.29)**			
74.89	37.45	1.27		**3 1 1 (1.27)**		
78.81	39.41	1.21		**2 2 2 (1.21)**		

Table S4: XRD peaks observed in the shocked sample of M2, along with their corresponding d-spacing values and assignments to different crystalline phases (Olivine, MgO, Mg, and Fe).

			Assignment (Millar indices (d spacing in Å))			
2θ (deg)	θ (deg)	d (Å)	Olivine ($Mg_{1.8}Fe_{0.2}SiO_4$)	MgO	Mg	Fe
			CODID:1010497	CODID:1000053	CODID: 9008506	
17.41	8.71	5.09	**2 0 0 (5.10)**			
22.94	11.47	3.87	**2 0 1(3.88)**			
23.97	11.99	3.71	**0 1 1 (3.72)**			
25.56	12.78	3.48	**3.49 (1 1 1)**			
29.93	14.97	2.98	**0 2 0 (2.99)**			
32.31	16.16	2.77	**3 0 1 (2.76)**		**1 0 0 (2.77)**	
34.49	17.25	2.6			**0 0 2 (2.60)**	
36.68	18.34	2.45	**1 2 1 (2.45)**		**1 0 1 (2.45)**	
36.89	18.45	2.43		**1 1 1 (2.43)**		
38.51	19.26	2.34	**4 1 0 (2.34)**			
39.72	19.86	2.27	**2 2 1 (2.26)**			
40.17	20.09	2.24	**4 0 1 (2.24)**			
41.85	20.93	2.16	**1 1 2 (2.15)**			
43	21.5	2.1		**2 0 0 (2.10)**		
44.47	22.24	2.04				**1 1 1 (2.01)**
45.48	22.74	1.99				
47.93	23.97	1.9				
52.43	26.22	1.74	**2 2 2 (1.74)**			
52.76	26.38	1.73	**4 0 2 (1.73)**			
56.96	28.48	1.62	**6 1 0 (1.63)**			
57.43	28.72	1.6			**1 1 1 (1.6)**	
62.01	31.01	1.5	**0 4 0 (1.49)**			
62.34	31.17	1.49		**2 2 0 (1.49)**		
62.83	31.42	1.48	**6 2 0 (1.48)**			
63.09	31.55	1.47			**1 0 3 (1.47)**	
65.18	32.59	1.43				**2 1 1 (1.44)**
68.74	34.37	1.36			**1 1 2 (1. 36)**	
69.79	34.9	1.35	**2 2 3 (1.35)**			
70.08	35.04	1.34			**2 0 1 (1.34)**	
77.8	38.9	1.23	**1 3 3 (1.23)**			
78.76	39.38	1.21		**2 2 2 (1.21)**		
82.54	41.27	1.17				**2 1 1 (1.17)**

Detailed description of HISTA

The High Intensity Shock tube for Astrochemistry (HISTA) is a gas driven 7 m long shock tube housed at Physical Research Laboratory (PRL), Ahmedabad. The High Intensity Shock Tube for Astrochemistry (HISTA) is a piston less single pulse gas driven shock tube dedicated for the astrochemistry research. This shock tube is capable to mimic the low velocity interstellar shocks, especially shocks observed around Mira variable, pulsation driven shock from AGB stars and impact induced shocks observed in different airless solar system bodies. A schematic diagram of the HISTA is shown in Fig **S.1**. It is a 7 m long, gas-driven shock tube that has two different sections separated by a metal (Aluminium) diaphragm. The high pressure (driver) section is 2 m long whereas the low-pressure (driven) section is 5 m long, and can be extended by another 3 m. Both ends of the HISTA are usually closed using thick metal (steel) flanges, serving as a shock reflector and shock absorber. The entire shock tube is made of stainless steel and mounted horizontally with the help of metallic mount. The inner diameter of the HISTA is around 82 mm and the outer diameter is around 115 mm. The driven section of HISTA is equipped with two piezoelectric pressure sensors separated by 30 cm. Also a pumping system is connected to the both driven and driver sections. The sample is placed at the end of the shock driven section and spread across the inner walls of the tube.

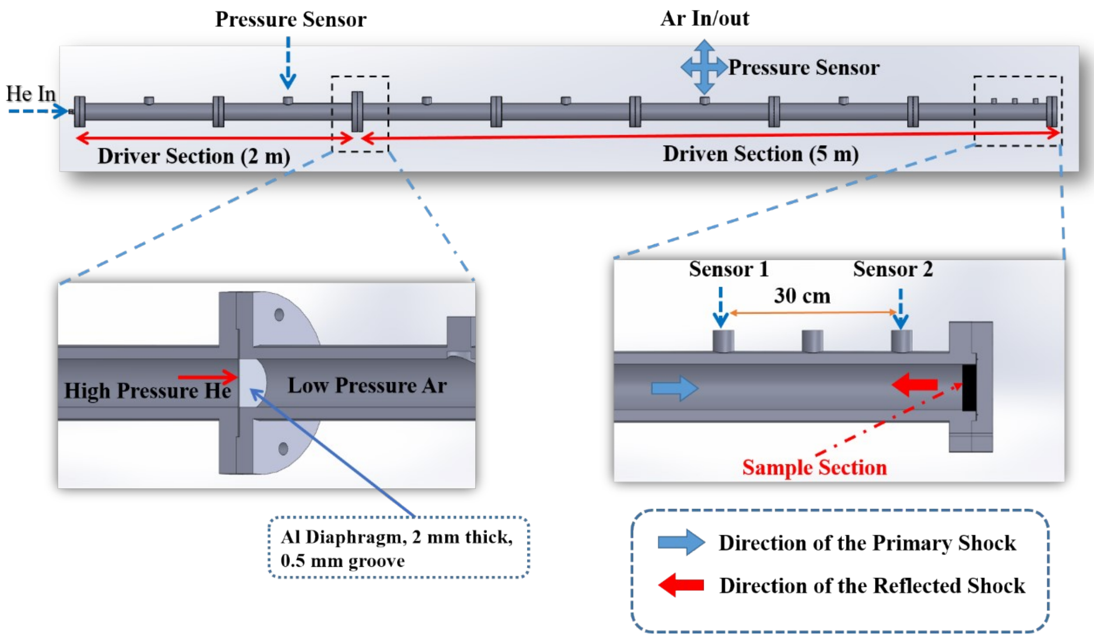

Fig S.1: The schematic diagram of HISTA housed at PRL, Ahmedabad, India. The driver section contains high pressure Helium and the driven section usually filled with low pressure Argon. The sample to be tested is loaded at the end of the shock tube, highlighted in black. Two Piezo electric dynamic-pressure sensors, labelled as Sensor 1 and Sensor 2, are positioned 30 cm apart at the end of the driven section to measure shock speed.

Before every experiment, the entire inner wall of the HISTA thoroughly cleaned using ethanol to minimise any possible chances of contaminations from the previous experiment. About 0.1 gm of powder sample is used in every experiment and once the sample is placed, we immediately lock the entire shock tube with the help of two end flanges. With the help of two scroll pump we pumped down both the driver and the driven section up to 10^{-3} mbar level. This is followed by purging of the entire driven and driver section with Ar and He. This further reduce the level of contamination present in the shock tube such as residual gas. In this work we kept the driven section with 0.1 Bar Ar for all the experiments. The Al diaphragm plays a crucial role in the shock formation process. It is a 2 mm thick diaphragm but grooved with different depths, which plays a crucial role in the shock formation pressure. In this work, we used 0.5 mm groove depth diaphragm. For the shock generation we filled the driver section with high pressure He very rapidly. At about 68 bar of driver pressure, the diaphragm ruptures and this allows the high pressure He to expand in the low pressure Ar medium and leads to the formation of shock waves. This shock wave is called primary shock wave which travels through the driven section and reflected back from the driven section end flange and therefore called as reflected shock wave. The sample placed at the end of the driven section are processed by both the primary and reflected shock waves. For the shock speed measurement, we used two PCB PIEZOTRONICS dynamic pressure sensors (Voltage sensitivity 0.4949 mV/PSI, Bias voltage 10.71 V). High intensity shocks may induce physio-chemical changes in the sample and it is very likely that a significant amount of the powder sample would have transformed into the gas phase after the processing. To avoid any loss of processed sample we left the entire shocked gas alone for a couple of hours so as to settle the residual sample inside the shock tube. During the settle down period, the internal static pressure of HISTA was around 18 bar, we then slowly removed the shocked gas to the atmosphere using a leak valve. Once the shock tube's internal pressure became equal to the atmospheric pressure we opened the end flange of the driven section and collected the rest of the processed samples in the vicinity to the end flange area.

Measurement and calculation of different shock parameters

In the shock tube, most of the thermodynamic parameters are usually expressed in terms of the Mach number (M). As explained in the previous section, two piezoelectric pressure sensors provide the pressure response created because of the arrival of the incident shock, and the pressure response curve is recorded in a digital oscilloscope. A typical shock pressure response curve is shown in **Fig S.2**. The velocity of the shock wave (Vs) calculated by determining the time taken (Δt) for the shock wave to travel the distance (Δx) between the pressure sensors, using the recorded pressure signal as shown in Figure below. Once the shock velocity is calculated, one can calculate the Mach number of the shock wave using the following relation:

$$Mach\ Number\ (M1)\ = \frac{v_S}{a}$$

Where a is the local speed of sound. By employing the calculated value of M1 to a set of equations , known as Rankin- Hugoniot jump equation, described below one can estimate the reflected shock temperature (T_5) and pressure (P_4).

$$\frac{T_5}{T_1} = \frac{\left[(2\gamma - 1)M_1^2 + (3 - \gamma)\right]\left[(3\gamma - 1)M_1^2 - 2(\gamma - 1)\right]}{(\gamma + 1)^2 M_1^2}$$

$$\frac{P_5}{P_1} = \left(\frac{2\gamma M_1^2 - (\gamma - 1)}{\gamma + 1}\right)\left[\frac{(3\gamma - 1)M_1^2 - 2(\gamma - 1)}{(\gamma - 1)M_1^2 + 2}\right]$$

Here the P_1 and T_1 are the initial pressure and temperature of the driven sections, and these are 0.1 bar and 300 K, $\gamma = 1.67$ for Argon and $M_1 = 5.6$. The estimated T_5 is around 7300 K.

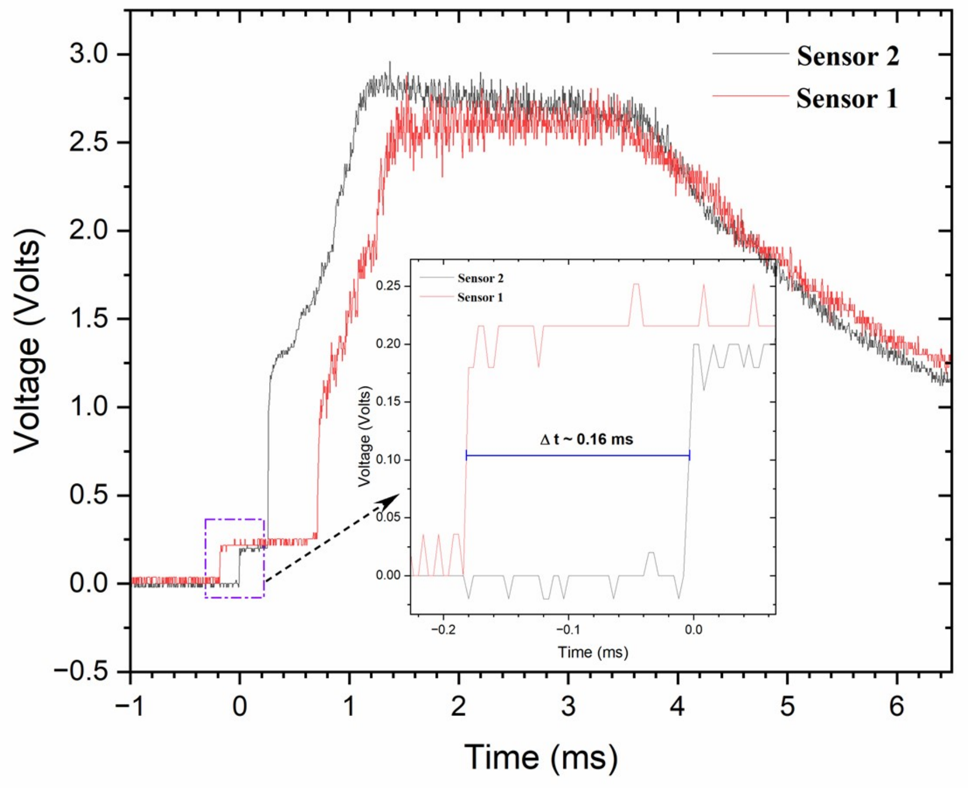

Fig S.2: A typical shock response curve of HISTA was recorded on a digital oscilloscope using two piezoelectric pressure sensors, PS1 and PS2, positioned at the end of HISTA. The inset highlights the time delay between the pressure signals from PS1 and PS2 during the arrival of the incident shock.

FE-SEM Analysis

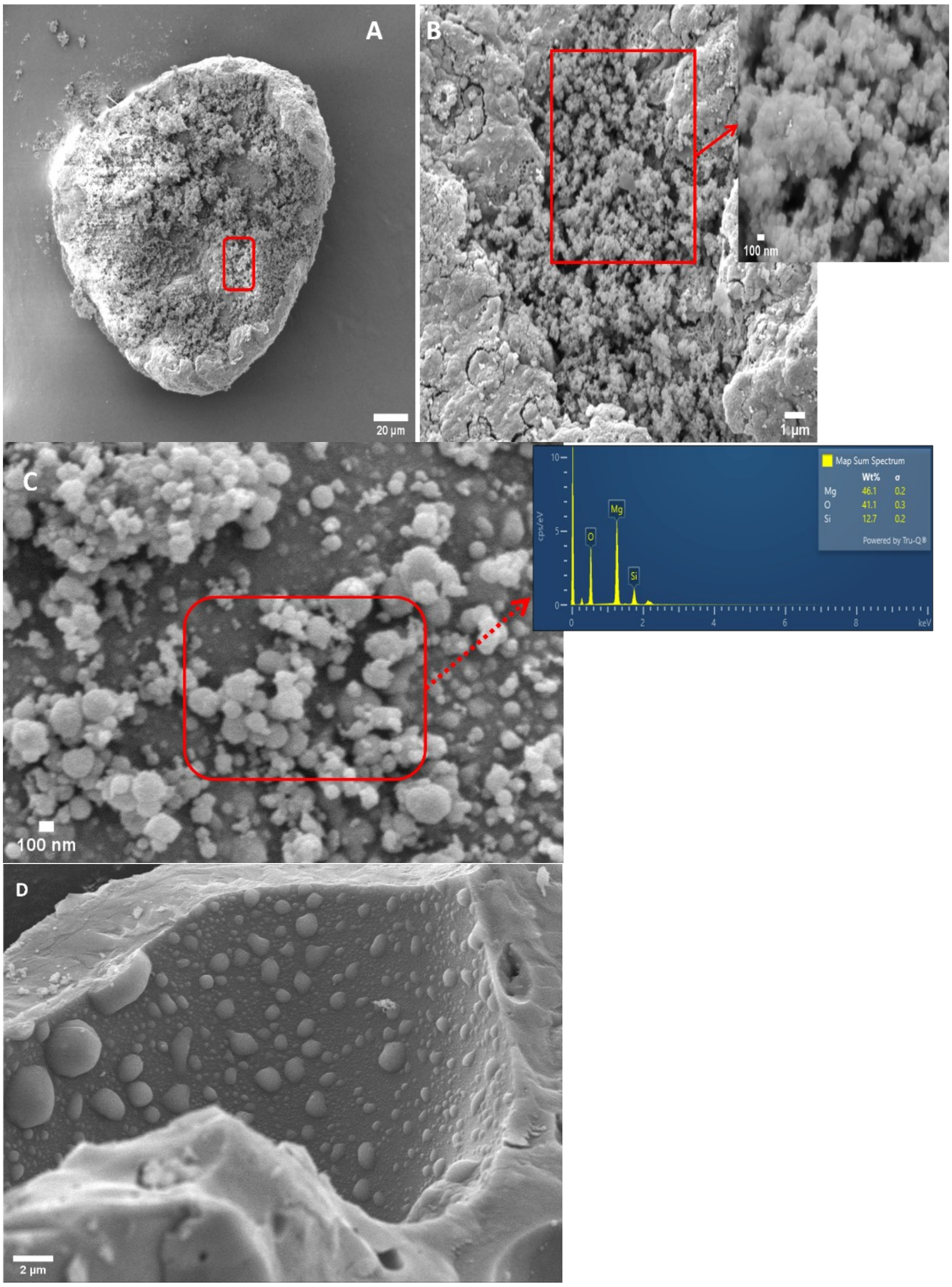

Fig S3: Supplementary FE-SEM images of the unshocked and shocked sample of M1. (**A**) FE-SEM images of the unshocked sample of M1shows a micron size large particle. (**B**) Zoomed view of the region marked by red box in (**A**) shows the sub-micron to nm size particle with different shapes and size. (**C**) FE-SEM images of the shocked sample of M1 and the inset shows the EDX spectra of the shock produced spherical particles. (**D**) shows the piece of a broken spherical grain in the shocked sample of M1, suggesting collisions during rapid cooling amongst the spherical grains with different particle sizes.

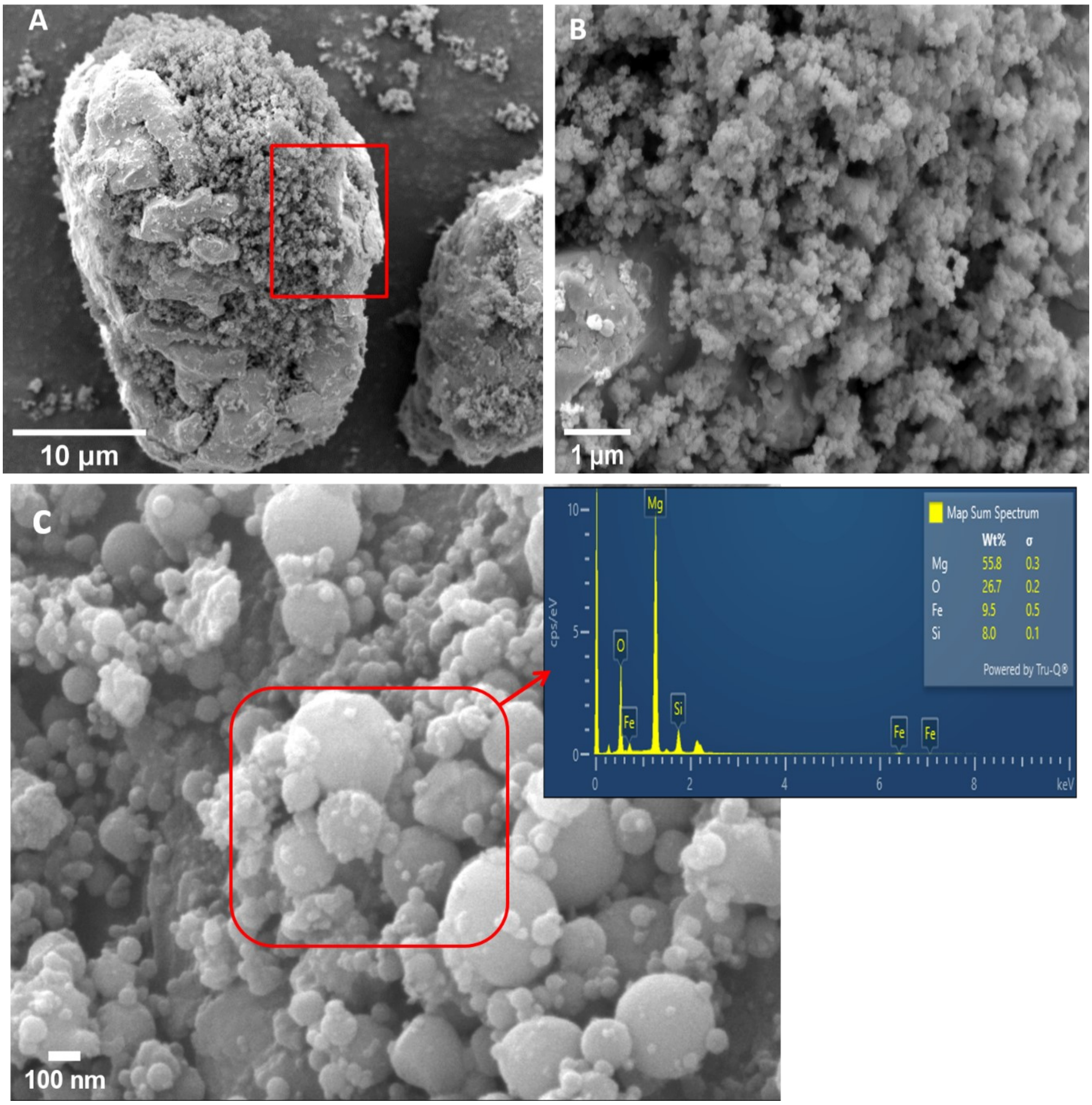

Fig S4: FE-SEM images of the unshocked and shocked samples of M2. (**A**) is showing a large grain of the unshocked sample, and (**B**) represents the close view of the area marked by a red box in (**A**), which contains small dust grains of different shapes and sizes. (**C**) is showing the FE-SEM image of the shocked M2 sample and the inset shows the EDX spectra of the spherical grains with in the red box.

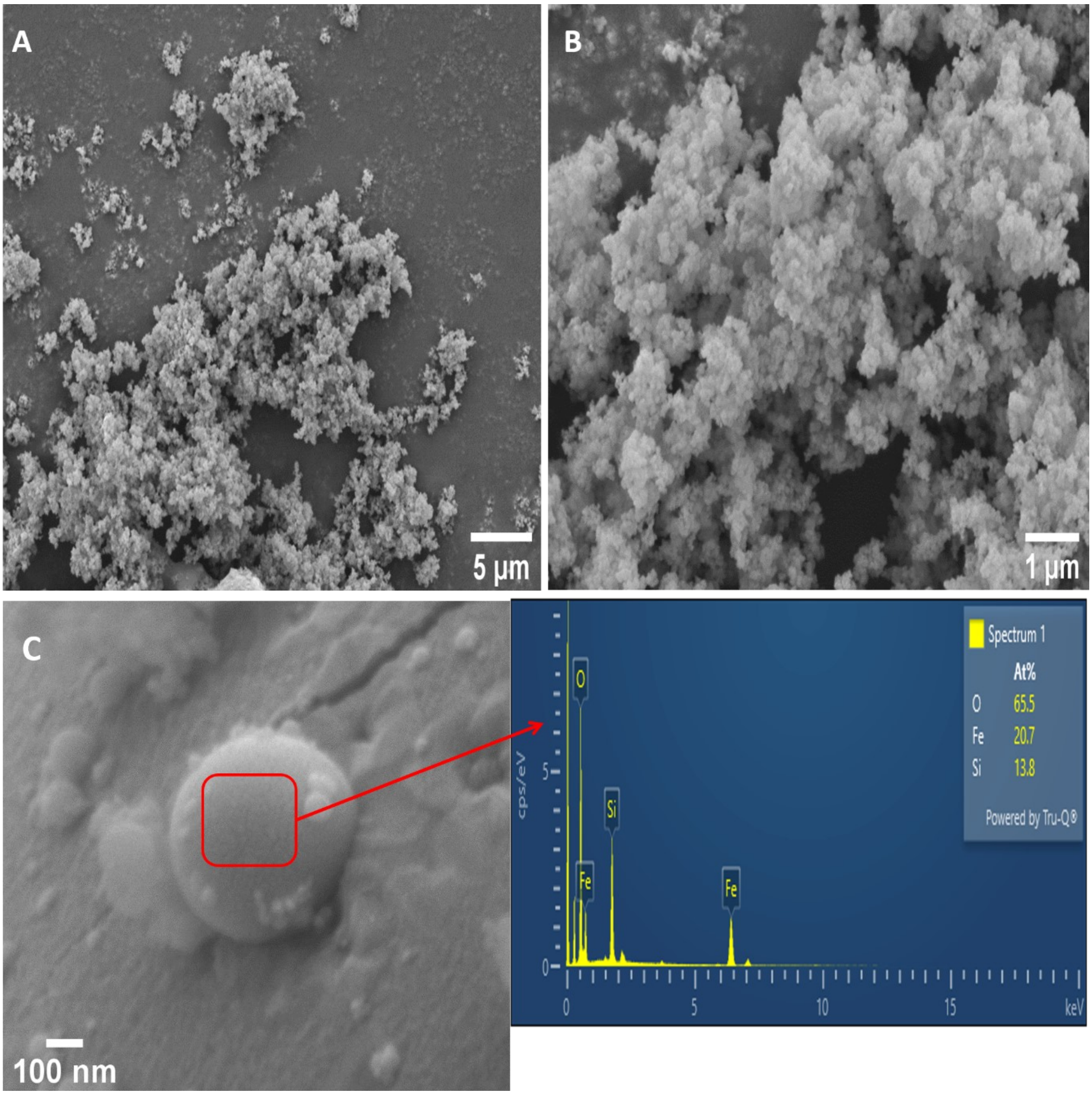

Fig S5: FE-SEM images of the unshocked and shocked samples of M3. (**A**) unshocked samples with scale size 5 µm (**A**), and, (**B**) shows the unshocked samples with scale size 1 µm. (**C**) FE-SEM image of the shocked produced spherical particle with its EDX spectra indicated using a red arrow.

HR-TEM Analysis

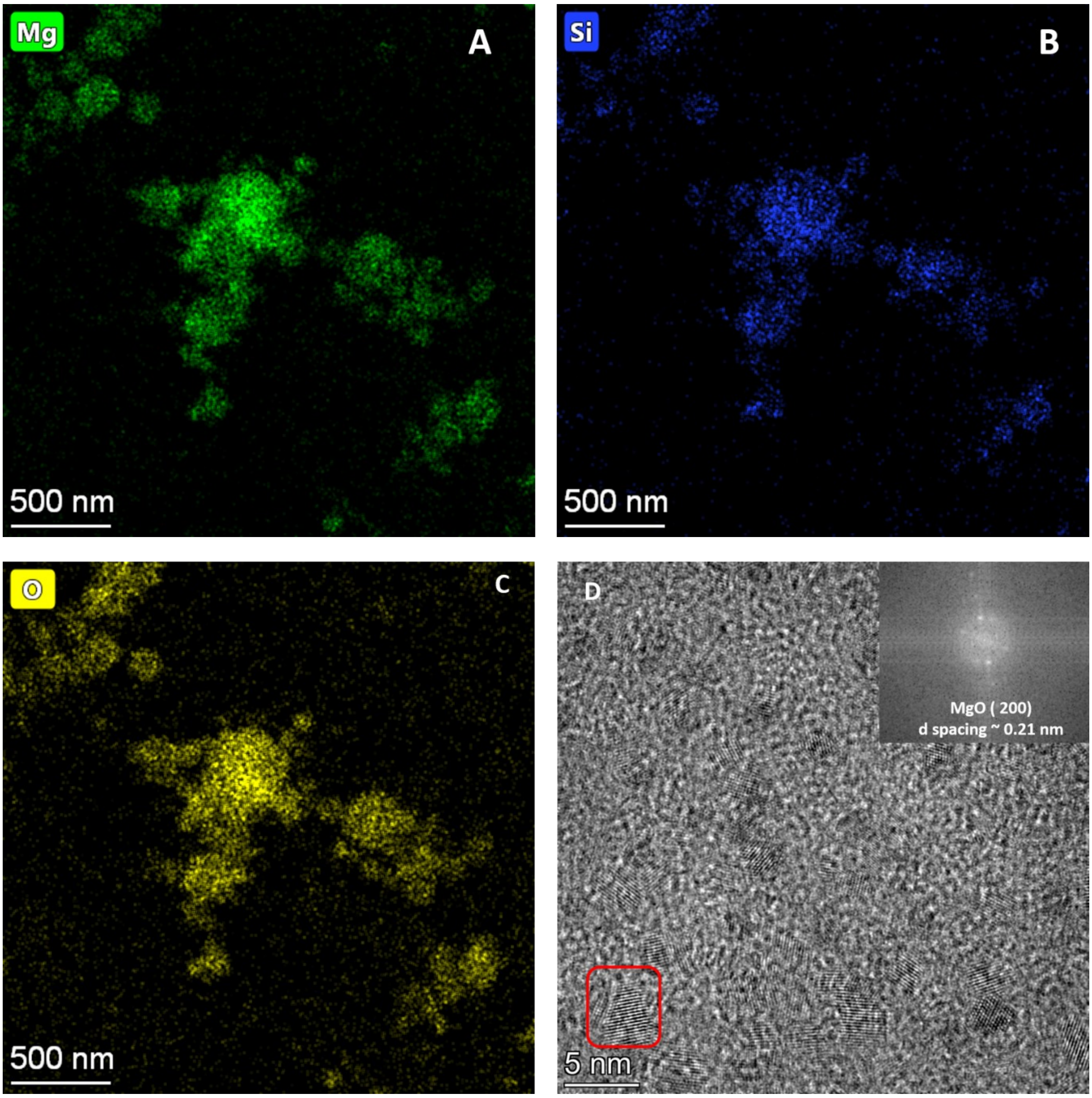

Fig. S6: Elemental mapping of the spherical particle in the shocked M1 sample, shown in Fig. 4.A of the main manuscript, along with the quantum dots present in the shocked M1 sample. (A) Distribution of Mg, (B) distribution of Si, and (C) distribution of O in the spherical dust particles. (D) MgO quantum dots present in the shocked sample.

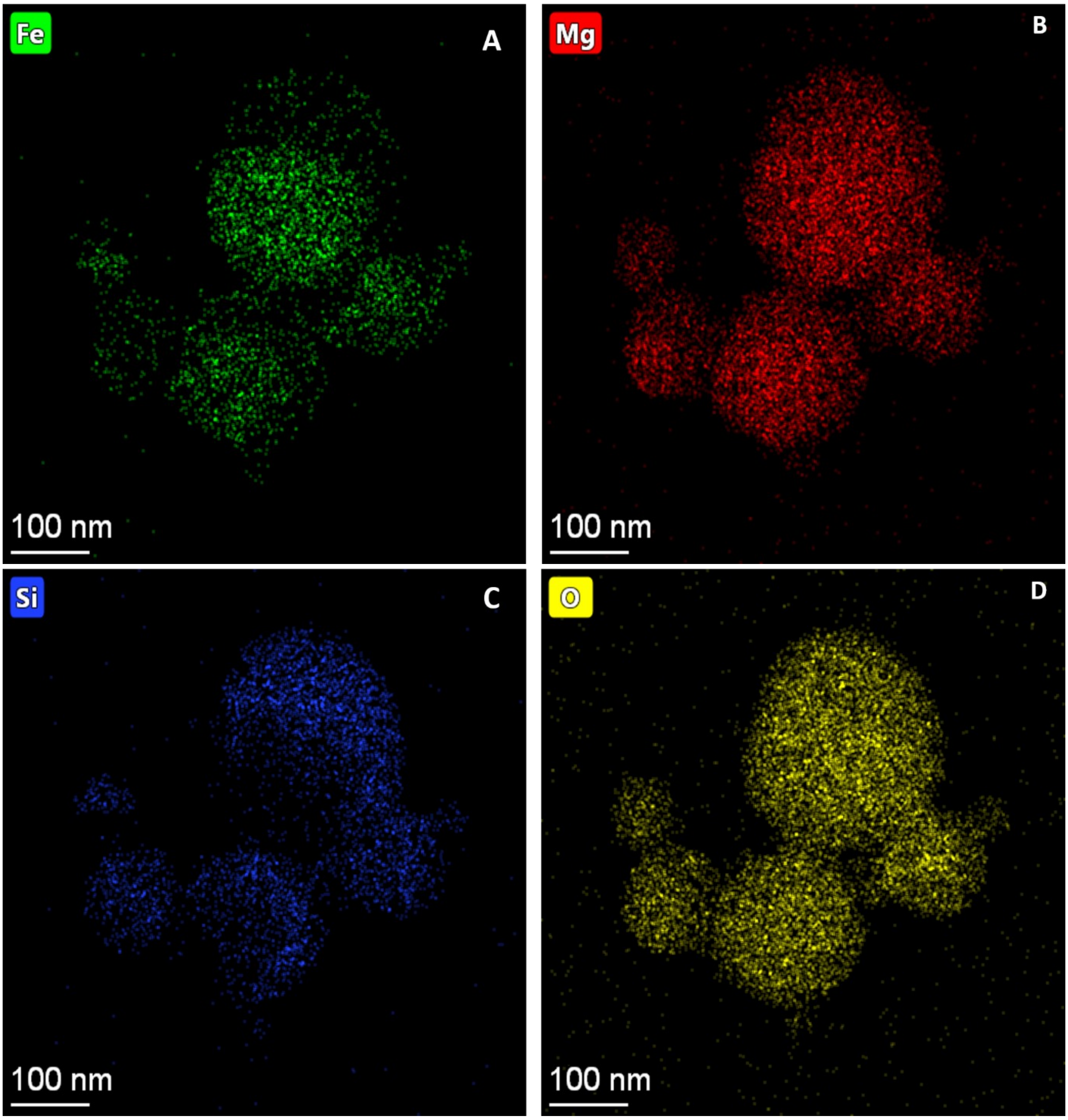
Fe
A
100 nm
Mg
B
100 nm
Si
C
100 nm
O
D
100 nm

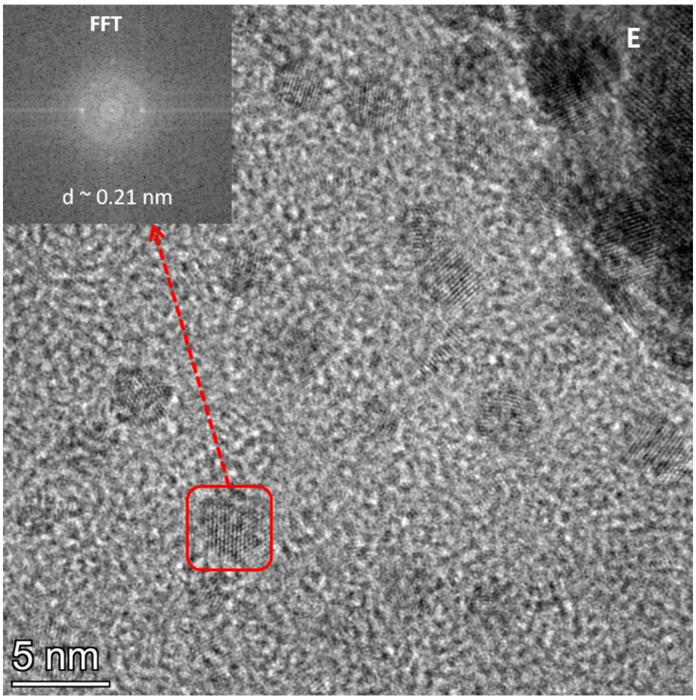

Fig S7: Elemental mapping of the shocked produced spherical particle showed in Fig 5.A and the quantum dots present in the shocked M2 sample. (**A**) shows the distribution of Fe. (**B**) shows the distribution of Mg. (**C**) shows the distribution of Si and (**D**) shows the distribution of O in the spherical dust particles. (**E**) MgO quantum dots present in the shocked sample.
